# Alteration of the microRNA network during the progression of Alzheimer's disease

**DOI:** 10.1002/emmm.201201974

**Published:** 2013-09-09

**Authors:** Pierre Lau, Koen Bossers, Rekin's Janky, Evgenia Salta, Carlo Sala Frigerio, Shahar Barbash, Roy Rothman, Annerieke S R Sierksma, Amantha Thathiah, David Greenberg, Aikaterini S Papadopoulou, Tilmann Achsel, Torik Ayoubi, Hermona Soreq, Joost Verhaagen, Dick F Swaab, Stein Aerts, Bart De Strooper

**Affiliations:** 1 VIB Center for the Biology of DiseaseLeuven, Belgium; 2 Center for Human Genetics, Leuven Institute for Neurodegenerative Disorders (LIND) University Hospitals Leuven, and University of LeuvenO&N4, Herestraat, Leuven, Belgium; 3 Neurogeneration Group, Netherlands Institute for Neuroscience, an Institute of the Royal Netherlands Academy of Arts and SciencesAmsterdam, The Netherlands; 4 Laboratory of Computational Biology, Center for Human Genetics and University of LeuvenO&N4, Herestraat, Leuven, Belgium; 5 Department of Biological Chemistry, the Silberman Institute of Life Sciences, and the Edmond and Lily Safra Center of Brain Science Interdisciplinary Center for Neural ComputationJerusalem, Israel; 6 Saint James School of MedicinePlaza Juliana, Kralendijk, Bonaire, Dutch Caribbean, The Netherlands; 7 Neuropsychiatric Disorders Group, Netherlands Institute for Neuroscience, an Institute of the Royal Netherlands Academy of Arts and SciencesAmsterdam, The Netherlands

**Keywords:** Alzheimer's disease, hippocampus, prefrontal cortex, microRNA, miR-132-3p

## Abstract

An overview of miRNAs altered in Alzheimer's disease (AD) was established by profiling the hippocampus of a cohort of 41 late-onset AD (LOAD) patients and 23 controls, showing deregulation of 35 miRNAs. Profiling of miRNAs in the prefrontal cortex of a second independent cohort of 49 patients grouped by Braak stages revealed 41 deregulated miRNAs. We focused on miR-132-3p which is strongly altered in both brain areas. Downregulation of this miRNA occurs already at Braak stages III and IV, before loss of neuron-specific miRNAs. Next-generation sequencing confirmed a strong decrease of miR-132-3p and of three family-related miRNAs encoded by the same miRNA cluster on chromosome 17. Deregulation of miR-132-3p in AD brain appears to occur mainly in neurons displaying Tau hyper-phosphorylation. We provide evidence that miR-132-3p may contribute to disease progression through aberrant regulation of mRNA targets in the Tau network. The transcription factor (TF) FOXO1a appears to be a key target of miR-132-3p in this pathway.

## INTRODUCTION

Alzheimer's disease (AD) is a progressive age-related dementia characterized by amyloid plaques, neuronal tangles, neurofibrillary degeneration and vascular amyloidopathy in the brain. The cause of early-onset AD (EOAD) is well known with mutations in the APP, PSEN1 and PSEN2 genes, contributing to the accumulation of amyloid plaques. In late-onset AD (LOAD), genome-wide association studies (GWAS) recently identified more than ten other *loci* [reviewed in (Tanzi, 2012)], suggesting the existence of additional molecular pathways contributing to the disease. MicroRNAs (miRNAs) are short ∼22 nt RNA molecules that bind to the transcripts of protein-coding genes to direct their post-transcriptional repression and by that regulate important physiological and pathophysiological signalling pathways. Increasing evidence links aberrant expression of miRNAs to neurodegenerative disorders including AD [reviewed in (Lau & de Strooper, 2010)]. For instance, miR-29b was found to be downregulated in the anterior temporal cortex of a subgroup of AD patients with high BACE1 protein expression (Hebert et al, 2008) and decreased miR-107 was also observed in the temporal cortex of some AD cases (Wang et al, 2008). However, the small number of patients analyzed represents a major issue when identifying which miRNAs are deregulated during disease. Confounding factors, in particular the technologies used for miRNA profiling which provide deviating results (Pritchard et al, 2012), have to be considered as well.

A more systematic analysis of miRNAs is clearly needed to determine which miRNAs and molecular networks normally controlled by such miRNAs are affected during disease [reviewed in (Salta & De Strooper, 2012)]. We aimed here to determine miRNA alterations in LOAD by profiling two large and independent cohorts of patients using the nCounter system, a technology proven to reliably quantify miRNAs (Wyman et al, 2011). We found numerous changes in the expression of miRNAs in the hippocampus and prefrontal cortex of LOAD patients. Of importance, downregulation of miR-132-3p stands out by robustness and consistency. This observation was confirmed by real-time PCR on the two same brain areas initially investigated and for the temporal gyrus. In agreement, downregulation of miR-132-3p in the LOAD prefrontal cortex was also found by next-generation sequencing of miRNAs and by *in situ* hybridization. We also provide initial identification of miR-132-3p targets of relevance to LOAD, thus offering novel insights into the pathogenesis of the disease.

## RESULTS

### Deregulation of miRNAs in the hippocampus of LOAD patients

**Figure 1:**
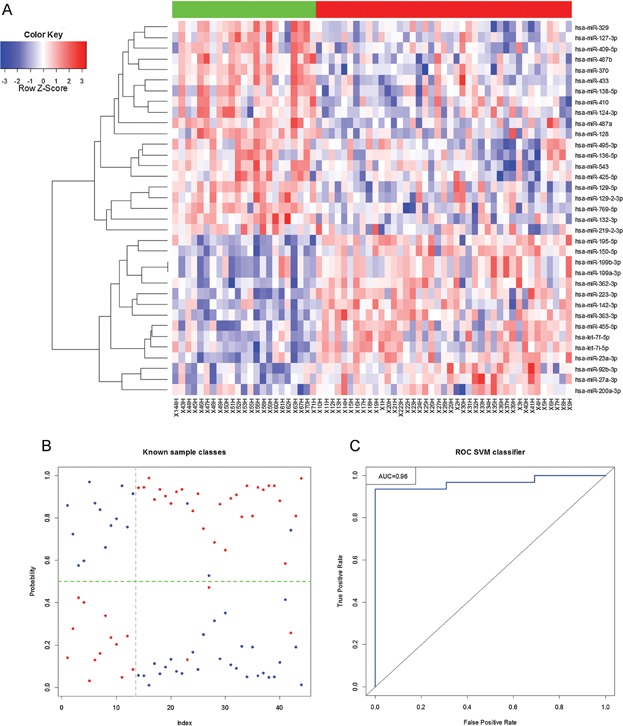
Differential expression of miRNAs in late-onset AD (LOAD) hippocampus Hierarchical clustering of deregulated miRNAs. Thirty-five significant miRNAs (*p* < 0.05, nbinomTest after BH adjustment) obtained from 64 hippocampus samples were hierarchically clustered using Spearman's correlation. Horizontal bar above the heatmap indicates the control (green) and LOAD (red) groups as defined by pathologic examination. The sample identifiers are indicated at the bottom of the heatmap and miRNAs are found on the right side. Blue colour corresponds to low miRNA expression values and red denotes high expression values as indicated by the colour key.Probability prediction of the hippocampus samples. The Support Vector Machine (SVM) training algorithm was built on a subset of 10 LOAD and 10 controls considering the 35 differentially expressed miRNAs shown in panel A. The obtained predictors were used to test the remaining 31 LOAD cases and 13 control samples. The index corresponds to each patient and the vertical dashed line delineates the control (left) and LOAD (right) groups. The y-axis represents the probability of diagnosis for each of the samples as determined by the SVM classifier (predicted class). Blue and red dots correspond to the probability that the sample belongs to the control group or the LOAD group, respectively. A sample is considered as misclassified when the probability for the predicted class is higher than and different from the probability corresponding to the real class (as defined by clinical diagnosis). By using this binary SVM classifier, three LOAD samples were misclassified as controls and all the controls were correctly called.Receiver operating characteristic (ROC) curve of the hippocampus-derived SVM classifier. The ROC curve summarizes the accuracy of the SVM built on the 35 differentially expressed miRNAs using a number of cross-validation equals to the number of predictions (*n* = 44). This set of miRNAs was predictive of LOAD with a sensitivity of 90% and a specificity of 100%. The area under the curve (AUC) was 0.96, with the best possible value would be one and any non-random prediction would be more than 0.5. Following standard convention, the true positive rate is defined as sensitivity and the false positive rate as (1-specificity).

We analyzed the miRNA expression profile of a first cohort made of 41 LOAD cases and 23 age-matched controls (clinical data in Supporting Information Table S1). The quality of the total RNA obtained from these *post-mortem* samples was systematically assessed (Supporting Information Fig S1A). The RNA Integrity Number (RIN) values were relatively low, indicating fragmented total RNA. However, raw data analysis showed that global expression of miRNAs in the samples with lower (2 < RIN < 6) and higher RIN values (RIN > 6) was similar (Supporting Information Figs S1B and S2A), therefore indicating that miRNAs were relatively resistant to RNA degradation. Because of the discrete counting nature of the nCounter system used for miRNA profiling, positive skewness (Supporting Information Fig S2B) and overdispersion of the data (Supporting Information Fig S3B), a statistical model based on the negative binomial distribution as implemented in the DESeq package (Anders & Huber, 2010) was used to call differential miRNA expression. We found that 35 (5.5%) of 641 tested miRNAs were differentially expressed between the LOAD cases and the control group [padj < 0.05, nbinomTest corrected for multiple testing by the Benjamini–Hochberg (BH) procedure]. Of interest, 20 miRNAs were downregulated and 15 were upregulated in the LOAD group when compared to the controls (Table 1 and Fig 1A). Notably, we identified miR-132-3p as the most significantly downregulated miRNA (padj = 1.57E^−07^) together with other brain-enriched miRNAs such as miR-128, miR-136-5p, miR-138-5p, miR-124-3p, miR-129-5p and miR-129-2-3p. Among the upregulated miRNAs in the LOAD group and with previous reported expression in the brain, we found miR-27a-3p, miR-142-3p, miR-92b-3p and miR-200a-3p (Table 1 and Fig 1A).

**Table 1:** Deregulated miRNAs in the hippocampus of LOAD patients

miRNA	Control	LOAD	Fold Change	log2 Fold Change	pval	padj
hsa-miR-132-3p	4316	2541	0.59	−0.76	6.35E-10	1.57E-07
hsa-miR-128	3995	2443	0.61	−0.71	1.42E-07	1.76E-05
hsa-miR-23a-3p	1207	1876	1.55	0.64	1.44E-05	0.0011903
hsa-miR-455-5p	14	40	2.95	1.56	2.72E-05	0.0016864
hsa-miR-129-5p	539	346	0.64	−0.64	3.51E-05	0.0017411
hsa-miR-363-3p	157	251	1.60	0.68	5.96E-05	0.0024648
hsa-miR-27a-3p	129	215	1.67	0.74	0.0001669	0.0059128
hsa-miR-370	92	51	0.55	−0.85	0.0002451	0.0067535
hsa-miR-487b	1860	1321	0.71	−0.49	0.0002444	0.0067535
hsa-let-7f-5p	4040	5712	1.41	0.50	0.0002978	0.0072293
hsa-miR-223-3p	613	885	1.44	0.53	0.0003366	0.0072293
hsa-miR-433	330	213	0.65	−0.63	0.0003498	0.0072293
hsa-miR-195-5p	590	843	1.43	0.51	0.0004041	0.0077096
hsa-miR-138-5p	130	80	0.62	−0.70	0.0004766	0.008442
hsa-miR-142-3p	821	1358	1.66	0.73	0.0005951	0.0098391
hsa-miR-129-2-3p	2245	1606	0.72	−0.48	0.0007848	0.0117644
hsa-miR-150-5p	463	664	1.43	0.52	0.0008064	0.0117644
hsa-miR-136-5p	1534	1135	0.74	−0.43	0.0012255	0.0168841
hsa-let-7i-5p	3802	5118	1.35	0.43	0.0014724	0.0175449
hsa-miR-124-3p	306	205	0.67	−0.58	0.0014857	0.0175449
hsa-miR-362-3p	98	151	1.54	0.62	0.001356	0.0175449
hsa-miR-92b-3p	1060	1529	1.44	0.53	0.0016005	0.0180415
hsa-miR-127-3p	346	239	0.69	−0.54	0.0017286	0.0186383
hsa-miR-329	240	160	0.67	−0.58	0.0019822	0.0196638
hsa-miR-495-3p	1946	1454	0.75	−0.42	0.0019607	0.0196638
hsa-miR-409-5p	46	25	0.53	−0.92	0.0021989	0.0209741
hsa-miR-487a	506	369	0.73	−0.45	0.0026776	0.0245943
hsa-miR-410	647	481	0.74	−0.43	0.0028013	0.0248112
hsa-miR-543	432	316	0.73	−0.45	0.0040771	0.034866
hsa-miR-199a-3p	88	138	1.57	0.65	0.0048687	0.0389497
hsa-miR-199b-3p	88	138	1.57	0.65	0.0048687	0.0389497
hsa-miR-769-5p	47	27	0.57	−0.81	0.0053295	0.0413038
hsa-miR-219-2-3p	2244	1711	0.76	−0.39	0.0060143	0.0443437
hsa-miR-425-5p	147	98	0.67	−0.58	0.0060794	0.0443437
hsa-miR-200a-3p	53	103	1.94	0.96	0.0067664	0.0479449

The name of the mature miRNAs is in accordance with the miRBase V19 nomenclature. Control: normalized counts for the control group; LOAD: normalized counts for the LOAD group; Fold Change and log2 Fold Change correspond to the difference between the LOAD and Control groups; pval: nominal *p*-value determined by the nbinomTest; padj: nominal *p*-value corrected for multiple testing by the BH procedure.

A supervised clustering based on Support Vector Machine (SVM) was built to evaluate whether the 35 differentially expressed miRNAs could be used to classify the hippocampus samples. After training with a subset consisting of 10 LOAD cases and 10 controls, the SVM classifier was tested on the 44 remaining samples including 31 LOAD cases and 13 controls. Among the 44 samples, three (X7H, X27H, X30H) LOAD cases were misclassified as controls whereas all the control samples were correctly assigned, resulting in a sensitivity of 90% and a specificity of 100% for this assay (Fig 1B). The receiver operating characteristic (ROC) curve further showed an area under the curve (AUC) of 0.96 (Fig 1C), confirming the potential application of these 35 miRNAs as markers of LOAD.

### Temporal analysis of miRNAs in the prefrontal cortex of LOAD patients

We next studied 49 prefrontal cortex samples from a second cohort of clinically well-defined patients stratified according to the six classical Braak stages (BRI to VI) and of seven control samples (BR0) (clinical data in Supporting Information Table S2) (Bossers et al, 2010). The global cellular composition in this brain area remains well preserved over different Braak stages in comparison to the hippocampus.

**Figure 2:**
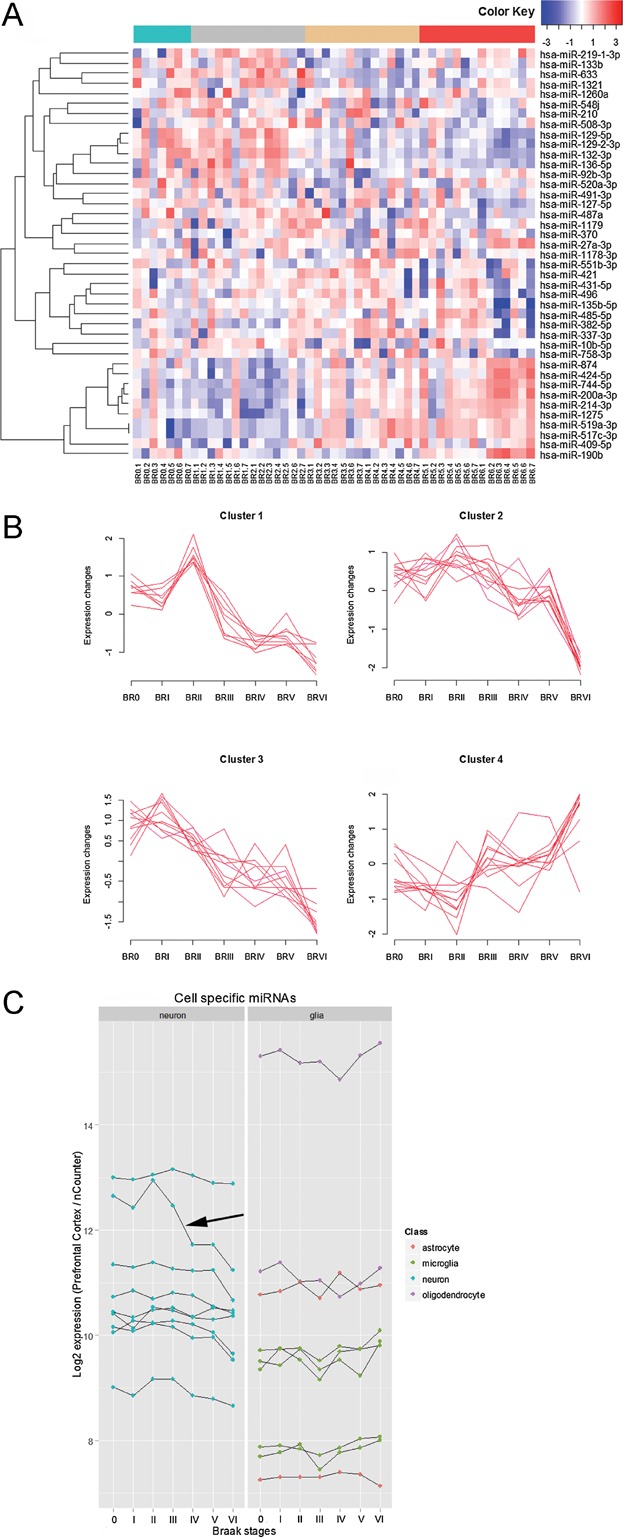
Differential expression of miRNAs in LOAD prefrontal cortex Hierarchical clustering of deregulated miRNAs across the Braak stages. Forty one miRNAs were found to differ between any two Braak stages (*p* < 0.05, nbinomGLMTest after BH adjustment). Hierarchical clustering of miRNAs was obtained using Spearman's correlation distance metric and average linkage. The samples are indicated at the bottom of the heatmap and miRNAs are found at the right side. Red colour in the colour key corresponds to high expression and blue colour to low expression values. The four colours at the top of the heatmap indicate the controls (BR0), early-stages (BRI and BRII), mid-stages (BRIII and BRIV) and late-stages (BRV and BRVI) of disease.Time-course analysis of deregulated miRNAs across the Braak stages. The expression of the 41 deregulated miRNAs was standardized, clustered into four groups and plotted according to the control (BR0) and the six Braak stages. Clusters 1, 2 and 3 correspond to downregulated miRNAs during disease whereas cluster 4 contains the upregulated miRNAs. Downregulation was evident between BRII and III stages for the miRNAs found in cluster 1 whilst a steep decline between BRV and VI characterized the cluster 2. Cluster 3 contains miRNAs gradually downregulated during disease. Cluster 4 was the only group corresponding to some upregulated miRNAs. A biphasic expression profile was observed for clusters 1, 2 and 4 with BRII acting as pivot stage.Expression of cell-type-enriched miRNAs across the Braak stages. The nCounter normalized counts obtained from the prefrontal cortex samples for the neuron-enriched and glia-enriched miRNAs were log2 transformed and plotted against the controls (0) and the six Braak stages (0 to VI). Neuron-enriched miRNAs are shown in blue, microglial-enriched in green, astrocyte-enriched in red and oligodendrocyte-enriched miRNAs in purple. For comparison, the most significantly downregulated miRNA *i.e*. miR-132-3p is indicated by arrow.

Quality assessment showed excellent total RNA integrity for those samples when compared to those in the first cohort studied (Supporting Information Fig S1A). Global expression profile of miRNAs for the 49 prefrontal cortex samples was similar, irrespective of the Braak stages and of the quality of the RNA (Supporting Information Fig S1C). Skewness and overdispersion of the nCounter data were observed (Supporting Information Fig S3A and C). We therefore applied a general linear model (GLM) based on the negative binomial distribution to find differentially expressed miRNAs between any two Braak stages. In total, 41 (6.4%) of 641 tested miRNAs were found to be changed (padj < 0.05, nbinomGLMTest after BH correction) (Table 2 and Fig 2A). The expression analysis of those 41 significantly altered miRNAs showed dynamic expression patterns that were characteristic of downregulated (clusters 1, 2 and 3) and upregulated (cluster 4) miRNAs (Fig 2B). Of importance, some of the miRNA changes were observed at early BRI and BRII stages. For instance, the eight miRNAs in cluster 1, including miR-132-3p, were upregulated between BRI and BRII, and then downregulated between BRII and BRVI. Cluster 2 was mainly characterized by a small loss between BRII and BRIV, followed by a steep decline between late BRV and BRVI stages. Eleven miRNAs such as miR-129-5p, miR-129-2-3p, miR-136-5p, miR-370, miR-409-5p and miR-487a belong to this cluster 2. Cluster 3 consisted of 10 miRNAs showing a gradual downregulation between BR0/BRI and BRVI. Lastly, cluster 4 was made of 12 upregulated miRNAs such as miR-27a-3p, miR-92b-3p and miR-200a-3p. The complete list of miRNAs in each of the four clusters is included in Table 2. In addition, a comparison of miRNAs found to be deregulated between any two Braak stages by the nbinomGLMTest implemented in DESeq to those found by two other popular algorithms, *i.e*. edgeR and Voom + Limma, shows that 40 of 41 miRNAs found to be changed in the prefrontal cortex by DESeq were also called by at least one of the two other methods (Supporting Information Fig S4), therefore supporting the robustness of the analysis.

**Table 2:** Deregulated miRNAs in the prefrontal cortex of LOAD patients

miRNA	BR0	BRI	BRII	BRIII	BRIV	BRV	BRVI	pval	padj	Cluster
hsa-miR-132-3p	6405	5518	7889	5638	3387	3367	2409	7.377E-12	2.279E-09	1
hsa-miR-127-5p	34	27	30	20	15	18	0	2.882E-10	3.98E-08	3
hsa-miR-1321	16	11	25	8	0	2	2	3.864E-10	3.98E-08	1
hsa-miR-210	19	38	22	28	8	5	0	6.376E-08	4.925E-06	3
hsa-miR-214-3p	82	80	24	134	116	144	266	6.686E-07	4.132E-05	4
hsa-miR-496	36	36	41	36	21	31	5	3.796E-06	0.0001955	2
hsa-miR-1178-3p	20	19	16	5	15	7	0	2.605E-05	0.0010853	3
hsa-miR-133b	18	13	21	6	3	7	1	2.968E-05	0.0010853	1
hsa-miR-337-3p	47	52	51	42	54	32	11	3.161E-05	0.0010853	2
hsa-miR-421	40	39	51	40	21	21	10	4.078E-05	0.0012602	1
hsa-miR-548j	26	20	16	15	9	11	1	0.0001314	0.0036922	3
hsa-miR-200a-3p	220	184	156	264	262	299	487	0.0001487	0.0038301	4
hsa-miR-431-5p	30	35	44	25	20	34	7	0.000173	0.0041117	2
hsa-miR-744-5p	187	173	141	246	225	234	326	0.0002172	0.004794	4
hsa-miR-491-3p	23	26	21	11	15	15	2	0.0002616	0.0053892	3
hsa-miR-633	48	42	137	10	3	15	1	0.0003924	0.0075776	1
hsa-miR-485-5p	43	45	41	28	24	38	10	0.0004575	0.0078541	3
hsa-miR-508-3p	17	18	23	14	9	8	1	0.0004413	0.0078541	1
hsa-miR-1275	32	22	5	36	28	26	33	0.0005612	0.0091191	4
hsa-miR-520a-3p	18	20	12	5	5	5	2	0.0006197	0.0091191	3
hsa-miR-758-3p	36	31	28	24	22	14	6	0.0006192	0.0091191	3
hsa-miR-135b-5p	77	80	77	71	60	59	27	0.0006627	0.0093078	2
hsa-miR-1179	23	17	29	15	10	10	2	0.000704	0.0094586	1
hsa-miR-1260a	3320	3175	2392	4873	4274	4708	6518	0.0007804	0.0100482	4
hsa-miR-551b-3p	43	63	41	36	37	25	12	0.0012518	0.0154719	3
hsa-miR-10b-5p	18	19	23	8	6	13	3	0.0013402	0.0159276	1
hsa-miR-129-2-3p	2597	2503	2661	2472	2400	2422	1627	0.0017505	0.0200339	2
hsa-miR-370	144	135	174	135	118	131	74	0.0018428	0.0203364	2
hsa-miR-190b	298	273	241	233	194	286	365	0.0021923	0.0233596	4
hsa-miR-517c-3p	105	110	110	128	180	176	103	0.0026103	0.026019	4
hsa-miR-519a-3p	105	110	110	128	180	176	103	0.0026103	0.026019	4
hsa-miR-129-5p	1140	1080	1199	1147	986	998	737	0.0029627	0.028176	2
hsa-miR-219-1-3p	12	19	14	5	1	4	4	0.0030091	0.028176	3
hsa-miR-409-5p	93	69	92	88	68	71	38	0.0044917	0.0408215	2
hsa-miR-27a-3p	378	326	477	399	432	412	561	0.0046685	0.0412159	4
hsa-miR-424-5p	123	99	87	125	100	119	180	0.0051385	0.0441053	4
hsa-miR-487a	557	506	618	621	458	513	385	0.0053237	0.0444603	2
hsa-miR-382-5p	49	62	51	56	42	56	18	0.0055054	0.0447675	2
hsa-miR-92b-3p	1334	1357	1335	1490	1530	1658	1981	0.0061121	0.048427	4
hsa-miR-136-5p	1061	1241	1203	1242	1184	1060	803	0.0063022	0.0486845	2
hsa-miR-874	218	157	134	244	206	207	263	0.0064902	0.0489137	4

The values represent the mean of the normalized miRNA counts at each Braak stage for the significant miRNAs. pval: nominal *p*-value (nbinomGLMTest); padj: nominal *p*-value corrected for multiple testing using the BH procedure; Cluster: Fuzzy clustering of the significant miRNAs into four classes.

To assess to what extent differences in miRNAs could be explained by changes in the cell types over the disease, we analyzed the prefrontal cortex expression of miRNAs previously shown to be enriched in rodent neurons, astrocytes, oligodendrocytes and microglia, respectively (Jovicic et al, 2013; Lau et al, 2008). Neuron-enriched miRNAs were miR-124-3p, miR-127-3p, miR-128, miR-129-2-3p, miR-129-5p, miR-136-5p, miR-376a-3p and miR-495-3p. Of interest, downregulation of those neuronal miRNA markers was evident between BRV and BRVI stages (Fig 2C), supporting the idea of some neuronal loss at late stages of disease. In contrast, microglial-enriched miR-142-3p, miR-142-5p, miR-146a-5p, miR-150-5p and miR-223-3p were increased at late BRV and BRVI stages (Fig 2C). For oligodendrocytes, we recorded the level of miR-219-2-3p/miR-219-5p and the expression of miR-31-5p and miR-143-3p was monitored for astrocytes. Again, the oligodendrocyte-enriched miRNAs were increased between BRV and BRVI stages but no clear trend was found for the two astrocyte-enriched miRNAs (Fig 2C). More importantly, and in contrast to the late loss of neuronal miRNA markers, downregulation of miR-132-3p was remarkably strong at early and mid-stages of disease already. This difference in miR-132-3p expression was more pronounced than what was found for the other neuronal miRNAs, clearly indicating that downregulation of miR-132-3p cannot be explained by simple cell loss (Fig 2C, indicated by arrow). Overall, it seems likely that miRNAs that are already changing at early and mid Braak stages are linked to the disease process itself rather than being the result of simple alterations in cellular composition of the diseased brain, as this would have been reflected in much more dramatic expression changes of cell-type-enriched miRNAs.

Because our main goal was to reliably identify individual miRNAs deregulated in the disease, we merged the hippocampus and prefrontal cortex data to obtain a ‘high-confidence’ list of miRNAs changed in these two brain areas. Although a vast majority of the 66 unique miRNA alterations appeared to be specific to one of the two brain regions investigated, an overlap of 10 miRNAs including miR-132-3p, miR-129-5p, miR-27a-3p, miR-92b-3p, miR-129-2-3p, miR-136-5p, miR-200a-3p, miR-370, miR-409-5p and miR-487a was found.

### Deregulation of miR-132-3p in the hippocampus, prefrontal cortex and temporal gyrus of LOAD patients

**Figure 3:**
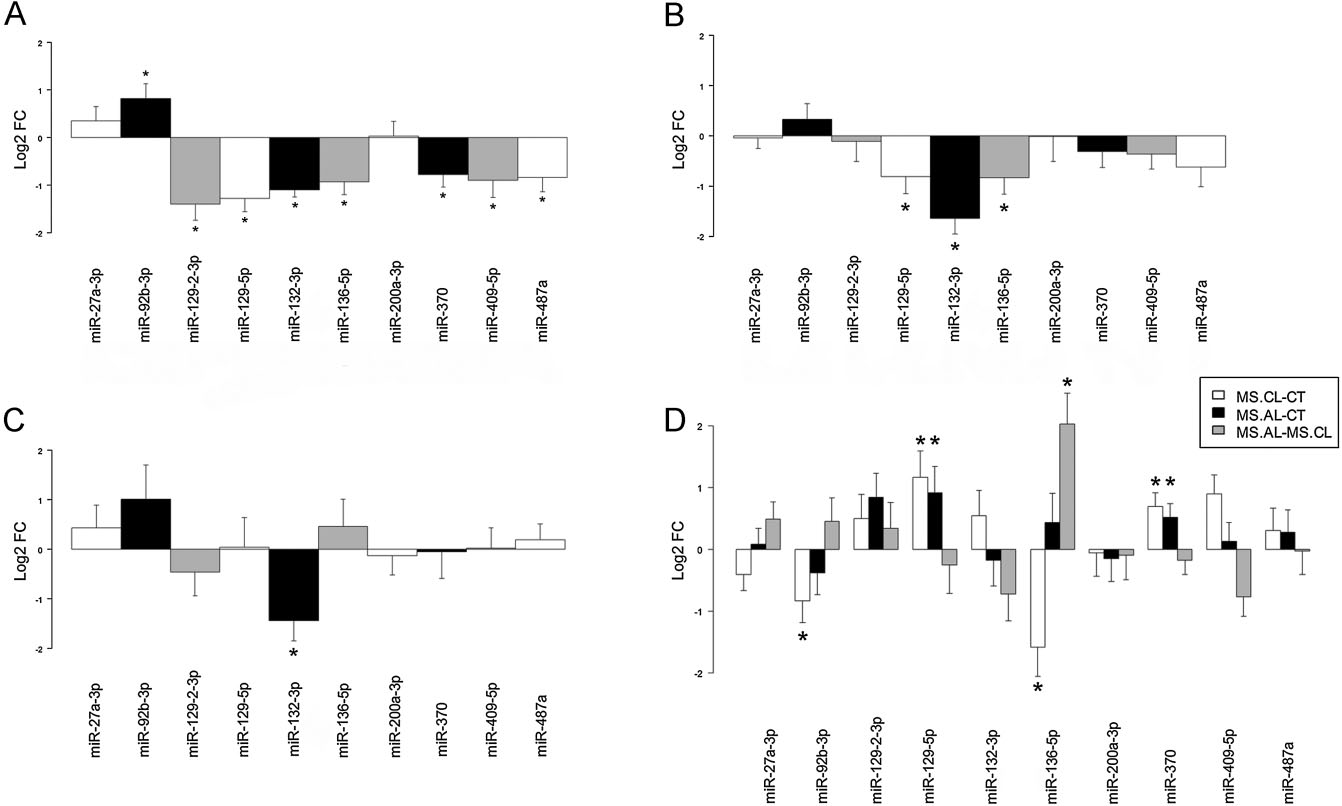
Technical and biological validation of miRNA deregulation in LOAD Validation of the LOAD hippocampus data. Five controls and five LOAD patients were analyzed to validate the 10 miRNAs found to be differentially expressed in both LOAD hippocampus and prefrontal cortex datasets. Fold changes obtained by real-time PCR were log2 transformed (Log2FC) and positive values represent upregulated miRNAs in LOAD whereas negative values correspond to downregulated miRNAs. The miRNAs are indicated at the bottom of the bar plot. Normalization was done with miR-9-5p. * padj < 0.05, ANOVA corrected for multiple testing by the BH procedure. Bars represent standard errors.Validation of the LOAD prefrontal cortex data. Six controls/early-stage and six late-stage samples were selected to confirm expression changes of the 10 miRNAs shown in panel A. The fold changes obtained after real-time PCR between late-stage subjects and controls were log2 transformed. The three downregulated miRNAs (miR-132-3p, miR-129-5p and miR-136-5p) found to be differentially expressed between the two groups are indicated by *. * padj < 0.05, ANOVA corrected for multiple testing by the BH procedure. Normalization was done with miR-9-5p. Bars represent standard errors.Biological validation in the LOAD temporal gyrus. The temporal gyri of eight healthy subjects and eight LOAD patients were analyzed for the expression of the 10 miRNAs found in panels A and B. The real-time PCR fold changes between LOAD and healthy subjects were log2 transformed. * padj < 0.05, ANOVA corrected for multiple testing by the BH procedure. Normalization was achieved using SNORD47. Bars represent standard errors.Deregulation of miRNAs in multiple sclerosis (MS) patients. The active (MS.AL) and chronic (MS.CL) lesions of eight MS patients and the white matter of six controls (CT) were compared by real-time PCR for the expression of the 10 miRNAs initially found to be deregulated in both LOAD hippocampus and prefrontal cortex. Normalization was done with miR-9-5p. * padj < 0.05 (*Post-hoc* Tukey's HSD after ANOVA with p values adjusted for multiple testing by BH correction). Bars represent standard errors.

The 10 miRNAs from the ‘high-confidence’ list were selected for technical validation by real-time PCR on the hippocampus of five LOAD cases (X1H, X14H, X29H, X32H, X9H) and five controls (X49H, X52H, X53H, X71H, X144H). We confirmed the upregulation of miR-92b-3p (padj < 0.05, ANOVA corrected for multiple testing by the BH procedure) and downregulation of miR-129-2-3p, miR-129-5p, miR-132-3p, miR-136-5p, miR-370, miR-409-5p and miR-487a in the five LOAD cases when compared to the control group (Fig 3A). Similarly to the nCounter data, miR-132-3p was the most significantly altered miRNA (padj = 2.88E^−04^). However, we did not confirm changes in the expression of miR-200a-3p (padj = 0.22) nor miR-27a-3p (padj = 0.90) (Fig 3A), therefore eight of 10 selected miRNAs were firmly validated by two assays.

For the prefrontal cortex samples, real-time PCR was performed for the 10 selected miRNAs on a first group consisting of four controls and two early-stages samples (BR0.2, BR0.4, BR0.5, BR0.7, BR1.2, BR1.3) and on a second group of six late-stages brain samples (BR5.7, BR6.2, BR6.3, BR6.4, BR6.5, BR6.7). Similar to the PCR validation of the hippocampus samples, we confirmed miR-132-3p as the most significantly downregulated miRNA in the prefrontal cortex (padj = 1.23E^−03^) (Fig 3B). In addition, miR-129-5p and miR-136-5p were also found to be differentially expressed, resulting in the validation of only three out of 10 miRNAs (Fig 3B). Such difference in the results, pending on sampling and/or techniques used, is an acknowledged concern in the field (Pritchard et al, 2012).

In addition to the hippocampus and prefrontal cortex areas, we aimed at biologically confirming the deregulation of the 10 miRNAs in a third brain region affected in LOAD. Real-time PCR was therefore performed on the temporal gyrus of a third cohort of eight LOAD individuals (five BRV and three BRVI patients) and eight apparently healthy individuals corresponding to non-demented cases (one BR0 and seven BRI subjects) (clinical data in Supporting Information Table S3). There was no difference between the two groups for miR-129-5p, miR-136-5p and seven other miRNAs from the ‘high-confidence’ list (Fig 3C). Obviously those miRNA changes were only seen in specific brain areas or picked by particular techniques, implying the importance of using orthogonal techniques when studying miRNA expression. However, we observed for a third time a significant downregulation of miR-132-3p (padj = 0.02) (Fig 3C). Overall, miR-132-3p was found to be downregulated in three different LOAD brain areas of three cohorts of patients using two different technologies, making this observation highly reproducible and robust.

We determined whether miR-132-3p deregulation in LOAD was the direct result of the inflammatory component of the disease by profiling its expression in another brain disorder characterized by neuroinflammation, *i.e*. multiple sclerosis (MS). Real-time PCR of the white matter of eight control subjects was compared to that of six MS samples with confirmed neuroinflammatory process (active lesions) and matched chronic lesions. We found that miR-132-3p was not differentially expressed between active lesions and controls (*p* = 0.83, *post hoc* Tukey's after ANOVA corrected for multiple testing by the BH procedure) nor between chronic lesions and controls (padj = 0.10) (Fig 3D). Additionally, we found higher levels of miR-129-5p and miR-370 in the active and chronic lesions when compared to the controls (padj < 0.05). More specifically, downregulation of miR-92b-3p and miR-136-5p was only observed between chronic lesions and controls (Fig 3D). Overall, these data show that LOAD and MS share some altered miRNAs, but not miR-132-3p, therefore suggesting that deregulation of this miRNA in LOAD is unlikely a direct consequence of a general inflammatory process.

### Deep-sequencing of miRNAs in the LOAD prefrontal cortex confirms the deregulation of miR-132-3p

**Figure 4:**
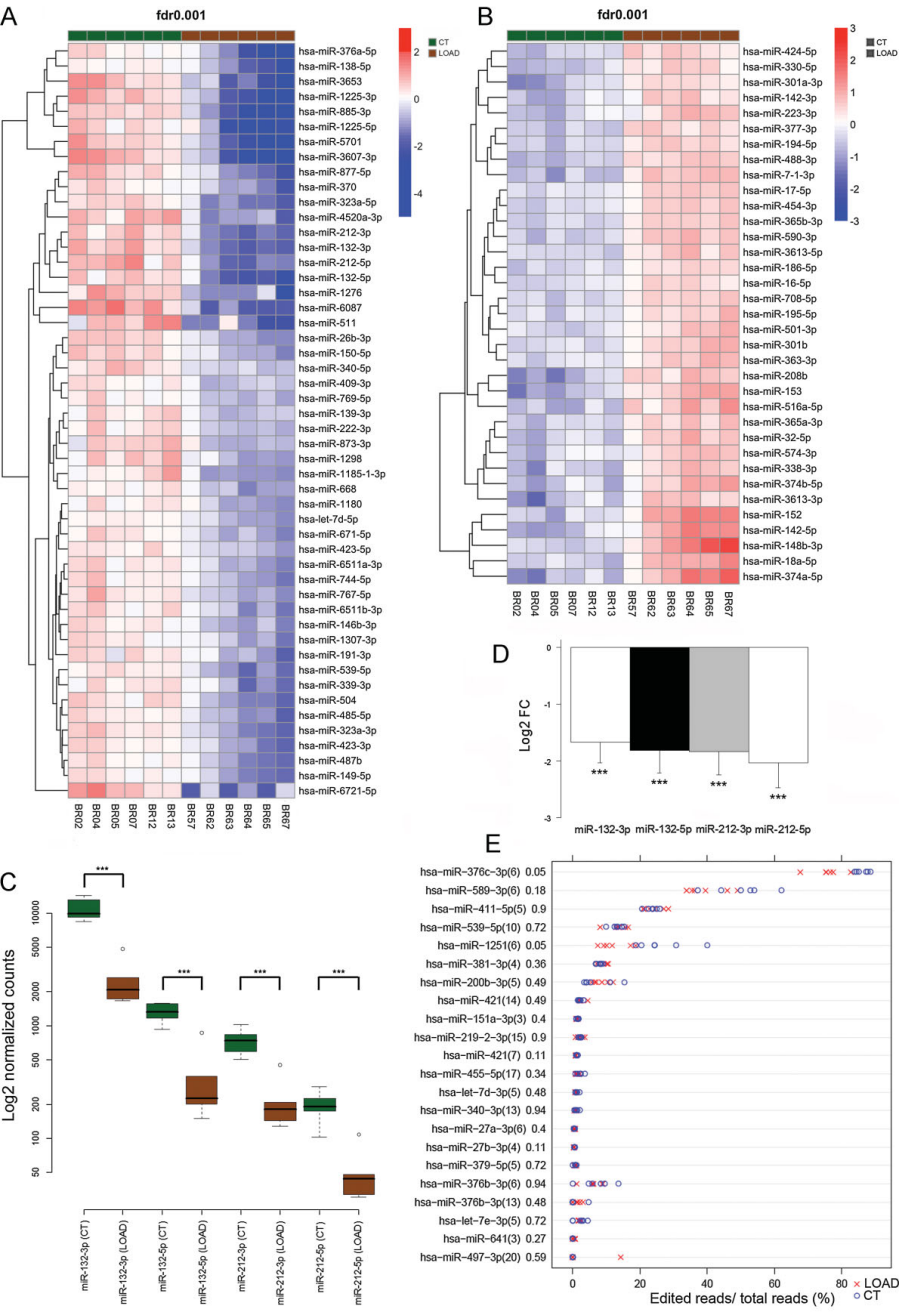
Deep-sequencing of miRNAs in LOAD prefrontal cortex Downregulated miRNAs in the LOAD prefrontal cortex. Prefrontal cortex of 12 subjects was profiled by next-generation sequencing of miRNAs. The six controls/early-stages (CT = BR0.2, BR0.4, BR0.5, BR0.7, BR1.2, BR1.3 samples) and six late-stages (LOAD = BR5.7, BR6.2, BR6.3, BR6.4, BR6.5, BR6.7 samples) were compared and heatmap and cluster dendrogram of the 50 most significantly downregulated miRNAs (padj < 0.001, nbinomTest after BH adjustment) are shown. Green = controls/early-stages (CT) and brown = LOAD patients. Blue: low expression, red: high expression.Upregulated miRNAs in the LOAD prefrontal cortex. Next-generation sequencing of miRNAs of the 12 prefrontal cortex samples shown in panel A revealed 35 upregulated miRNAs (padj < 0.001, nbinomTest after BH adjustment). Hiearchical clustering of upregulated miRNAs shows separation of the LOAD group and controls. Green = controls/early-stages (CT) and brown = LOAD individuals. Blue: low expression, red: high expression.Normalized counts for the four miRNAs of the miR-132/-212 cluster. Normalized reads corresponding to the miRNAs found in the miR-132/-212 cluster and obtained from the 12 sequencing libraries are represented on a logarithm scale. Pairwise comparisons of the two arms (5p and 3p) of each miRNA precursor (pre-miR-132 and pre-miR-212) are shown for the two groups (CT = controls/early-stages represented in green and LOAD = Alzheimer's subjects shown in brown). The expression level of miR-132-3p, miR-132-5p, miR-212-3p and miR-212-5p was, respectively, 4.02, 3.68, 3.22 and 3.61 times lower in the LOAD when compared to the CT group. *** padj < 0.001, nbinomTest after BH adjustment.Validation of the four miRNAs in the miR-132/-212 cluster. Real-time PCR for miR-132-3p, miR-132-5p, miR-212-3p and miR-212-5p was performed on the 12 prefrontal cortex samples used in panels A, B and C. Fold changes between the LOAD group and controls were log2 transformed. The log2 fold changes (Log2FC) obtained for miR-132-3p, miR-132-5p, miR-212-3p and miR-212-5p were −1.67, −1.82, −1.83 and −2.03, respectively. *** padj < 0.001, ANOVA corrected by BH procedure.A-I RNA editing events in the LOAD prefrontal cortex. For each miRNA known to be (A-I) edited, the percentage of editing events at each known position was calculated by dividing the number of reads containing the edited base by the total number of reads corresponding to the miRNA. The six controls/early-stages (CT) libraries are indicated by blue circles and the six LOAD libraries are represented by red crosses. The number in parenthesis indicates the position on the miRNA where the editing event occurs, with the first nucleotide at the 5′ end of the miRNA being the position 1. The last number at the right side of the parenthesis corresponds to the p value adjusted for multiple testing using the BH procedure (padj, Wilcoxon two-sample test).

The nCounter system used in our study turns out to have two major drawbacks. First, it covers only 641 well-expressed human miRNAs but not all the 2038 miRNAs found in the current version of miRBase (V19, August 2012). Moreover, RNA editing can modify the sequence of miRNAs and the nCounter system would have not detected such editing events. To address these two major issues, we expanded our initial profiling experiments by deep-sequencing miRNAs in the 12 prefrontal cortex samples previously used for real-time PCR validation. After lowering the stringency of detection to find miRNAs expressed at very low level (cut-off of 30 counts across the 12 libraries), 839 of 2038 miRNAs were called present with 85 of them differentially expressed between the two groups (padj < 0.001, nbinomTest corrected for multiple testing by the BH procedure) (Table 3). There were 50 and 35 miRNAs found to be, respectively, down and upregulated between LOAD subjects and controls (Fig 4A and B). Among those 85 miRNAs, 22 of them were not present in the nCounter design used for profiling the two large cohorts of patients, therefore representing a set of miRNAs meriting further investigation in the future (Supporting Information Table S4). From this limited list of 22 miRNAs, miR-132-5p and miR-212-5p are found in cluster together with miR-132-3p and miR-212-3p on the chromosome 17 of the human genome, between positions 1,953,200 and 1,953,700 (hg19 build). Of importance, the two miRNAs originating from the pre-miR-132 precursor *i.e*. miR-132-3p and miR-132-5p were both found to be downregulated (padj = 8.42E^−15^ and 1.95E^−10^, respectively). Moreover, miR-212-3p and miR-212-5p were also downregulated (padj = 3.04E^−08^ and 4.74E^−07^, respectively) (Table 3 and Fig 4C), showing that not only miR-132-3p but also the entire miR-132/-212 cluster was altered in the LOAD prefrontal cortex. Real-time PCR was also performed on the 12 prefrontal cortex samples to confirm alteration of the miR-132/-212 cluster, validating the downregulation of the four miRNAs (padj = 0.0005, ANOVA corrected by the BH procedure) (Fig 4D).

**Table 3:** MiRNAs called differentially expressed by next-generation sequencing

miRNA	Control	LOAD	Fold Change	log2 Fold Change	pval	padj
hsa-miR-885-3p	340	75	0.22	−2.19	4.30E-20	3.61E-17
hsa-miR-132-3p	10,811	2513	0.23	−2.10	2.01E-17	8.42E-15
hsa-miR-132-5p	1315	339	0.26	−1.95	6.97E-13	1.95E-10
hsa-miR-3607-3p	3946	576	0.15	−2.78	1.08E-12	2.27E-10
hsa-miR-1225-3p	135	27	0.20	−2.35	1.04E-11	1.74E-09
hsa-miR-153	4341	12,623	2.91	1.54	1.41E-11	1.97E-09
hsa-miR-323a-3p	6852	2640	0.39	−1.38	3.85E-11	4.61E-09
hsa-miR-212-3p	740	215	0.29	−1.78	2.89E-10	3.04E-08
hsa-miR-504	1353	540	0.40	−1.33	4.24E-10	3.95E-08
hsa-miR-7-1-3p	166	432	2.60	1.38	6.46E-10	5.42E-08
hsa-miR-5701	217	46	0.21	−2.25	8.31E-10	6.34E-08
hsa-miR-338-3p	28,741	65,927	2.29	1.20	9.47E-10	6.62E-08
hsa-miR-26b-3p	435	174	0.40	−1.33	2.40E-09	1.55E-07
hsa-miR-877-5p	181	60	0.33	−1.59	3.05E-09	1.83E-07
hsa-miR-3653	226	51	0.23	−2.14	8.27E-09	4.62E-07
hsa-miR-212-5p	197	51	0.26	−1.94	9.04E-09	4.74E-07
hsa-miR-150-5p	4314	1951	0.45	−1.14	1.05E-08	5.20E-07
hsa-miR-488-3p	535	1212	2.27	1.18	1.93E-08	9.02E-07
hsa-miR-152	83	252	3.03	1.60	3.45E-08	1.52E-06
hsa-miR-539-5p	428	185	0.43	−1.21	4.18E-08	1.69E-06
hsa-miR-485-5p	1270	572	0.45	−1.15	4.22E-08	1.69E-06
hsa-miR-301a-3p	3613	7532	2.08	1.06	4.69E-08	1.71E-06
hsa-miR-423-3p	13,338	6057	0.45	−1.14	4.52E-08	1.71E-06
hsa-miR-340-5p	38,252	19,537	0.51	−0.97	6.67E-08	2.33E-06
hsa-miR-323a-5p	66	22	0.33	−1.60	8.14E-08	2.73E-06
hsa-miR-18a-5p	81	215	2.65	1.41	1.60E-07	4.98E-06
hsa-miR-330-5p	1235	2588	2.10	1.07	1.60E-07	4.98E-06
hsa-miR-208b	33	90	2.71	1.44	1.68E-07	5.03E-06
hsa-miR-487b	8194	3884	0.47	−1.08	2.05E-07	5.93E-06
hsa-miR-32-5p	189	417	2.20	1.14	2.56E-07	6.93E-06
hsa-miR-767-5p	563	253	0.45	−1.15	2.53E-07	6.93E-06
hsa-miR-6087	9	2	0.16	−2.61	2.69E-07	7.05E-06
hsa-miR-149-5p	25,531	12,021	0.47	−1.09	4.27E-07	1.09E-05
hsa-miR-376a-5p	3336	1156	0.35	−1.53	5.02E-07	1.24E-05
hsa-miR-142-5p	1367	4387	3.21	1.68	5.28E-07	1.27E-05
hsa-miR-516a-5p	28	76	2.73	1.45	6.19E-07	1.44E-05
hsa-miR-590-3p	125	274	2.19	1.13	9.38E-07	2.13E-05
hsa-miR-138-5p	73,258	30,965	0.42	−1.24	9.82E-07	2.17E-05
hsa-miR-744-5p	8825	4298	0.49	−1.04	1.05E-06	2.26E-05
hsa-miR-377-3p	231	477	2.07	1.05	1.53E-06	3.21E-05
hsa-miR-222-3p	19,064	10,221	0.54	−0.90	1.77E-06	3.62E-05
hsa-let-7d-5p	18,241	9963	0.55	−0.87	2.52E-06	5.03E-05
hsa-miR-301b	398	792	1.99	0.99	2.69E-06	5.25E-05
hsa-miR-374b-5p	570	1429	2.51	1.33	3.31E-06	6.31E-05
hsa-miR-194-5p	320	634	1.98	0.98	4.17E-06	7.72E-05
hsa-miR-370	864	397	0.46	−1.12	4.23E-06	7.72E-05
hsa-miR-1298	137	60	0.44	−1.19	4.67E-06	8.34E-05
hsa-miR-1225-5p	42	12	0.28	−1.83	5.25E-06	9.18E-05
hsa-miR-873-3p	531	244	0.46	−1.12	5.49E-06	9.40E-05
hsa-miR-708-5p	948	1812	1.91	0.93	5.72E-06	9.59E-05
hsa-miR-6511b-3p	296	146	0.49	−1.02	7.77E-06	0.0001
hsa-miR-142-3p	202	401	1.98	0.99	7.63E-06	0.0001
hsa-miR-454-3p	838	1583	1.89	0.92	9.10E-06	0.0001
hsa-miR-374a-5p	244	972	3.98	1.99	9.46E-06	0.0001
hsa-miR-769-5p	72,207	40,795	0.56	−0.82	9.73E-06	0.0001
hsa-miR-1180	1487	788	0.53	−0.92	9.96E-06	0.0001
hsa-miR-6511a-3p	262	130	0.49	−1.01	1.02E-05	0.0002
hsa-miR-363-3p	1570	2898	1.85	0.88	1.13E-05	0.0002
hsa-miR-1276	19	6	0.28	−1.81	1.21E-05	0.0002
hsa-miR-365a-3p	145	290	1.99	0.99	1.23E-05	0.0002
hsa-miR-186-5p	12,389	21,604	1.74	0.80	1.24E-05	0.0002
hsa-miR-365b-3p	195	380	1.95	0.97	1.30E-05	0.0002
hsa-miR-339-3p	2388	1192	0.50	−1.00	1.29E-05	0.0002
hsa-miR-223-3p	324	664	2.05	1.04	1.35E-05	0.0002
hsa-miR-146b-3p	329	168	0.51	−0.97	1.45E-05	0.0002
hsa-miR-195-5p	4050	7216	1.78	0.83	1.59E-05	0.0002
hsa-miR-501-3p	195	377	1.93	0.95	1.69E-05	0.0002
hsa-miR-424-5p	175	339	1.94	0.96	1.82E-05	0.0002
hsa-miR-668	668	358	0.54	−0.90	2.31E-05	0.0003
hsa-miR-3613-5p	143	278	1.95	0.96	2.42E-05	0.0003
hsa-miR-574-3p	739	1356	1.84	0.88	2.46E-05	0.0003
hsa-miR-511	9	2	0.25	−2.00	2.69E-05	0.0003
hsa-miR-6721-5p	8	2	0.24	−2.03	3.12E-05	0.0004
hsa-miR-4520a-3p	15	4	0.29	−1.80	3.53E-05	0.0004
hsa-miR-409-3p	7499	4337	0.58	−0.79	4.07E-05	0.0005
hsa-miR-1185-1-3p	80	32	0.40	−1.31	4.93E-05	0.0005
hsa-miR-1307-3p	1827	1025	0.56	−0.83	5.14E-05	0.0006
hsa-miR-17-5p	672	1202	1.79	0.84	5.47E-05	0.0006
hsa-miR-423-5p	2621	1491	0.57	−0.81	5.54E-05	0.0006
hsa-miR-139-3p	397	216	0.54	−0.88	6.15E-05	0.0006
hsa-miR-671-5p	113	56	0.49	−1.03	6.48E-05	0.0007
hsa-miR-3613-3p	18	44	2.44	1.28	7.18E-05	0.0007
hsa-miR-148b-3p	1211	4542	3.75	1.91	7.91E-05	0.0008
hsa-miR-16-5p	18,412	30,124	1.64	0.71	8.14E-05	0.0008
hsa-miR-191-3p	142	73	0.51	−0.97	8.58E-05	0.0008

Control: normalized counts for the control group; LOAD: normalized counts for the LOAD group; pval: nominal *p*-value determined by the nbinomTest; padj: nominal *p*-value corrected for multiple testing by the BH procedure.

Because RNA editing can affect target recognition by the miRNAs, we surveyed adenosine to inosine (A-I) modification which is the main type of editing event described in the brain (Alon et al, 2012; Chiang et al, 2010). We detected 22 of the 29 known (A-I) RNA editing events in the 12 libraries. Two miRNAs were less edited in LOAD than in controls, on the borderline of significance level (miR-376c-3p and miR-1251 with padj = 0.05, Wilcoxon two-sample test corrected by BH procedure) (Fig 4E). In addition, we characterized miR-132-3p isoforms in depth and found decreased mono-uridylation in LOAD (mean percent of controls = 1.52% of the total reads for miR-132-3p, mean percent of LOAD = 0.71%, *p* = 0.004, Wilcoxon two-sample test), increased miR-132-3p isoform lacking 1 nt at the 3'end (mean percent of controls = 3.76% of the total reads for miR-132-3p, mean percent of LOAD = 8.24%, p = 0.002, Wilcoxon two-sample test) and decreased miR-132-3p isoform lacking 1 nt at the 5'end (mean percent of controls = 2.31% of the total reads for miR-132-3p, mean percent of LOAD = 1.77%, *p* = 0.009, Wilcoxon two-sample test). Overall, these data show that miR-132-3p, in addition to being downregulated, is also differentially sequence-modified in LOAD. Whether such changes in the miR-132-3p sequence are biologically relevant to the disease remains to be determined.

### *In situ* hybridization of miR-132-3p in the human prefrontal cortex

**Figure 5:**
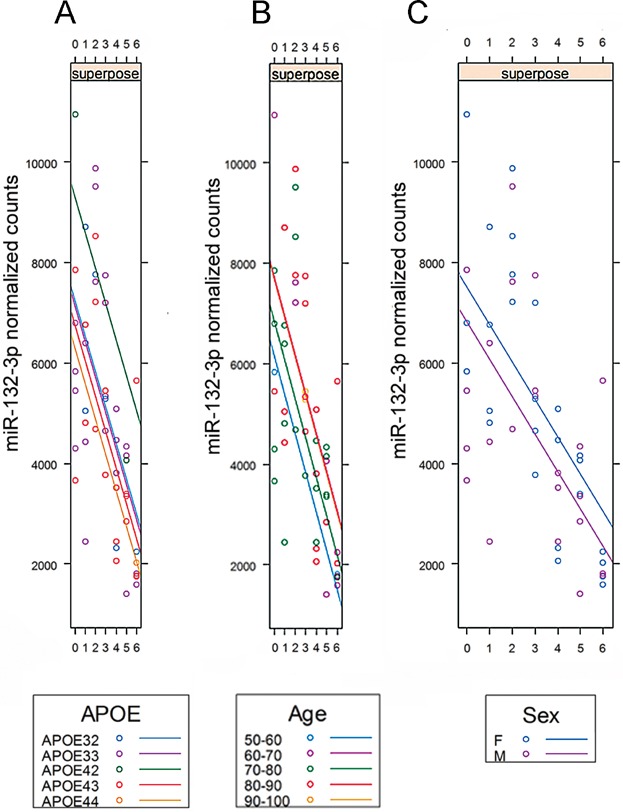
Downregulation of miR-132-3p is specifically related to disease progression Effect of the APOE genotype on miR-132-3p downregulation. The normalized expression level of miR-132-3p in the prefrontal cortex as determined by the nCounter system was plotted against the controls (0) and the six Braak stages (1–6) for which the APOE genotyping was made. The ANCOVA model was used to assess the effect of the different APOE genotypes. For the five genotypes present in the 49 samples *i.e*. APOE 32, 33, 42, 43 and 44, there was no significant difference in the downregulation of miR-132-3p (ANCOVA, *p* = 0.50). Note that APOE42 was found for two patients only.Effect of the age on miR-132-3p downregulation. All the 49 patients were grouped into bins of 10 years, from 50 to 100 years old. For the five different bins, we found no significant difference in the downregulation of miR-132-3p (ANCOVA, *p* = 0.57).Effect of the sex on miR-132-3p downregulation. For the males and the females in the cohort of 49 patients analyzed for the expression of miR-132-3p in the prefrontal cortex, there was no significant difference in the downregulation of miR-132-3p during disease progression (ANCOVA, *p* = 0.20).

Given that miR-132-3p showed a negative correlation to the Braak stages (*r* = −0.63, Pearson's correlation) and to the formation of Tau tangles in the prefrontal cortex (*r* = −0.63, Pearson's correlation), ANCOVA analysis was done to determine whether downregulation of miR-132-3p in LOAD could be explained by additional covariates. The expression of miR-132-3p was related to the Braak stages as expected (*p* = 1.449E^−06^, ANCOVA *F* test) but not to sex (*p* = 0.20) nor to APOE genotype (*p* = 0.50) nor to age (*p* = 0.57) (Fig 5A–C), therefore supporting a specific role for this miRNA in the disease.

**Figure 6:**
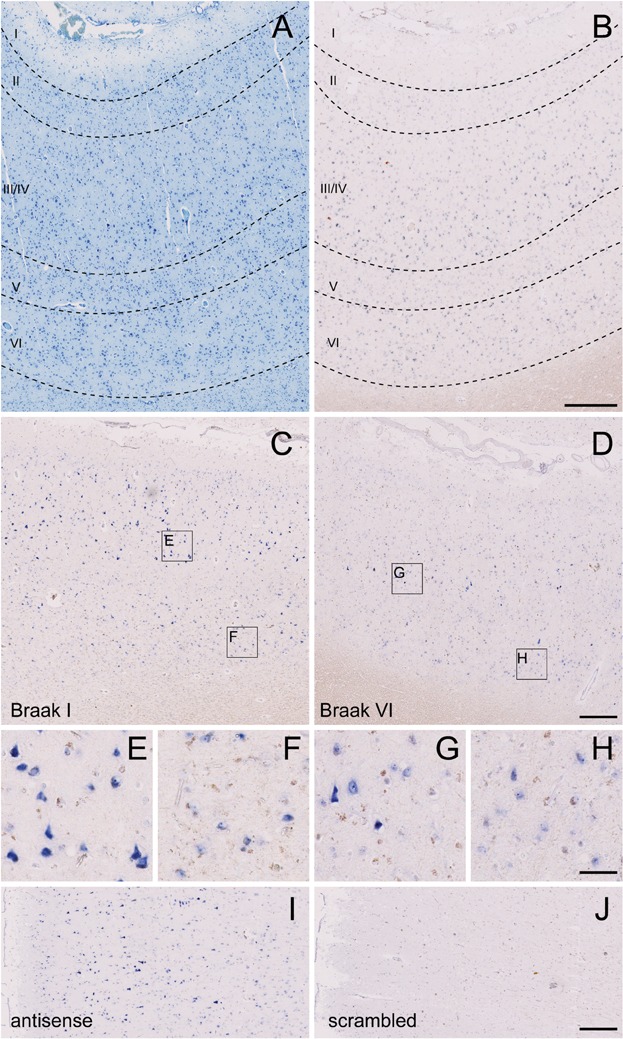
Characterization of miR-132-3p expression in LOAD prefrontal cortex **A-D.** miR-132-3p is expressed in a layer-specific manner. Thionine staining (A) and *in situ* hybridization for miR-132-3p (B) in consecutive prefrontal cortex sections of a LOAD patient (BRVI) reveals that miR-132-3p is predominantly expressed in cortical layers III/IV and VI whereas layer I is devoid of signal. In cortical layers II and V, a low-to-moderate expression of miR-132-3p is seen. The same layer-specific expression pattern is observed in a BRI subject (C and I) and in another LOAD patient (BRVI) (D). Note the overall decrease of the miR-132-3p signal in the LOAD sample.**E-H.** miR-132-3p is constrained to neuronal structures in the prefrontal cortex. Layer III/IV neurons (E, G) express higher levels of miR-132-3p than layer VI neurons (F, H). There is apparent overall decrease in the intensity of the signal in LOAD neurons (G, H) compared to the BRI subject (E, F).**I,J.** Negative control staining with a scrambled probe. To verify the specificity of the miR-132-3p LNA probe, we hybridized consecutive tissue sections of an early-stage patient with the miR-132-3p probe (I) or with a scrambled probe (J). The section hybridized with the scrambled probe was completely devoid of staining whilst intense staining with the miR-132-3p probe was obtained. Scale bars in B, D and J represent 0.25 mm. Scale bar in H represents 50 µm.

We further determined the expression pattern of miR-132-3p in the prefrontal cortex of LOAD patients and controls using *in situ* hybridization. We first investigated whether miR-132-3p was expressed in a particular neuronal population. Virtually all neurons of the prefrontal cortex (94.3 ± 1.9%, *n* = 8 subjects, *n* = 98-219 neurons per subject) (Supporting Information Table S5) were positive for miR-132-3p, suggesting a broad expression in neurons throughout this brain area. Interestingly, the miR-132-3p signal highly varied between different cortical layers (Fig 6A–D). Layer I was negative whilst a low-to-moderate staining intensity was observed in layers II and V. Of note, layers III/IV and VI contained neurons with highest levels of miR-132-3p. In agreement with the nCounter and real-time PCR data, neurons from layers III/IV and VI of a BRVI patient appeared to contain lower levels of miR-132-3p when compared to a BRI subject (Fig 6C and D). Although a more pronounced downregulation of miR-132-3p was found in layers III/IV (Fig 6E–H), the laminar expression pattern was preserved in the BRVI sample (Fig 6C–D), suggesting that deregulation of miR-132-3p was not limited to a specific neuronal population in a defined cortical layer. As negative control, the absence of staining was verified using a scrambled probe (Fig 6I–J).

**Figure 7:**
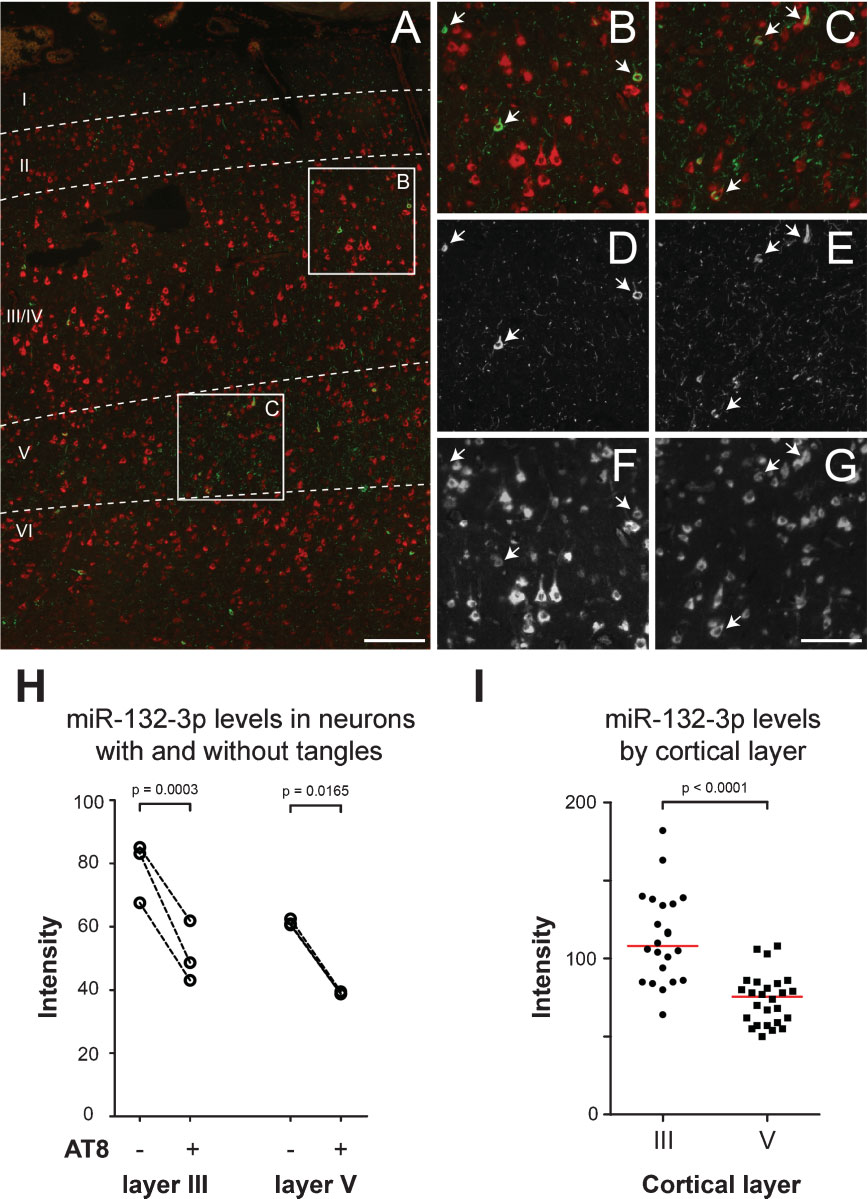
Tangle formation and downregulation of miR-132-3p in LOAD **A-G.** Downregulation of miR-132-3p colocalizes with hyper-phosphorylated Tau. (A) *In situ* hybridization of miR-132-3p (red) and immunohistochemical detection of hyper-phosphorylated Tau (green, AT8 antibody) revealed that hyper-phosphorylated Tau builds up in some miR-132-3p positive neurons of a BRV patient. Panels B, D, F depict tangle formation in layer III neurons (B: merge, D: AT8, F: miR-132-3p). Panels C, E, G depict tangle formation in layer V neurons (C: merge, E: AT8, G: miR-132-3p). Arrows denote examples of miR-132-3p and AT8 colocalization. Scale bar in A corresponds to 100 µm. Scale bar in G represents 50 µm.**H.** miR-132-3p expression is decreased in tangle-bearing neurons. The average fluorescence levels of miR-132-3p in AT8(+) and AT8(−) neurons in layers III and V were quantified for one Braak V individual and two Braak VI subjects. In layer III, the average expression levels are based on 52, 81 and 190 AT8(−) neurons and 18, 20 and 35 AT8(+) neurons, respectively. In layer V, the average expression levels are based on 32, 45 and 145 AT8(−) neurons and 14, 17 and 41 AT8(+) neurons, respectively. Note the significant decrease in miR-132-3p levels in AT8(+) neurons in both cortical layers (*p* = 0.0003 for layer III and *p* = 0.0165 for layer V, paired Student's *t*-test).**I.** Quantification of miR-132-3p expression levels in layers III and V neurons. The graph represents the miR-132-3p fluorescence intensity of all neurons found in panel B (layer III) and panel C (layer V). Note that miR-132-3p expression is significantly lower in layer V neurons of this BRV subject (*p* < 0.0001, Mann–Whitney U test). Red lines indicate median expression levels.

We also investigated a potential link between downregulation of miR-132-3p and presence of hyper-phosphorylated Tau in neuronal cells. In the prefrontal cortex of LOAD patients, Tau tangles are predominantly localized in layers III/IV and V neurons (Hof et al, 1990). By combining *in situ* hybridization and hyper-phosphorylated Tau immunostaining, we found several neurons in the layers III/IV and V of a BRV sample that were positive for both miR-132-3p and hyper-phosphorylated Tau (Fig 7A–G, indicated with arrows). Particularly in layers III/IV, miR-132-3p appeared to be decreased in neurons positive for hyper-phosphorylated Tau (Fig 7B, D and F). However, this expression change was less evident in layer V neurons possibly because of the weaker staining obtained for the neurons of this cortical layer (Fig 7C, E and G). To establish if miR-132-3p was linked to the Tau pathology at the single-neuron level, we quantified miR-132-3p in tangle-positive [AT8(+)] and tangle-negative [AT8(−)] neurons. Of interest, miR-132-3p levels were significantly lower in AT8(+) neurons in both layers III and V, demonstrating a negative correlation between Tau hyper-phosphorylation and miR-132-3p expression on a single-neuron basis (*p* = 0.0003 and 0.0165 for layers III and V respectively, paired Student's *t*-test) (Fig 7H). Because layer V neurons are more prone to develop tangles than layer III neurons, we quantified the expression level of miR-132-3p in those layer V neurons and found it to be significantly lower when compared to layer III neurons (*p* < 0.0001, Mann–Whitney U test) (Fig 7I). Overall, we established a correlation between Tau hyper-phosphorylation and miR-132-3p expression. Thus it appears that downregulation of miR-132-3p goes together with Tau hyper-phosphorylation, a major hallmark of LOAD. The question remains obviously whether there is also a functional relationship between the two.

### miR-132-3p targets FOXO1a, a protein increased in the LOAD hippocampus

The TargetScan algorithm version 6.2 predicts 407 miR-132-3p targets including several transcripts encoding proteins either directly involved in LOAD, *i.e*. MAPT (coding for Tau), or known regulators of Tau at the post-transcriptional level, *i.e*. EP300, SIRT1 and GSK3B. An additional salient feature is the prediction of six members of the Forkhead (FOX) transcription factor (TF) family: FOXA1, FOXN3, FOXO1, FOXO3, FOXP1 and FOXP2. Note that other target prediction tools have confirmed these predictions as well (Supporting Information Table S6). Of interest, when we analyzed published microarray data of prefrontal cortex samples from the same cohort of 49 patients analyzed here (Bossers et al, 2010), we found the enrichment of motifs for the FOX TF family in the promoter region [500 bases upstream the transcription start site (TSS)] of 882 upregulated transcripts (Table 4). We found the FOX motif as significantly enriched (*p*-value < 0.001), particularly as the 2nd strongest enriched motif from a large collection of more than 6000 position weight matrices (Herrmann et al, 2012) (shown in bold in Table 4). As control, we found no enrichment of any FOX motif in the promoters of 673 downregulated genes. Instead, CREB family motifs (CREB1 and ATF3) were enriched in this subset (Supporting Information Table S7). These predictions have been confirmed using another independent method for motif discovery (HOMER), and the FOX motifs remained enriched even within very large upstream sequences of 20 kb (Supporting Information Table S8).

**Table 4:** Enrichment of TF binding sites in the promoter of genes upregulated between BRI and BRVI stages

Rank	Motif id	Z-score	Transcription factor	Targets
1	yetfasco-1638	4.33	NFYC,POLE4	46
**2**	**yetfasco-1467**	**3.89**	**FOXP1,FOXP4,FOXP3,FOXJ1,FOXP2,FOXO1**	69
3	jaspar-MA0012.1	3.78	NA	172
4	jaspar-PB0141.1	3.76	IRF9	87
5	jaspar-PB0116.1	3.76	ELF3	51
**6**	**jaspar-PB0119.1**	**3.74**	**FOXA2,NANOG,NANOGP1**	84
7	transfac_pro-M00091	3.60	NA	44
8	transfac_pro-M01281	3.53	NFATC2,NFATC4	46
9	flyfactorsurvey-br-Z3_FlyReg_FBgn0000210	3.49	NA	34
10	transfac_pro-M00093	3.48	NA	72
11	transfac_pro-M00160	3.46	SRY,SOX5,SOX9,NANOG,SOX7,NANOGP1	72
**12**	**yetfasco-382**	**3.35**	**FOXP1,FOXP4,FOXP3,FOXP2,FOXJ1,FOXF2,FOXO3 FOXO4,FOXO1,FOXJ3,SRF,FOXK1**	31
13	jaspar-PB0186.1	3.30	PAX4,ELF3,TFAP2E	136
**14**	**transfac_pro-M01599**	**3.26**	**FOXP3,FOXF2,FOXP4,FOXP2,FOXJ3**	40
**15**	**transfac_pro-M00477**	**3.23**	**FOXO3,FOXO4,FOXO1,FOXF2,FOXK1,FOXP2,FOXP1FOXP4,FOXJ3**	31
16	jaspar-MA0013.1	3.17	NA	55
17	transfac_pro-M00094	3.15	NA	53
**18**	**transfac_pro-M01137**	**3.13**	**FOXO3,FOXO4,FOXP1,FOXP4,FOXP2,FOXK1**	81
19	transfac_pro-M01010	3.11	HMGA2,HMGA1	52
20	transfac_pro-M00935	3.07	NFATC4,NFATC2,NFATC1,NFATC3	41
**21**	**transfac_pro-M00475**	**3.07**	**FOXO1,FOXO4,FOXO3,FOXF2,FOXP1,FOXP2,FOXP4**	94
			**FOXJ3,FOXK1**	
22	transfac_pro-M00456	3.05	BPTF	140
**23**	**yetfasco-570**	**3.03**	**FOXO4,FOXO1**	47
**24**	**jaspar-MA0317.1**	**3.02**	**FOXO4,FOXO1**	48

There were 882 genes upregulated between BRI and BRVI stages. Motif ids were obtained from the JASPAR, TRANSFAC and YeTFaSCo database. Transcription Factor: TF predicted to bind to the motif; NA: motifs not annotated; Targets: number of upregulated genes containing the motif inside 500 bp upstream the transcription start site.

**Figure 8:**
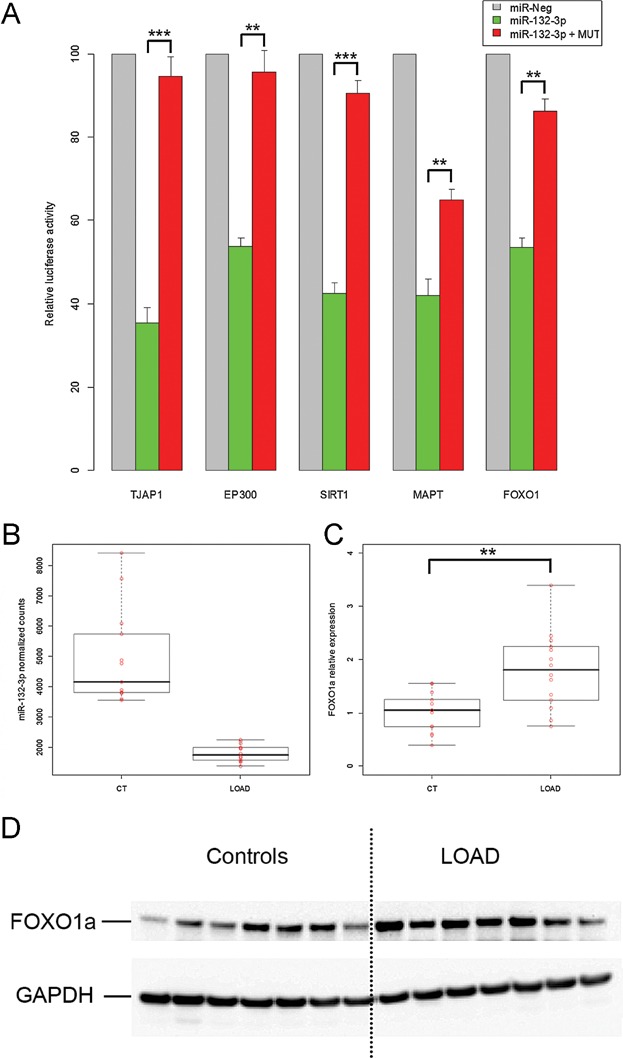
Identification of miR-132 targets of relevance to LOAD *In vitro* validation of miR-132-target pairs. Luciferase assays were performed to determine functional interaction between miR-132-3p and the 3′UTR of predicted targets found in the Tau subnetwork including EP300, SIRT1, MAPT (Tau) and FOXO1. TJAP1 was described as *in vivo* target of miR-132-3p and used as positive control. Reporter plasmids containing the different 3′UTRs were transiently co-transfected with the miRNA negative control 1 (grey bars arbitrary set at 100 for each plasmid) or miR-132-3p (green bars). Red bars indicate data obtained with plasmids containing deletion of the miR-132-3p binding site(s). Data represent mean with SEM of at least three independent transfections. *** *p* < 0.001 and ** *p* < 0.01, ANOVA corrected for multiple testing by BH procedure.Expression of miR-132-3p in 27 selected subjects. Boxplots represent the level of miR-132-3p in the hippocampus of 13 selected controls (CT) and 14 selected LOAD. Normalized counts for miR-132-3p were obtained from the nCounter experiment. There was no overlap between the two groups, with the controls showing higher miR-132-3p expression than the LOAD group. Red circles indicate the different samples.Quantification of FOXO1a expression in the 27 selected subjects. Western-blot analysis of FOXO1a in the hippocampus of 13 controls (CT) and 14 LOAD subjects was done to assess difference in protein expression between the two groups from panel B. Normalization was done with GAPDH. Red circles indicate the 27 different samples. ** *p* < 0.01, Welch two sample *t*-test.FOXO1a expression in the hippocampus of 14 selected subjects. Difference in FOXO1a protein expression is illustrated for seven controls and seven LOAD subjects. Loading control done with GAPDH is shown below FOXO1a. Vertical dashed line separates the two groups.

Of interest, FOXO1a shows a preferential expression in the murine hippocampus (Hoekman et al, 2006). To determine whether miR-132-3p targets FOXO1a, a luciferase construct containing 2.5 kb of the 3′ Untranslated Region (3′UTR) of human FOXO1a (positions 874-3385 relative to the start of the 3′UTR) was co-transfected with miR-132-3p in HEK-293T cells. A 47% decreased of luciferase activity was observed in the presence of miR-132-3p when compared to the co-transfection of an irrelevant miRNA (Fig 8A). A double mutant containing two deletions corresponding to the miR-132-3p binding site predicted by TargetScan (position 2601: 5′-ACUGUUA-3′) and to a non-conserved binding site at position 2270 (5′-ACUGUUA-3′) was less responsive to miR-132-3p (*p* < 0.01, ANOVA corrected by BH procedure) (Fig 8A). In addition, we also tested the entire 3′UTR of EP300, SIRT1 and MAPT. A validated miR-132-3p target, *i.e*. TJAP1 (Cambronne et al, 2012), was included as positive control. Highest decreased luciferase activity was obtained for TJAP1 (65% decreased) followed by MAPT (57%), SIRT1 (57%) and EP300 (46%) (Fig 8A). Luciferase constructs containing deletion of the predicted binding sites were also less responsive to miR-132-3p than wild-type constructs (*p* < 0.001 for TJAP1 and SIRT1, *p* < 0.01 for EP300 and MAPT). Overall, a functional interaction with EP300 and SIRT1 was confirmed and in addition, two other transcripts (MAPT and FOXO1a) encoding for additional proteins of the Tau subnetwork were also found to interact with miR-132-3p, therefore adding evidence that this miRNA could be of relevance to LOAD. We also showed that FOXO1a was indeed increased in LOAD patients with low miR-132-3p level by comparing its expression in the hippocampus of 27 subjects including 13 selected controls and 14 LOAD patients. The controls showed 2.73-fold more miR-132-3p than the LOAD subjects (Fig 8B). Of importance, a 79% increase of FOXO1a was found in the LOAD group (*p* = 0.002, Welch two-sample *t*-test) (Fig 8C and D).

## DISCUSSION

We show that the miR-132/-212 cluster is downregulated in LOAD. In particular, the highest expressed miRNA in this cluster *i.e*. miR-132-3p is downregulated in three different brain areas including the hippocampus, the prefrontal cortex and the temporal gyrus. Downregulation of miR-132-3p was consistently observed irrespective of the three quantitative methods used *i.e*. nCounter system, real-time PCR and deep-sequencing. A pioneer study done with a small cohort of subjects previously proposed miR-132-3p to be deregulated in the LOAD hippocampus and prefrontal cortex (Cogswell et al, 2008) (Supporting Information Fig S5A and B). While this work was under revision, decreased miR-132-3p was also found in the temporal cortex of a cohort of 29 patients and controls (Wong et al, 2013) and of a smaller group of eight AD *versus* eight controls (Hebert et al, 2013), demonstrating the robustness of our observation. Clearly, our work, in addition to these three studies, strongly puts miR-132-3p at the centre stage in the study of miRNAs in LOAD.

A major question of our study was to delineate to what extent miRNA changes accompany disease and disease progression. We attempted to obtain sufficiently large cohorts of LOAD patients and controls to address this question in a statistically sound way. While our work clearly demonstrates that miRNAs are altered in the brain of LOAD subjects, with 35 and 41 miRNAs significantly deregulated in the hippocampus and prefrontal cortex, respectively, we reveal that most of the recorded expression changes are brain area specific, with ten miRNAs deregulated in both regions. We also provide evidence that several of those miRNAs are changed according to the different Braak stages that characterize the progressive severity of the disease (Braak & Braak, 1991).

In the past years, profiling of miRNAs in four different brain regions of LOAD patients was attempted (Cogswell et al, 2008; Hebert et al, 2008; Nunez-Iglesias et al, 2010; Wang et al, 2011). However there is no overlap in the miRNAs identified as altered (Supporting Information Fig 5C). This clear lack of consensus could indeed be due to the small number of samples or the heterogeneity of the brain tissues studied. It should be noted that the well-known bias in the different technologies used for miRNA profiling could be a confounding factor as well (Pritchard et al, 2012). To tackle these caveats, we used a rather large number of patients in our study (*n* = 123 cases compared to 57 for the four previous reports combined together). Additionally, we carefully controlled several inter-individual covariates such as age, sex and *post-mortem* intervals. We also profiled several brain areas using different methodologies to reduce bias introduced by the techniques. We anticipate that, in the future, larger controlled studies with more subjects will be needed to fully capture miRNA changes in the LOAD brain. However, a major limiting factor to overcome is the availability of well-documented frozen brain samples.

Apart from brain area specific expression changes and methodology related considerations, another important question is to what extent recorded miRNA differences reflect disease related processes or more general alterations due to modifications of cellular composition in the affected brain regions. For instance, some of the miRNA changes may not solely be LOAD related but could simply reflect a more neuronal *versus* more glia-like profile. With regard to the hippocampus samples analyzed, we found increases of microglial-enriched miR-142-3p, miR-150-5p and miR-223-3p in the LOAD group. It is also impossible to exclude that loss of neurons contributes to decreased miRNA-132-3p in those samples. However, neuronal miRNAs decreased only (and relatively mildly) at late stages of disease in the prefrontal cortex, suggesting that neurodegeneration can be an explanation for loss of miRNAs at those late stages only. Several miRNAs, such as those found in cluster 1, and including miR-132-3p, emerged as being deregulated already at early Braak stages before the appearance of major neuronal loss. We also found that many of the downregulated genes in LOAD show a CREB motif in their promoter regions and given that miR-132-3p is regulated by CREB (Remenyi et al, 2010; Vo et al, 2005), it is likely that active downregulation of this miRNA is taking place during disease process. The specific deregulation pattern of miR-132-3p also supports the hypothesis that this miRNA plays a rather upstream role in the pathogenic cascade of AD. In agreement, it is of interest to note that miRNA alterations can be observed rather early in the brain of mouse AD models as well. For example, the APPswe/PS1ΔE9 mouse model displays 37 miRNA alterations already at 3 months of age when there is no visible neuronal loss (Wang et al, 2009). We therefore hypothesize that part of the neuronal toxicity caused by Aβ species is mediated, or at least reflected, by changes in the expression of miRNAs. To support this view, hippocampal neurons treated with Aβ42 showed deregulation of 21 miRNAs (Schonrock et al, 2010). It remains to be determined whether miR-132-3p expression is also perturbed in the different mouse models of the disease. Although there was no difference observed for miR-132-3p in the APPswe/PS1ΔE9 mouse (Wang et al, 2009), a more recent study showed this miRNA to be downregulated in the double transgenic APP/PS1 mice at 6 months of age (Wong et al, 2013).

It is also likely that several miRNA changes recorded in our human brain samples are related to some neuroinflammatory changes occurring during disease, for instance reflected in the enrichment of microglial-specific miRNAs at late stages of disease *i.e*. increased miR-142-3p, miR-150-5p and miR-223-3p. Deregulation of miR-132-3p was not however observed in the small group of MS patients analyzed, in agreement with a previous study done with a larger cohort of 30 MS patients/control subjects (Junker et al, 2009). Thus, alteration of miR-132-3p is not systematically observed in a neuroinflammatory context although it has been shown that miR-132-3p mediates anti-inflammatory signalling (Shaked et al, 2009). The targeting of acetylcholinesterase by miR-132-3p may be indeed relevant for the association of the enzyme activity with amyloid load in LOAD (Alkalay et al, 2013; Berson et al, 2008), as well as with the anxiety phenotype and RNA metabolism impairment that are characteristic of the disease (Berson et al, 2012; Shaltiel et al, 2013).

The deregulation of miR-132-3p might be particularly relevant to LOAD at several additional levels. For instance, miR-132-3p has been involved in modulating synaptic activity and plasticity (Wanet et al, 2012). Furthermore, miR-132-3p regulates neuronal outgrowth in response to neurotrophins (Vo et al, 2005), mediates integration of newborn neurons in the adult hippocampus and dentate gyrus (Luikart et al, 2011; Magill et al, 2010), and shapes synaptic structure (Edbauer et al, 2010) and plasticity in the visual cortex (Mellios et al, 2011; Tognini et al, 2011). This brings us to the question which molecular targets of miR-132-3p are relevant to LOAD. We analyzed deregulated transcripts in the LOAD prefrontal cortex samples and identified several miR-132-3p targets including Tau, EP300 and SIRT1, the latter being post-translational regulators of Tau (Min et al, 2010) and FOXO1a. These targets were confirmed using luciferase assays and we further demonstrated upregulation of FOXO1a protein in AD hippocampus samples, in agreement with previous works showing that the FOXO1a transcript is upregulated in function of the severity of the disease (Blalock et al, 2004; Gomez Ravetti et al, 2010; Liang et al, 2012), but see (Bronner et al, 2009). During the revision of the manuscript, a study proposed miR-132-3p to regulate another member of the FOX family of TFs, *i.e*. FOXO3a, which is increased in LOAD and in turn transcriptionally activates pro-apoptotic genes (Wong et al, 2013). Thus, evidence increases that FOXs and the FOXOs in particular, may be regulating important molecular pathways relevant to LOAD. Intriguingly, we found that not only miR-132-3p regulates FOXs but also many of the predicted miR-132-3p targets show in their promoter regions an enrichment of motifs for this family of TFs. We estimate the number of such co-regulation events mediated by miR-132-3p and the FOXs to be up to 311 out of 407 (81.3%) predicted targets (Supporting Information Fig S6).

In conclusion, we provide strong evidence for meaningful changes in miRNA expression during LOAD and downregulation of miR-132-3p as the most salient feature. Recently, alteration of miR-132-3p expression has also been reported in several other neurodegenerative diseases including schizophrenia, progressive supranuclear palsy, Huntington's disease and frontotemporal lobar degeneration with TDP-43 inclusions (FTLD-TDP) (Chen-Plotkin et al, 2012; Johnson et al, 2008; Miller et al, 2012; Smith et al, 2011). Our work thus clearly indicates that miRNAs such as miR-132-3p deserve further functional exploration to deepen our understanding of molecular mechanisms driving not only LOAD but also other neurodegenerative disorders. It is not unlikely that future studies might reveal part of those common molecular pathways that are relevant to these conditions.

## MATERIALS AND METHODS

### Participants and study design

Frozen prefrontal cortex and hippocampus samples were obtained from the Netherlands Brain Bank (Amsterdam, The Netherlands) and the MRC London Brain Bank (London, UK), respectively. The prefrontal cortex collection was made of 49 samples covering the six Braak stages (*n* = 7 samples for each stage) and of seven additional controls. To avoid effect of potential confounding factors, the samples were carefully matched for sex, age, *post-mortem* interval and pH of the cerebrospinal fluid (CSF). The 64 hippocampus samples were made of 23 controls and 41 LOAD cases. All samples were collected according to the legislation and ethical boards of the two Brain Banks. The samples from the temporal lobe of LOAD patients were also obtained from the Netherlands Brain Bank. The MS samples were obtained from the MS Society Tissue Bank (London, UK). The human study was approved by the ethical committees of the University of Leuven and by the UZ Leuven hospital (Reference of the protocol: ML6349).

### Total RNA isolation and nCounter hybridization

Prefrontal cortex samples were snap-frozen in liquid nitrogen and grey matter was dissected in a cryostat. Frozen hippocampus samples were directly dissected on dry-ice. Total RNA was extracted using TRIzol (Invitrogen, Carlsbad, CA, USA) and purified on mirVana columns (Ambion, Austin, TX). The active and chronic lesions of six MS patients and the white matter of normal appearance of eight control subjects were also dissected and snap-frozen in TRIzol. The total RNA quality was assessed on Agilent 2100 Bioanalyzer (Agilent Technologies, Santa Clara, CA, USA). Medium-throughput profiling of miRNAs was performed with the nCounter Human miRNA Expression Assay Kit version 1 (Nanostring Technologies, Seattle, WA, USA). Briefly, 100 ng of total RNA was annealed to the nCounter miRNA Tag reagent, hybridized to the Reporter CodeSet and run on a nCounter Prep Station (VIB microarray facility, Leuven).

### nCounter analysis

All R packages were downloaded from the Bioconductor (http://www.bioconductor.org) and the Comprehensive R Archive Network (CRAN) (http://cran.r-project.org) repositories. The nCounter definition file from the nCounter Human miRNA assay Kit version 1 was re-annotated to match the human miRNAs found in the most recent version of miRBase (V19, August 2012). For the hippocampus samples, the nCounter data were background subtracted using the library NanoStringNorm (version 1.1.14) and a filtering step was applied to keep mean counts >20, followed by testing for differential expression using the nbinomTest (library DESeq, version 1.12.0) and corrected for multiple testing using the BH false discovery rate (FDR) controlling procedure. For the hierarchical clustering of hippocampus miRNAs, data were clustered using Spearman's correlation and average linkage method (library gplots, version 2.11.0.1). The SVM classification of the hippocampus samples was performed using a linear kernel with a gamma = 0.01 and cost = 1 (library e1071, version 1.6-1). The sensitivity or true positive rate (TPR) was defined as TPR = True Positive/(True Positive + False Negative). The specificity or true negative rate (TNR) was calculated as followed: TNR = True Negative/(True Negative + False Positive). The ROC was determined using the library ROCR (version 1.0-5).

The nCounter data obtained from the prefrontal cortex samples were similarly processed except that filtering was set to keep >10 mean counts and differential expression between any two Braak stages was assessed using ANOVADEV (fitNbinomGLMs in the library DESeq). *p*-values were corrected for multiple testing using the BH procedure. Hierarchical clustering of prefrontal cortex miRNAs was performed using Spearman's correlation and average linkage method (library gplots). Fuzzy clustering of significantly deregulated miRNAs (*p* < 0.05, ANOVADEV corrected by the BH procedure) into four clusters was made by using 1.1 degrees of fuzziness (library Mfuzz, version 2.18.0). Additional data analysis was done for the prefrontal cortex dataset using the GLM implemented in edgeR (version 3.2.3) and the Voom transformation found in Limma (version 3.16.5).

### Real-time PCR

To validate miRNA changes, total RNA of five controls and five LOAD hippocampus samples was reverse transcribed using the miRCURY LNA Universal RT kit (Exiqon, Denmark). The reverse transcription mix contained 4 µl of total RNA (200 ng), 4 µl of 5× reaction buffer, 2 µl of enzyme mix and 10 µl of water. The reactions were incubated at 42°C for 1 h followed by inactivation at 95°C for 5 min. The samples were diluted 20× and 4 µl of diluted product was assessed in a PCR reaction mix containing 5 µl of SYBR Green master mix and 1 µl of miRNA specific primers (Exiqon). Real-time PCR was performed on 96-well plates using the LightCycler 480 Real-Time PCR system (Roche, Indianapolis, IN, USA). The PCR conditions were 95°C, 10 min followed by 45 cycles at 95°C, 10 s and 60°C, 1 min. The crossing point (Cp) was determined by using the second derivative method and data were normalized with miR-9-5p. Fold changes and statistical significance were calculated using one-way ANOVA. Validation of the 12 prefrontal cortex samples and analysis of the 14 MS samples were performed similarly using miR-9-5p as normalizer and ANOVA followed by *post-hoc* Tukey's HSD for the comparison of multiple MS groups. Correction for multiple testing was done using the BH procedure. The validation of the temporal lobe of eight LOAD patients and eight controls was done after extracting the RNA using the miRNeasy Mini Kit (Qiagen, Venlo, The Netherlands). The cDNA was prepared using the qScript microRNA cDNA Synthesis Kit (Quanta BioSciences, Gaithersburg, MD, USA) and semi-quantitative PCR was performed using Quanta miR primers and PerfeCta SYBR Green FastMix, Low ROX (Quanta BioSciences). Light emission was measured using the Bio-Rad CFX96TM Real-Time PCR Detection System (Bio-Rad). Normalization was achieved using SNORD47.

### Deep-sequencing of miRNAs

Deep-sequencing of miRNAs from 12 prefrontal cortex samples was performed after reverse transcription of the total RNA and ligation of 5′ and 3′ adaptors using the TruSeq Small RNA Sample Preparation Kit (Illumina, San Diego, CA, USA). After gel purification, the PCR products were sequenced for 35 cycles on an Illumina HiSeq 2000 system (CME Genomics Core, UZ Leuven). The adaptor (5′-TGGAATTCTCGGGTGCCAAGG-3′) was trimmed from the raw sequencing reads using Flicker 3.0 (Illumina) and manual filtering steps were performed to remove low quality reads and reads containing ambiguous nucleotides. Reads longer than 15 nucleotides were mapped to the hg19 build genome using the short-read aligner Bowtie2 (version 2.0.2) and allowing one mismatch. Quantification of mature miRNAs was made using the HTSeq-counts (version 0.5.3p9) taking into account the strand orientation and guided with the mature miRNAs from the miRBase v19 gff3 annotation file. After weighting by one, the low counts were discarded (minimum 30 counts over the 12 samples). Differential expression was done using DESeq (version 1.6.1) after top geometric mean normalization on the 75 top expressed miRNAs (Waggott et al, 2012).

**The paper explained**

PROBLEM:

Alzheimer's disease (AD) is the most common form of dementia. While most of ongoing research is focused on the contribution of Tau and the amyloid β- (Aβ) protein, other molecular pathways are involved in the neurodegenerative process underlying the disorder. Recently, microRNAs have been recognized as important regulators of gene expression and their aberrant expression could lead to perturbation of cellular homeostasis and ultimately to neurodegeneration. However, little is known about the global expression pattern of microRNAs in the brain of patients and the molecular networks potentially regulated by such microRNAs. Moreover, reproducibility of recorded alterations is a major issue.

RESULTS:

In our study, we found deregulation of a small subset of microRNAs in the hippocampus and prefrontal cortex of AD patients. We established miR-132-3p as being the most altered microRNA during disease. Downregulation of miR-132-3p in neuronal cells was inversely correlated with the appearance of the hyper-phosphorylated form of Tau. Additional analysis of miR-132-3p targets points to the FOX transcription factors (TFs) as regulated by and sharing a common subset of targets with this microRNA.

IMPACT:

Our results provide the first large scale analysis of microRNAs altered in AD. When the disease worsens, neuronal cells show lower amount of miR-132-3p and hyper-phosphorylated Tau accumulates in these cells, therefore establishing the downregulation of miR-132-3p as a new marker of AD and potentially other neurodegenerative disorders.

To examine miRNA editing events, we searched for previously reported A-G changes corresponding to A-I editing events. The editing frequency was calculated as the number of reads containing the A-G modification divided by the total number of reads corresponding to the same miRNA. Statistical difference between the two groups was assessed by using the Wilcoxon two-sample test followed by BH correction. The data obtained from the 12 libraries have been submitted to the GEO database under accession number GSE48552.

### Detection of miR-132-3p by *in situ* hybridization

Chimeric 2′-O-methyl/LNA oligoribonucleotide probes were obtained from Ribotask (Odense, Denmark). The sequence of the miR-132-3p LNA probe was:

5′-FAM-lCmGmAlCmCmAlTmGmGlCmUmGlTmAmGlAmCmUlGmUmUlA-3′ with *FAM* denoting a fluorescein tag, *l* a locked nucleic acid (LNA) and *m* a 2′-*O*-methyl modified ribonucleic acid. The sequence of the scrambled probe was 5′-FAM-lAmCmUlTmAmClGmCmUlAmGmUlGmUmGlGmA-mAlCmGmClT-3′. *In situ* hybridization was performed as previously described (Shan et al, 2012). Briefly, 6 µm-thick formalin-fixed, paraffin-embedded sections were de-paraffinized and boiled for 10 min in citrate buffer (pH 6.0). After de-proteinization and de-lipidation, sections were prehybridized for 1 h in hybridization mix [10 mM HEPES (pH 7.5), 5× Denhardt's solution, 50% formamide, 600 mM NaCl and 1 mM EDTA]. Hybridization was performed for 90 min at 58°C with either 25 nM of antisense or scrambled LNA probe diluted in hybridization mix. Sections were subsequently washed for 5 min each in 5×SSC (saline-sodium citrate buffer), 2×SSC, 0.2×SSC (all at 58°C) and 5 min in PBS. For detection of the LNA probe, sections were blocked for 1 h and incubated overnight at 4°C with rabbit anti-fluorescein coupled to alkaline phosphatase (Roche, 1:3000). The sections were developed with NBT/BCIP colour substrate and the colour reaction was stopped in distilled water.

### Co-detection of miR-132-3p and hyper-phosphorylated Tau

Hybridization of the miR-132-3p LNA probe was performed as described above. For immunological detection of miR-132-3p and hyper-phosphorylated Tau, the sections were washed for 2 × 5 min in TBS and incubated with rabbit anti-fluorescein coupled to alkaline phosphatase (1:3000) and mouse anti-human PHF-Tau clone AT8 (Perbio Science, 1:800) in PBS/0.5% BSA for 1 h at RT, followed by overnight incubation at 4°C. Sections were washed 2 × 5 min in TBS and incubated for 1 h with donkey anti-mouse DyLight488 (Bio-Connect, 1:1400). The auto-fluorescence was blocked by incubating 2 × 5 min in TBS, 7 min in 70% ethanol/0.5% Sudan Black, 2 × 5 min in TBS and 2 × 5 min in 0.05 M Tris-HCl (pH 8.2). The colour reaction was performed with Fast Red TR/Napthol AS-MX (Sigma–Aldrich Chemie, The Netherlands) and stopped in distilled water. All sections were coverslipped with Mowiol.

### Quantification of miR-132-3p levels

The images were acquired with an Axioplan microscope at 10× magnification (Carl Zeiss) and analyzed using the Image Pro Plus software (Media Cybernetics, Bethesda, MD, USA). For the quantification of the miR-132-3p fluorescence levels in AT8(+) and AT8(−) neurons, cortical layers III and V were analyzed separately. Briefly, a stretch of layer III or V was manually outlined and fields covering 50% of the outline were randomly selected. Per field, miR-132-3p expression levels were estimated by measuring the average fluorescence intensity in a circle with a diameter of eight pixels (equivalent to 51 µm^2^) placed in the soma of all miR-132-3p positive neurons. Furthermore, each neuron was classified as AT8(+) or AT8(−). Finally, the average miR-132-3p intensity of AT8(+) and AT8(−) neurons was calculated separately for layers III and V. Statistical significance was determined with a paired Student's *t*-test. For the comparison of miR-132-3p levels between cortical layers, the miR-132-3p expression levels were estimated by measuring the average fluorescence intensity in miR-132-3p positive neurons as described above.

### Regulatory analysis

Differentially expressed transcripts between BRI and BRVI stages were determined with Limma. Detection of enriched TFs on protein coding genes was performed on 500 bp promoter sequences of 882 upregulated genes between BRVI and BRI stages (FDR < 0.05) with the parameters for a motif collection of 6683 position weight matrices using an empirical *p*-value (Herrmann et al, 2012). The results were confirmed using Hypergeometric Optimization of Motif EnRichment (HOMER) Suite version 4.2 (Heinz et al, 2010). The program findMotifs.pl was run on a regulatory region of 20 kb centred around the TSS and all the other parameters were used as default to detect the significant motifs from their in-house motif library (1865 motifs).

### Luciferase assays

The 3′UTRs were cloned by PCR using the Phusion enzyme kit (NEB, Ipswich, MA, USA) and human brain cDNA as template (Clontech, Mountain View, CA, USA). For MAPT cloning, PCR was performed using a 201 kb P1-derived artificial chromosome (PAC) containing the human MAPT gene (Poorkaj et al, 2001). The sequences of the PCR primers used for cloning can be found in Supporting Information Table S9. Briefly, PCR products were cut with Asc I and Xho I restriction enzymes and cloned into the psicheck3 vector, a derivative of the psicheck2 plasmid (Promega, Madison, WI, USA) containing an Asc I site inserted into the multiple cloning site (MCS). All plasmids were verified by dideoxy DNA sequencing. For luciferase assays, HEK-293T cells were grown on 24-well plates and transfected with 200 ng of reporter plasmid and 20 pmol of miR-132-3p miRIDIAN miRNA mimic (Dharmacon, Boulder, CO, USA) or miRIDIAN miRNA negative control 1 (Dharmacon) using Lipofectamine 2000 (Invitrogen). After 48 h, cells were processed using the Dual Luciferase Assay kit (Promega) and analyzed using an Envision luminescent plate reader (Perkin-Elmer, Waltham, MA, USA). For each reporter construct, renilla activity was normalized according to that of firefly. Normalized activity after co-transfection of miR-132-3p with a reporter construct was expressed as relative to that obtained after co-transfecting the same plasmid with miRNA negative control 1. Statistical significance was determined using ANOVA with correction for multiple testing by the BH procedure.

### Western-blot

Primary antibody against FOXO1a (C29H4) was from Cell Signalling Technology (Danvers, MA, USA). Primary antibody against GAPDH (5G4) was obtained from Hy-Test (Turku, Finland). Protein extracts from human hippocampus were obtained after cell lysis in RIPA buffer and sonication. After determining the protein concentration, twenty micrograms of proteins were resolved on Bis-Tris gels (Invitrogen) and transferred onto nylon membranes. After blocking with 5% non-fat milk in TBS/Tween-20, blots were incubated overnight with the different primary antibodies. After several consecutive washes in TBS/Tween-20, membranes were incubated with anti-rabbit-HRP conjugated secondary antibody (for FOXO1a) or anti-mouse-HRP secondary antibody (for GAPDH) and developed using the Western Lighting Plus-ECL chemiluminescent reagent (Perkin-Elmer).

## Supplementary material

As a service to our authors and readers, this journal provides supporting information supplied by the authors. Such materials are peer reviewed and may be re-organized for online delivery, but are not copy-edited or typeset. Technical support issues arising from supporting information (other than missing files) should be addressed to the authors.

**Figure S1.** Quality assessment of the total RNA and distribution of the nCounter raw data

**Figure S2.** Comparison of the raw data from hippocampus samples showing different RIN values.

**Figure S3.** Skewness and overdispersion of the nCounter data.

**Figure S4.** Multiple-group comparison of the prefrontal cortex data obtained with the nCounter system.

**Figure S5.** Comparison of deregulated miRNAs in diverse brain areas of LOAD patients.

**Figure S6.** Co-targets of miR-132-3p and the FOX transcription factors.

**Table S1.** Clinical diagnosis of cohort 1 (hippocampus samples).

**Table S2.** Clinical data of sample cases in the cohort 2 (prefrontal cortex samples)

**Table S3.** Clinical diagnosis of sample cases in the cohort 3 (temporal gyrus samples).

**Table S4.** MiRNAs called different by deep-sequencing and without corresponding probes in the nCounter system.

**Table S5.** Percentage of neurons positive for miR-132-3p in the prefrontal cortex.

**Table S6.** miR-132-3p predictions for Tau related targets and FOX targets by different algorithms.

**Table S7.** Enrichment of transcription factor binding sites in the promoter of genes downregulated between BRI and BRVI.

**Table S8.** Detection of FOX motifs by HOMER (known motif approach) in the different gene sets.

**Table S9.** List of PCR primers used for 3'UTR cloning.
