# Supplementary material for: Alteration of the microRNA network during the progression of Alzheimer's disease
Source: EMBO Mol Med. 2013 Sep 9;5(10):1613–34. doi: 10.1002/emmm.201201974 (PMC3799583; doi:10.1002/emmm.201201974)
Supplement: Supplementary file 1 [file emmm0005-1613-SD1.pdf]

EMBO Molecular Medicine

Alterations In The MicroRNA Network During The Progression Of Alzheimer's Disease

Pierre Lau, Koen Bossers, Rekin's Janky, Evgenia Salta, Carlo Sala Frigerio, Shahar Barbash, Roy Rothman, Annerieke S. R. Sierksma, Amantha Thathiah, David Greenberg, Aikaterini S. Papadopoulou, Tilmann Achsel, Torik Ayoubi, Hermona Soreq, Joost Verhaagen, Dick F. Swaab, Stein Aerts and Bart de Strooper

Corresponding author: Bart De Strooper, Katholieke Universiteit Leuven

Review timeline:

Submission date:	03 September 2012
Editorial Decision:	22 October 2012
Revision received:	02 July 2013
Editorial Decision:	24 July 2013
Accepted:	26 July 2013

Transaction Report:

(Note: With the exception of the correction of typographical or spelling errors that could be a source of ambiguity, letters and reports are not edited. The original formatting of letters and referee reports may not be reflected in this compilation.)

Editors: Natascha Bushati, Céline Carret

1st Editorial Decision 22 October 2012

Thank you for the submission of your manuscript "Alterations In The MicroRNA Network During The Progression Of Alzheimer's Disease" to EMBO Molecular Medicine. Please accept my sincere apologies for not replying earlier. We have heard back from the three referees whose comments are below. You will see that the reviewers consider your manuscript to be of potential interest. However, they also raise a number of concerns about the study, which would have to be addressed in a revision of the manuscript.

Importantly, reviewers #1 and #2 highlight that additional data are required to strengthen the link between miR-132 and the Tau pathway. Reviewer #1 also asks for analysis of the association between the ApoE genotype and the observed changes in miRNA expression in the studied samples. In addition, this reviewer would like to see further histochemical characterisation of the sub-groups of neurons expressing miR-132 in the prefrontal cortex and quantitation of the miR-132 in situ data.

Reviewer #2 requires further validation of predicted miR-132 targets. Importantly, the question whether endogenous levels of predicted targets change in the human samples should be addressed. We have asked reviewers #1 and #2 for additional advice with regard to the luciferase assay used for target validation, and it was noted that in Figure 6, panel B, controls as specified in the "additional comments" of reviewer #1 should potentially be included.

Reviewers #2 and #3 note that it remains unclear whether the observed changes in individual miRNAs are specific to Alzheimer's Disease or due to more general inflammatory processes. Reviewer #3 therefore suggests extending the study to additional neurodegenerative or neuroinflammatory diseases. We have consulted reviewers #1 and #2 for their opinion also on this point. While these referees realize that an extension of the entire study will be difficult to carry out,

reviewer #1 suggests histochemically analyzing a limited number of brain sections from patients with other diseases to clarify this issue (see "additional comments" of reviewer #1).

In our view the suggested revisions would render the manuscript more compelling and interesting to a broad readership. We therefore hope that you will be prepared to undertake the recommended experimental revision.

Revised manuscripts should be submitted within three months of a request for revision. If your revision will have to exceed this time frame, please contact the editor. Please also contact the editor as soon as possible if similar work is published elsewhere.

I look forward to seeing a revised form of your manuscript in due course.

***** Reviewer's comments *****

Referee #1 (Remarks):

This manuscript presents an innovative, pathology-associated trans-Atlantic and multi-national collaborative approach for studying the deregulation of miRNAs in the brain of Alzheimer's disease patients. Specifically, the authors searched for overlapping miRNAs that are altered in more than one of the affected regions in the diseased brain. First, they co-profiled miRNAs in the hippocampus of controls and late-onset Alzheimer's disease patients that are presumably sporadic and identified 38 hippocampal miRNAs as deregulated. Then, they found 33 disease-modified miRNAs in the pre-frontal cortex of another cohort, where additionally they could pinpoint those miRNAs whose levels changed in early or late stage Alzheimer's patients grouped by Braak pathology. Zooming into those miRNAs where real-time PCR validated the findings and which were modified in both sites, they focused on miR-132, -129-5p, -136 and -92b and then on miR-132 alone. They found by in situ hybridization and immuno-histochemistry that miR-132 is preferentially expressed in Alzheimer's neurons presenting Tau hyper-phosphorylation; performed some Psicheck viral work to validate suppression by miR-132 of some already known and some additional predicted miR-132 targets that are relevant to the Tau pathway and constructed a computerized network of these targets, based on which they speculate that down-regulation of miR-132 may contribute to disease progression by intercepting those mRNA targets of miR-132 that are involved in the Tau pathway.

Strengths: A significant amount of work has been invested in this study, by two groups of experts in two different fields of expertise. The authors do not shy from admitting some of the major problems in studying miRNAs in post-mortem tissues, explored relatively large groups of tissue samples and made an effort to start their survey in a non-biased manner, also by studying two different brain regions, and proceed with it all the way to a mechanistic explanation. They applied in-depth statistics and combined powerful bioinformatics with some experimental validations; and focused on one miRNA whose neuronal functions have been thoroughly demonstrated by others, which increases the prospects of a significant role for this miRNA in neurodegenerative disease. Additionally, the palliative efficacy-even if marginal-of anticholinesterases was demonstrated in millions of Alzheimer patients, suggesting that at least one target of miR-132, Acetylcholinesterase indeed exists in excess amounts in the diseased brain and thus providing a powerful validation to the message conveyed in this manuscript.

Weaknesses: As is often the case, the same points that strengthen this study also involve obvious weaknesses. The project reads as two separate studies that were stitched together for increasing the impact; the neuropathology and molecular biology tests are not welded well, the choice of Tau as the relevant pathway calls for more tests to be established and some conclusions are not yet proven in an in-depth manner. Part of these difficulties may be addressed by performing additional work, as is detailed below.

1. Introduction: The goal of this study was phrased as " establishing a complete overview of miRNAs in Alzheimer's disease". This over-ambitious statement is presumably based on the assumption that those miRNAs whose expression is changed in the hippocampus and the pre-frontal cortex would be likewise changed is all of the disease-affected neurons; but such a bold assumption requires experimental proof that was not even attempted here. This section should be rephrased to read more cautiously.

2. Technology: Given that the work was performed in more than one site and involved a special technology, the authors should demonstrate that the methods they used worked at the same efficacy and with the same accuracy in both sites and that their validation methods are reliable. For example, Figure 3 B shows a rather weak correlation between the findings reached by the nCounter approach and the qPCR validation tests, with 6 out of 12 miRs showing totally different fold change levels in these two tests. More validation tests are called for to convince the reader.
3. The tested cohorts: Was ApoE profiling done for all of the studied samples? If so, was there an association between the ApoE genotype and the severity of change in the miRNAs discussed here? If not- this genotyping and association analysis can, and should be done.
4. Brain area and cortical layer specificity: The authors argue that the vast majority of the observed changes were specific to the investigated brain area. This calls for testing which sub-groups of neurons were affected, which could significantly increase the importance of their study. Likewise, the differences between cortical layers in the intensity of labeling for miR-132 might reflect neuronal subtype specificity that is amenable for immuno-histochemical validation. This being said, layer V labeling is noted as "weaker" without statistics; such phrases should either include the significance of the difference or be deleted.
5. Discussion: The authors argue that their cohort is the largest one to have ever been tested, but to make such a statement they need to convince the reader that the two tested cohorts in their study may indeed be merged into one, especially given that they explored miRNA profiles in completely different brain regions.
6. The dilemma of cause and effect: The loss of miR-132 in the Alzheimer brain is presented is a causal element in the disease process; however, it might also be the RESULT of this process, especially given the known loss of cortical neurons. The authors should discuss the possibility that miR-132 paucity simply reflects the absence of those neurons expressing it in the healthy brain.
7. Figure 5: This figure presents immuno-staining of TAU and in Situ labeling of miR-132, and the authors argue for causal connection between the two that is not yet proven; the corresponding text should be rephrased to make this point clear.
8. Figure 6 B: This figure shows a luciferase assay of known targets of miR-132 such as SirT1, EP300 and AChE, as well as some new targets such as TJAP1, PSMA2 etc. Indeed, MAPT (tau) was previously suggested to be associated to miR-132, but was never experimentally validated as a miR-132 target. To convincingly prove a functional MAPT/miR-132 association the authors should infect cells with a miR-132 coding virus and demonstrate that this leads to down-regulation of MAPT and the Tau pathway.
9. Neuropathology: Demonstrating that miR-132 is expressed in neurons showing TAU pathology could be strengthened significantly by showing an inverse association between its levels and the extent of TAU pathology in the corresponding neurons.

Additional comments:

1. It is always better, of course to compare at least two diseases, but this would be very difficult to do in the current study. However, the paper includes some immuno-histochemistry with in situ hybridization tests, and those are potentially amenable for experimentation in a limited number of brain sections from patients with other disease(s). That could provide further assurance that the findings are correct while not sending them to repeat a study which already took rather long. If they do not have such sections, they could approach another colleague who does and solve this problem relatively rapidly.

2. I have little trust in these assays on the first place, because they only test binding to an artificial 3'-UTR rather than testing if the target transcript is indeed suppressed via this interaction. But whenever done, these tests MUST include some controls, both with mutated miRNA binding sites and by transfection with irrelevant miRNAs.

Referee #2 (Comments on Novelty/Model System):

1. To use array technology is an OK choice but alternative and probably better technologies are available.
2. The study is unprecedented so far in terms of samples analyzed.
3. Very relevant for biomarker research in LOAD.
4. Human tissue and cell culture to study LOAD. Very Adequate!

Referee #2 (Remarks):

In this study Lau et al., use array technology to measure miRNA levels in the total hippocampus and prefrontal cortex of patients suffering from late onset Alzheimer's disease (LOAD). Patients are stratified according to Braak & Braak stages (B&B). The authors detect a number of miRNAs that correlate with repeat to the expression levels with LOAD. For one miRNA that was downregulated in LOAD (miR132) the authors provide preliminary evidence that miR132 is linked to Tau pathology.

This is a very interesting study. Although the study remains discriptive the analysis of miRNAs in LOAD is a very promising emerging area of research and reliable data is rare so far.

A few questions remain and should be addressed prior to publication.

1. The authors should state which nCounter array they used. I guess it is the Version 1. Then should clearly acknowledge the limitations of this approach. They only measure about half of the known miRNAs and the array technology does not allow measurement absolute levels.

2. Another issue is that especially in early LOAD pathology specific hippocampal subregions are distinctly affected. Thus measuring miRNA in the total hippocampal formation and in cortical regions will most likely miss a number of changes and the authors should address this issue which may explain why miRNAs that has been previously associated with LOAD pathogenesis do not show up in their assay. Are the authors not surprised that they did not find key miRs that have been linked to LOAD previously such as miR29, as shown by the author's themselves but also other miRNAs?

3. The authors should also acknowledge that the data is compromised by the lack of cell type specificity. Especially in the advanced B&B stages this is an issue since the number of neurons is severely decreased while inflammatory processes increase and thus most of the signal likely stems from glia cells. Changes may therefore not only be LOAD related but could simply reflect a more neuronal vs. more glia-like profile.

4. The statistically analysis should be described in greater detail better. The binominal testing is suitable but probably not the best method to detect changes across the different stages of AD.

5. FIG 5: The authors should quantify miR132 levels in pTau positive vs. pTau negative cells of the same individual to strengthen their interpretation.

6. Fig. 6: Where does the array data for the gene-expression used in Fig 6A come from?

7. The authors should analyze via qPCR if mRNA and via IHC or western blot if protein levels change as predicted for the target scan derived candidates. At least some of the key players such as SIRT1, EP3000, RB1, MAPK1, MAPK3 GSK3b should be analyzed.

Additional comments:

With respect to the point 1 raised by reviewer 3 my opinion would be that it is certainly beyond the scope of the study to investigate another neurodegenerative disease in the depth as it has been done by the authors. This relates most of all to the amount of samples analyzed.

However, If I am correct referee 3 raises here as similar point that I have brought up. Especially at later stages of AD there are inflammatory processes that may account for the observed changes since the ratio of neuronal vs. glia cells severely changes.

As for the 2nd comment on the controls of the luciferase assay. This would of course be a nice control that is commonly done in the field of miRNA research. Nevertheless, in the context of this study I would not feel this is really necessary if the authors could demonstrate that mRNA and/or protein levels of - at least some of -the predicted targets change accordingly in the human samples.

Referee #3 (Comments on Novelty/Model System):

This is ceratinly an interesting study in an evolving field. However, authors only compared control vs. AD brains. Thus, it remains unclear whether the observed changes in individual miRNAs are disease specific or rather result from other more general pathogenic processes observed in other brain diseases as well, e.g. inflammation, oxidative stress, neurodegeneration.

Referee #3 (Remarks):

This is an interesting and well performed study describing differences of miRNAs between control and AD post-mortem brains.
Authors used well characterized brain samples from two brain banks and compared miRNA levels of late onset AD brains to age and sex matched controls. miRNAs were profiled with nCounter assay. Bioinformatical processing of the data revealed significant differences in several miRNA in hippocampal and prefrontal cortical areas. In the hippocampus samples, levels of miR-128, -138,-124,-129 were lower, while that of others, including let-7f, -7i, miR-23a,-142-3 and others were increased in AD.
In prefrontal cortical areas mainly other miRNAs were altered, with only little overlap as compared to the hippocampal region.
The alteration of miRNA levels was then validated by RT-PCR. Interestingly some alterations of miRNA levels correlated with Braak stages of tau pathology.
Authors then followed up in more detail miR132 expression by in situ hybridization as it showed a strong negative correlation to Braak stageing. Finally, potential targets of miRNA-132 were found to be connected by network analysis, some also known to be implicated in AD, including tau, gsk3beta and sirt1, and expression validated in hek293 cells.

Overall, this is a very interesting and well controlled study. The strength of the study clearly lies in the relatively high number of brain samples and also inclusion of functional analysis. However, I have a major concern about the specificity of the detected changes for late-onset AD. As brains of other disease conditions have not been included in the study, it remains unclear whether the changes in the miRNA levels were disease specific or rather caused by more general processes in brains undergoing degeneration or chronic inflammation. Indeed, miR-132 for example appears to regulate inflammatory processes.
I would like to encourage the authors to extend the study to at least one additional neurodegenerative/neuroinflammatory disease to assess the specificity of miRNA alterations for late onset AD.

Additional correspondence (author) 26 January 2013

Thank you for your continuing interest in our manuscript. We have worked very hard to answer the criticisms of the referees, but we feel that with regard to validation of candidate targets of the miRNA in brain that more work needs to be done. We have now obtained brain material from the 132 knock out mouse and are performing COFRADIC experiments to replace the synthetic reporter assays we used in the previous publication. We feel that the data from these experiments will be unequivocal with regard to biological relevance. However these are expensive and big studies and we are only halfway as we need a confirmation round. Therefore I would like to ask you whether you can give us 4 additional months to finalize these experiments.

Thank you for your constructive input and patience.

(Editor agreed, 28.01.2013)

1st Revision - authors' response 02 July 2013

I am very pleased to submit the revised version of our manuscript EMM-2012-01974 *"Alteration of The MicroRNA Network During The Progression Of Alzheimer's Disease" by Lau et al.,* for publication in *Embo Molecular Medicine*. We felt very encouraged by the positive feedbacks from both the editorial board and the referees to deepen our study. We have added considerable work to the manuscript and feel that we have now addressed all major comments of the referees. With regard to the specific points that were raised in the previous editorial letter, I included our answer in the next paragraph. For the other criticisms, I added a point by point response to the referees at the end of the letter.
The article is original research not previously published nor submitted elsewhere under consideration. I am consultant for Janssen Pharmaceutica, Belgium but this work has solely been funded by public funding as described in the acknowledgement section. All authors declare no competing financial interests in relation to the work described here.
We added, partially encouraged by the comments of the referees, also several really interesting data to the new manuscript which complement the requests of the referees, and strongly enrich the manuscript.

-Panel C in figure 2 shows the profiles of several cell specific miRNAs which are rather stable over the disease, in contrast to the dramatic change in miR-132 expression. To our knowledge, this is the first demonstration that miRNA alterations recorded in a neurodegenerative disease are not a consequence of changes in cellular content but rather precede them.
-Panel D in figure 3 adds controls of multiple sclerosis brains to determine whether neuroinflammation was the trigger of miR-132 deregulation.
-Figure 4 is an exciting extension of the study using deep-sequencing. This allowed us to expand our analysis of all the >2000 different human miRNAs known to date. Compared to the initial large-scale profiling done with the nCounter, there is little gain with only ~20 additional miRNAs found altered by deep-sequencing and not tested on the nCounter. Moreover, we added in panel 4D significant information on the post-translational editing of miRNAs in Alzheimer's brain, showing that, on the contrary of the current view that such events may be of importance, little difference is indeed found in the disease (we also further detailed in the result section specific modifications of miR-132).
-Fig 5 shows the analyses of clinical covariates in respect to miR-132 expression (as discussed below)
-Fig 7 shows quantitative analyses of miR-132 expression in Alzheimer's brain (panel H and I)
-Figure 8 demonstrates endogenous changes in FOXO1a transcription factor expression. The experiment was performed in 27 human brain samples.

In your previous letter, you pointed out that reviewers #1 and #2 requested additional data to strengthen the link between miR-132 and the Tau pathway.

We have in response added considerable data including bioinformatics analysis and western-blots of patient samples to demonstrate the link. The Tau pathway contains proteins such as MAPT (Tau) and FOXO1a. Because our analysis of transcripts deregulated in LOAD revealed the enrichment of FOX binding sites in their promoter region, we focused on the FOX transcription factors. We detected FOXO1a in the human hippocampus and showed increased FOXO1a in the hippocampus of LOAD patients. To show that miR-132 targets FOXO1a, luciferase assays were performed using the 3'UTR of human FOXO1a and compared to the same region with the miR-132 binding sites removed. We have also provided additional luciferase assays with deletion of the binding sites for miR-132 demonstrating that Tau, Sirt1, and P300 found in the Tau pathway are indeed bona fide targets of miR-132. The additional data are shown in Figure 8.

You requested also more information with regard to the association between the APOE genotype and the observed miRNA changes.

In our paper, we found by using three quantitative methods that miR-132 is down-regulated in Alzheimer's disease. We acknowledged that determining a relationship between miR-132 expression and the clinical covariates was important. We therefore used ANCOVA to assess the relation between miR-132 down-regulation and the clinical covariates for the 49 prefrontal cortex samples.

The data are shown in Figures 5A, 5B and 5C. We did not find significant differences when miR-132 expression was corrected for the APOE genotype, age or sex.

Histochemical characterization of the sub-groups of neurons expressing miR-132 in the prefrontal cortex was requested.

We agreed with the importance of localizing miR-132 in neurons and analyzed its expression in 8 subjects using in situ hybridization. We observed that ~94% of the counted prefrontal cortex neurons (from 98 to 219 neurons per subject) were positive for miR-132, suggesting a broad expression. The data are shown in Supplementary Table 5.

Reviewer #2 required further validation of predicted miR-132 targets. Importantly, the question whether endogenous levels of predicted targets change in the human samples should be addressed. We have asked reviewers #1 and #2 for additional advice with regard to the luciferase assay used for target validation, and it was noted that in Figure 6, panel B, controls as specified in the "additional comments" of reviewer #1 should potentially be included.

Because of the importance of FOXO1 in regulating expression of genes deregulated in LOAD, we performed western-blot on hippocampus samples of LOAD patients and found increased expression of the endogenous protein.
For the luciferase assays, we deleted the predicted miRNA binding sites from the 3'UTRs tested in Figure 8 and also transfected irrelevant miRNA as control (set at value=100).

Reviewers #2 and #3 note that it remains unclear whether the observed changes in individual miRNAs are specific to Alzheimer's Disease or due to more general inflammatory processes. Reviewer #3 therefore suggests extending the study to additional neurodegenerative or neuroinflammatory diseases. We have consulted reviewers #1 and #2 for their opinion also on this point. While these referees realize that an extension of the entire study will be difficult to carry out, reviewer #1 suggests histochemically analyzing a limited number of brain sections from patients with other diseases to clarify this issue (see "additional comments" of reviewer #1).

We agreed with the importance of determining whether the down-regulation of miR-132 results directly from an inflammatory process. We have in addition tried to analyze to what extent the changes observed were a consequence of general cellular alterations or specific for an underlying disease process.
We first extended the study to an unrelated neuroinflammatory disorder i.e multiple sclerosis and profiled miR-132 in the active lesions of 8 multiple sclerosis patients and did not find any statistical difference when compared to 8 subjects having a normal white matter. We also did not find difference in miR-132 expression when comparing the chronic lesions of these patients to the controls.
The data are incorporated in Figure 3D and discussion about disease specificity was included in the discussion section. We state that miR-132 was not found changed in multiple sclerosis but in schizophrenia, progressive supranuclear palsy, Huntington's disease and frontotemporal lobar degeneration with TDP-43 inclusions (FTLD-TDP). We believe that this alteration of miR-132 is a very important clue towards a process unifying different neurodegenerative disorders.
We also added in addition new data in fig 2C which are crucial in this regard. Most cell specific miRNAs do not show major alterations over the different Braak stages of AD, but miR-132 shows first a small increase and then a really dramatic and specific decrease, which cannot be explained by simple cell loss as this experiment demonstrates.

While our work was under revision, two papers came out that might compete with our work to a certain extent. The first paper (Hebert et al, PMID: 23403535) was a study with 8 patients and 8 controls, and found by qpCR alteration of miR-132 in the Alzheimer's patients. Our work contains 123 human samples, localizes miR-132 expression in neurons, shows using time-series analysis that this deregulation is not due to simple loss of neurons but rather actively contributes to disease progression. The second paper by Wong et al. (PMID: 23585551) was a much more detailed analysis of the potential involvement of miR-132 in Alzheimer's Disease. They provide considerable evidence that this miRNA regulates a member of the Foxo family, i.e FOXO3 and link the dysregulation of this transcription factor to an apoptotic pathway centered around BIM1. This work is very exciting and interesting and reinforces part of the conclusions of our manuscript.

However there is considerable difference in the depth of analysis of the clinical material between ours and this other manuscript. Again, we have 123 against 29 samples used in that other report. We found by western-blot up-regulation of FOXO1 in the hippocampus whereas FOXO3 was found to be up in the prefrontal cortex. Additionally, we extend this study by showing that promoters of half of the genes coding for transcripts predicted as miR-132 targets contain FOX motifs. This exciting observation will facilitate discovery and validation of targets of relevance to the disease. In addition, we have focused on establishing to what extent miRNA alterations in different brain areas are correlated with Alzheimer's disease. A particular strength is the Braak series of patients that we studied and which allowed us to show the stage-specific alteration of miRNAs. We provided also deep-sequencing data to be as comprehensive as possible concerning all the ~2000 known miRNAs. However, current costs associated with deep-sequencing experiments preclude the routine analysis of hundreds of samples for the time being, thus making our study a valuable resource. The in situ hybridization data provided by us in figures 6 and 7 are also very unique and will stimulate the field to explore cell specific changes of miRNAs in the brain.

We therefore see those two publications as complementary to and not as competitive with our study. We believe moreover that our study, by its broad approach and the depth of analysis, will serve as a landmark paper in the study of miRNA alterations in the brain of AD patients and will stimulate a cascade of follow-up work, including single-cell analysis of miRNA changes in neurons or microglia of patients (for instance, our data correlating miR-132 alteration with abnormally expression of AT8 phosphorylated Tau in neurons is certainly a good motivation to go into that direction).

In conclusion, we provide now a large battery of additional works providing a rather comprehensive picture of miRNA changes in Alzheimer's brain and puts miR-132 as key miRNA in the disease process. We believe that our work will serve as a reference for the field, and will stimulate the field to provide more systematic attention to this intriguing class of molecules. This will lead to in depth insight in the pathogenic processes that underlay the disorder.

On behalf of all co-authors, I want to thank you for your interest in our work and the constructive reviewing which has allowed us to considerably improve our work.

Point-by-point response to the reviewers
Reviewer 1 comments:

This manuscript presents an innovative, pathology-associated trans-Atlantic and multi-national collaborative approach for studying the deregulation of miRNAs in the brain of Alzheimer's disease patients. Specifically, the authors searched for overlapping miRNAs that are altered in more than one of the affected regions in the diseased brain. First, they co-profiled miRNAs in the hippocampus of controls and late-onset Alzheimer's disease patients that are presumably sporadic and identified 38 hippocampal miRNAs as deregulated. Then, they found 33 disease-modified miRNAs in the pre-frontal cortex of another cohort, where additionally they could pinpoint those miRNAs whose levels changed in early or late stage Alzheimer's patients grouped by Braak pathology. Zooming into those miRNAs where real-time PCR validated the findings and which were modified in both sites, they focused on miR-132, -129-5p, -136 and -92b and then on miR-132 alone. They found by in situ hybridization and immuno-histochemistry that miR-132 is preferentially expressed in Alzheimer's neurons presenting Tau hyper-phosphorylation; performed some Psicheck viral work to validate suppression by miR-132 of some already known and some additional predicted miR-132 targets that are relevant to the Tau pathway and constructed a computerized network of these targets, based on which they speculate that down-regulation of miR-132 may contribute to disease progression by intercepting those mRNA targets of miR-132 that are involved in the Tau pathway.

Strengths: A significant amount of work has been invested in this study, by two groups of experts in two different fields of expertise. The authors do not shy from admitting some of the major problems in studying miRNAs in post-mortem tissues, explored relatively large groups of tissue samples and made an effort to start their survey in a non-biased manner, also by studying two different brain regions, and proceed with it all the way to a mechanistic explanation. They applied in-depth statistics and combined powerful bioinformatics with some experimental validations; and focused on one miRNA whose neuronal functions have been thoroughly demonstrated by others, which increases the prospects of a significant role for this miRNA in neurodegenerative disease. Additionally, the palliative efficacy-even if marginal-of anticholinesterases was demonstrated in millions of Alzheimer patients, suggesting that at least one target of miR-132, Acetylcholinesterase

indeed exists in excess amounts in the diseased brain and thus providing a powerful validation to the message conveyed in this manuscript.

We thank the referee for constructive and encouraging comments

Weaknesses: As is often the case, the same points that strengthen this study also involve obvious weaknesses. The project reads as two separate studies that were stitched together for increasing the impact; the neuropathology and molecular biology tests are not welded well, the choice of Tau as the relevant pathway calls for more tests to be established and some conclusions are not yet proven in an in-depth manner. Part of these difficulties may be addressed by performing additional work, as is detailed below.

We appreciate constructive criticism and have worked along the lines indicated in the specific criticisms below.

1. Introduction: The goal of this study was phrased as " establishing a complete overview of miRNAs in Alzheimer's disease". This over-ambitious statement is presumably based on the assumption that those miRNAs whose expression is changed in the hippocampus and the pre-frontal cortex would be likewise changed is all of the disease-affected neurons; but such a bold assumption requires experimental proof that was not even attempted here. This section should be rephrased to read more cautiously.

The goal of the study was rephrased more cautiously as "We aim here to determine miRNA alterations in LOAD by profiling two large and independent cohorts of patients".

2. Technology: Given that the work was performed in more than one site and involved a special technology, the authors should demonstrate that the methods they used worked at the same efficacy and with the same accuracy in both sites and that their validation methods are reliable. For example, Figure 3 B shows a rather weak correlation between the findings reached by the nCounter approach and the qPCR validation tests, with 6 out of 12 miRs showing totally different fold change levels in these two tests. More validation tests are called for to convince the reader.

- All analysis were performed in Leuven, apart for the new series that were added to this version of the manuscript which were samples from temporal cortex, using an independent third series of AD brain samples. We are therefore pretty sure that the differences in results are the consequence of technology. As we discuss in the manuscript divergences between different technologies to detect miRNAs are not unusual and we therefore believe that one of the strengths of our work is the orthogonal series of experimental approaches that we have used to analyze the micro-RNA-ome in Alzheimer.
-We have added a considerable amount of additional work and validation, including deep sequencing. We show the down regulation of miR-132 in three different brain areas, using four different detection methods (nCounter, qPCR, deep-sequencing, in situ hybridization). We are not aware of any other paper showing deregulation of a miRNA in the disease using the levels of technical/biological validation shown in our paper.

3. The tested cohorts: Was ApoE profiling done for all of the studied samples? If so, was there an association between the ApoE genotype and the severity of change in the miRNAs discussed here? If not- this genotyping and association analysis can, and should be done.

We extracted the APOE information from the 49 prefrontal cortex samples and showed that APOE genotype did not influence how miR-132 was affected. Data are shown in Figure 5A. We did not investigate APOE for the hippocampus samples because of the lack of association between APOE and miR-132 expression in the prefrontal cortex.

4. Brain area and cortical layer specificity: The authors argue that the vast majority of the observed changes were specific to the investigated brain area. This calls for testing which sub-groups of neurons were affected, which could significantly increase the importance of their study. Likewise, the differences between cortical layers in the intensity of labeling for miR-132 might reflect neuronal subtype specificity that is amenable for immuno-histochemical validation.

We found that virtually all neurons were positive for miR-132. This suggests broad expression in neurons throughout the prefrontal cortex rather than expression in a subclass of neurons. Data are shown in Supplementary Table 5.

This being said, layer V labeling is noted as "weaker" without statistics; such phrases should either include the significance of the difference or be deleted.

Figure 7I contains now the statistics ($p<0.0001$, Mann-Whitney U test).

5. Discussion: The authors argue that their cohort is the largest one to have ever been tested, but to make such a statement they need to convince the reader that the two tested cohorts in their study may indeed be merged into one, especially given that they explored miRNA profiles in completely different brain regions.

We profiled a cohort of 49 prefrontal cortical and a second cohort of 64 hippocampampal material from different subjects. These two independent cohorts without any merging contain already each more patients than any single other studies that we are aware of. We also feel that our study provides strong encouragement to try to set up larger consortia now to increase the number of analyzed brains, and to explore in depth the pathways affected in different stages of the disease. This should provide new biomarkers and might yield important novel insights into disease pathways. However, as is also clear form our manuscript, it is critical to have sufficient patient material and to stratify according to disease progress if meaningful data have to be extracted.

6. The dilemma of cause and effect: The loss of miR-132 in the Alzheimer brain is presented is a causal element in the disease process; however, it might also be the RESULT of this process, especially given the known loss of cortical neurons. The authors should discuss the possibility that miR-132 paucity simply reflects the absence of those neurons expressing it in the healthy brain.

Experimentally we include panel 2C, showing the profiles from cell specific microRNA over different stages of the disorder. This figure dramatically demonstrates the specificity of the miR-132 change. In the Discussion, we included a very long paragraph discussing the important issue raised by this referee. It starts with "Apart from brain area specific expression changes and methodology related considerations, another important question is to what extent recorded miRNA differences reflect disease related processes….

7. Figure 5: This figure presents immuno-staining of TAU and in Situ labeling of miR-132, and the authors argue for causal connection between the two that is not yet proven; the corresponding text should be rephrased to make this point clear.

We agree with the referee and have attenuated our conclusions. Text includes: "Overall, we established a correlation between Tau hyper-phosphorylation and miR-132 down-regulation. Thus it appears that deregulation of miR-132-3p goes together with Tau hyper-phosphorylation, a major hallmark of LOAD. The question remains obviously whether there is also a functional relationship between the two."

8. Figure 6 B: This figure shows a luciferase assay of known targets of miR-132 such as SirT1, EP300 and AChE, as well as some new targets such as TJAP1, PSMA2 etc. Indeed, MAPT (tau) was previously suggested to be associated to miR-132, but was never experimentally validated as a miR-132 target. To convincingly prove a functional MAPT/miR-132 association the authors should infect cells with a miR-132 coding virus and demonstrate that this leads to down-regulation of MAPT and the Tau pathway.

We have performed extensive work to make this link harder, but failed to obtain direct evidence in cell culture for this regulatory mechanism. See however remark 9 that strengthens the correlation though. The only additional functional evidence is that mutating the binding site of miR-132 in the 3'-UTR of Tau affects the down regulation of the Tau-luciferase construct by miR-132. In our revised paper, we acknowledge the lack of data supporting the direct in vivo interaction of MAPT/miR-132 and minimally discussed therefore this possibility. We refocus the discussion around proteins possibly affecting Tau and on Foxo transcription factors.

9. Neuropathology: Demonstrating that miR-132 is expressed in neurons showing TAU pathology could be strengthened significantly by showing an inverse association between its levels and the extent of TAU pathology in the corresponding neurons.

Quantification showing inverse correlation between phosphorylated Tau and miR-132 is included in Figure 7H.

Additional comments:

1. It is always better, of course to compare at least two diseases, but this would be very difficult to do in the current study. However, the paper includes some immuno-histochemistry with in situ hybridization tests, and those are potentially amenable for experimentation in a limited number of brain sections from patients with other disease(s). That could provide further assurance that the findings are correct while not sending them to repeat a study which already took rather long. If they do not have such sections, they could approach another colleague who does and solve this problem relatively rapidly.

We believe that we have addressed largely this point of discussion between the different refs by including additional analysis on a series of MS patient samples, the analysis of cell specific changes (2C) and by improving our in situ analysis as discussed above.

2. I have little trust in these assays on the first place, because they only test binding to an artificial 3'-UTR rather than testing if the target transcript is indeed suppressed via this interaction. But whenever done, these tests MUST include some controls, both with mutated miRNA binding sites and by transfection with irrelevant miRNAs.

We have included these controls as mentioned already above

Reviewer 2 comments:

Referee #2 (Comments on Novelty/Model System):

1. To use array technology is an OK choice but alternative and probably better technologies are available.
2. The study is unprecedented so far in terms of samples analyzed.
3. Very relevant for biomarker research in LOAD.
4. Human tissue and cell culture to study LOAD. Very Adequate!

Referee #2 (Remarks):

In this study Lau et al., use array technology to measure miRNA levels in the total hippocampus and prefrontal cortex of patients suffering from late onset Alzheimer's disease (LOAD). Patients are stratified according to Braak & Braak stages (B&B). The authors detect a number of miRNAs that correlate with repeat to the expression levels with LOAD. For one miRNA that was downregulated in LOAD (miR132) the authors provide preliminary evidence that miR132 is linked to Tau pathology.

This is a very interesting study. Although the study remains discriptive the analysis of miRNAs in LOAD is a very promising emerging area of research and reliable data is rare so far.

We feel very encouraged by these comments.

A few questions remain and should be addressed prior to publication.

1. The authors should state which nCounter array they used. I guess it is the Version 1. Then should clearly acknowledge the limitations of this approach. They only measure about half of the known miRNAs and the array technology does not allow measurement absolute levels..

The array version 1 was used and it is now clearly stated in Material and Methods section.
"Medium-throughput profiling of miRNAs was performed with the nCounter Human miRNA Expression Assay Kit version 1"
In addition, we clearly acknowledged that it does not measure all the miRNAs.

To profile all known human miRNAs, deep-sequencing was performed on 12 prefrontal cortex samples. We show that only 22 miRNAs found differentially expressed by deep-sequencing and not originally present in the nCounter merits further investigation (Supplementary table 4). We add those data to the new version of the manuscript, including an initial analysis of editing of miRNAs in AD.

2. Another issue is that especially in early LOAD pathology specific hippocampal subregions are distinctly affected. Thus measuring miRNA in the total hippocampal formation and in cortical regions will most likely miss a number of changes and the authors should address this issue which may explain why miRNAs that has been previously associated with LOAD pathogenesis do not show up in their assay. Are the authors not surprised that they did not find key miRs that have been linked to LOAD previously such as miR29, as shown by the author's themselves but also other miRNAs

We agree with the referee that these are important issues. We extensively discuss the human sampling issue in the first paragraph of the discussion section and how it affects detection of miRNAs. The heterogeneity of the brain areas that were investigated is also another important issue to be taken into account when comparing our data to previous experiments. We advocate that profiling larger cohort of patients allows rigorous statistical testing. In our 2008 paper, only 10 samples were initially profiled and further work with brain material was focused on the correlation between miR-29 changes and changes in BACE1 level in human brains. The conclusion of that manuscript was that miR-29 regulates BACE1 expression and potentially contributes to alterations in BACE1 expression in the disease. More strong conclusions could not be made at that time. In the current manuscript, large sample size allows for rigorous statistical correction, and therefore allowing an unbiased approach to analyze the data. The microRNA which we find now can be considered as markers of the disease.

3. The authors should also acknowledge that the data is compromised by the lack of cell type specificity. Especially in the advanced B&B stages this is an issue since the number of neurons is severely decreased while inflammatory processes increase and thus most of the signal likely stems from glia cells. Changes may therefore not only be LOAD related but could simply reflect a more neuronal vs. more glia-like profile.

This is a very valid point and although only single cell analysis will allow to address this definitively, we have done our best to analyze this issue in more depth because our data set allows us to perform analysis on cell specific miRNAs over different Braak stages. We include figure 2C which clearly show that changes in miRNAs over the different Braak stages are not simply explained by aspecific cell loss. We also discuss this issue in more detail in the discussion section. "For instance, some of the miRNA changes may not only be LOAD related but could simply reflect a more neuronal vs. more glia-like profile. With regard to the hippocampus samples analyzed, we found increases of microglial-enriched miR-142-3p, miR-150-5p and miR-223-3p in LOAD. Furthermore, it is also impossible to exclude that loss of neurons contributes to decreased miRNA-132-3p. However, neuronal-enriched miRNAs decreased only (and relatively mildly) at late stages of disease in the prefrontal cortex, suggesting that neurodegeneration can be an explanation for loss of miRNAs only at those late stages. Several miRNAs, such as those found in cluster 1 and including miR-132-3p, emerged start being deregulated already at early Braak stages before the appearance of major neuronal loss…."

4. The statistically analysis should be described in greater detail better. The binominal testing is suitable but probably not the best method to detect changes across the different stages of AD.

We compared the negative binomial model used in the paper to another negative binomial baes model which is less conservative in nature. We also compared it to a recently developed transformation i.e log-cpm values that generally results in a decreasing mean-variance trend when the count size increases. The overlap of the three methods is shown in Supplementary Figure 4 and clearly shows that, except for one miRNA, all the others are also found by another statistical method.

5. FIG 5: The authors should quantify miR132 levels in pTau positive vs. pTau negative cells of the same individual to strengthen their interpretation.

Quantification is shown in Figure 7H.

6. Fig. 6: Where does the array data for the gene-expression used in Fig 6A come from?

*The expression data used in the manuscript come from the publication: Concerted changes in transcripts in the prefrontal cortex precede neuropathology in Alzheimer's disease. **Bossers K**, Wirz KT, Meerhoff GF, Essing AH, van Dongen JW, Houba P, Kruse CG, Verhaagen J, Swaab DF. Brain. 2010 Dec;133(Pt 12):3699-723. doi: 10.1093/brain/awq258. Epub 2010 Oct 1.PMID:20889584 [PubMed - indexed for MEDLINE]*

This reference is presented in the manuscript.

7. The authors should analyze via qPCR if mRNA and via IHC or western blot if protein levels change as predicted for the target scan derived candidates. At least some of the key players such as SIRT1, EP3000, RB1, MAPK1, MAPK3 GSK3b should be analyzed.

Our paper highlights the importance of FOXO1 in the Tau pathway. We show by western-blot that this miR-132 target is increased at the protein level in the LOAD hippocampus. Several of the other targets have been studied in the past, but we did not obtain suitable antibodies to verify all candidates. We have therefore for now focused on FOXO1 and hope that the additional data with deep sequencing, luciferase construct analysis and so on convince the referee of the quality of our manuscript.

Additional comments:

With respect to the point 1 raised by reviewer 3 my opinion would be that it is certainly beyond the scope of the study to investigate another neurodegenerative disease in the depth as it has been done by the authors. This relates most of all to the amount of samples analyzed.

However, If I am correct referee 3 raises here as similar point that I have brought up. Especially at later stages of AD there are inflammatory processes that may account for the observed changes since the ratio of neuronal vs. glia cells severely changes.

As indicated above we have included analysis of MS samples and also analysis of specific miRNAs over different Braak stages (Fig 2C). We hope that these novel data partially answer to the issues raised by the referees. We also carefully discuss these issues in the discussion of the manuscript.

As for the 2nd comment on the controls of the luciferase assay. This would of course be a nice control that is commonly done in the field of miRNA research. Nevertheless, in the context of and/or protein levels of - at least some of -the predicted targets change accordingly in the human samples.

We have included the luciferase control experiments as required and also protein expression analysis of Foxo 1a as mentioned above.

Reviewer 3 comments:

Referee #3 (Comments on Novelty/Model System):

This is certainly an interesting study in an evolving field. However, authors only compared control vs. AD brains. Thus, it remains unclear whether the observed changes in individual miRNAs are disease specific or rather result from other more general pathogenic processes observed in other brain diseases as well, e.g. inflammation, oxidative stress, neurodegeneration..

We profiled a cohort of multiple sclerosis samples and did not detect change of miR-132 expression level in the active nor chronic lesions. This suggests that miR-132 deregulation is not a direct result of the inflammation. However miR-132 was shown by others to be deregulated in other neurodegenerative disorders such as schizophrenia, progressive supranuclear palsy, Huntington's

disease and frontotemporal lobar degeneration with TDP-43 inclusions. We made these two points clear in our discussion.
"Deregulation of miR-132-3p was not however observed in the small cohort of multiple sclerosis patients analyzed here, in agreement with a previous study done with a larger cohort of 30 MS patients/control subjects."
"Recently, several neurodegenerative diseases have been also characterized by alteration of miR-132-3p expression, including schizophrenia, progressive supranuclear palsy, Huntington's disease and frontotemporal lobar degeneration with TDP-43 inclusions (FTLD-TDP)."
We also included an analysis of expression of miRNAs typically expressed in neurons, glia or microglia cells (fig 2C) further providing specificity to the change in miR-132 we describe here.

Referee #3 (Remarks):

This is an interesting and well performed study describing differences of miRNAs between control and AD post-mortem brains.
Authors used well characterized brain samples from two brain banks and compared miRNA levels of late onset AD brains to age and sex matched controls. miRNAs were profiled with nCounter assay. Bioinformatical processing of the data revealed significant differences in several miRNA in hippocampal and prefrontal cortical areas. In the hippocampus samples, levels of miR-128, -138,-124,-129 were lower, while that of others, including let-7f, -7i, miR-23a,-142-3 and others were increased in AD.
In prefrontal cortical areas mainly other miRNAs were altered, with only little overlap as compared to the hippocampal region.
The alteration of miRNA levels was then validated by RT-PCR. Interestingly some alterations of miRNA levels correlated with Braak stages of tau pathology.
Authors then followed up in more detail miR132 expression by in situ hybridization as it showed a strong negative correlation to Braak stageing. Finally, potential targets of miRNA-132 were found to be connected by network analysis, some also known to be implicated in AD, including tau, gsk3beta and sirt1, and expression validated in hek293 cells.
Overall, this is a very interesting and well controlled study. The strength of the study clearly lies in the relatively high number of brain samples and also inclusion of functional analysis. However, I have a major concern about the specificity of the detected changes for late-onset AD. As brains of other disease conditions have not been included in the study, it remains unclear whether the changes in the miRNA levels were disease specific or rather caused by more general processes in brains undergoing degeneration or chronic inflammation. Indeed, miR-132 for example appears to regulate inflammatory processes.
I would like to encourage the authors to extend the study to at least one additional neurodegenerative/neuroinflammatory disease to assess the specificity of miRNA alterations for late onset AD.

As indicated above, we included the analysis of a series of MS samples. Furthermore the analysis of cell specific miRNA expression over the disease progression according to Braak stages reassures us that at least some of the changes (including miR-132) are related to disease process and not to general alterations in cell composition of the investigated material.

In conclusion, we have tried to answer all criticisms. We thank the three referees for their very helpful and constructive input and we feel that our work considerably improved thanks to this.

2nd Editorial Decision 24 July 2013

Thank you for the submission of your revised manuscript to EMBO Molecular Medicine. We have now received the enclosed report from the referee who was asked to re-assess it. As you will see the reviewer is now supportive and I am pleased to inform you that we will be able to accept your manuscript as soon as you will add the GEO accession number for the RNAseq experiment within the Materials and Methods.

I look forward to receiving a new revised version of your manuscript as soon as possible.

***** Reviewer's comments *****

Referee #3 (Comments on Novelty/Model System):

Authors addressed to points of the reviewer adequately.

© EMBO
