# Supplementary material for: Alteration of the microRNA network during the progression of Alzheimer's disease
Source: EMBO Mol Med. 2013 Sep 9;5(10):1613–34. doi: 10.1002/emmm.201201974 (PMC3799583; doi:10.1002/emmm.201201974)
Supplement: Supplementary file 2 [file emmm0005-1613-SD2.pdf]

SUPPLEMENTARY INFORMATION:

TABLE OF CONTENT

1/ Supplementary Figure legends and Supplementary Figures

Supplementary Figure 1. Quality assessment of the total RNA and distribution of the nCounter raw data

Supplementary Figure 2. Comparison of the raw data from hippocampus samples showing different RIN values

Supplementary Figure 3. Skewness and overdispersion of the nCounter data

Supplementary Figure 4. Multiple-group comparison of the prefrontal cortex data obtained with the nCounter system

Supplementary Figure 5. Comparison of deregulated miRNAs in diverse brain areas of LOAD patients

Supplementary Figure 6. Co-targets of miR-132-3p and the FOX transcription factors

2/ Supplementary Tables

Supplementary Table 1. Clinical diagnosis of cohort 1 (hippocampus samples)

Supplementary Table 2. Clinical data of sample cases in the cohort 2 (prefrontal cortex samples)

Supplementary Table 3. Clinical diagnosis of sample cases in the cohort 3 (temporal gyrus samples)

Supplementary Table 4. MiRNAs called different by deep-sequencing and without corresponding probes in the nCounter system

Supplementary Table 5. Percentage of neurons positive for miR-132-3p in the prefrontal cortex

Supplementary Table 6. miR-132-3p predictions for Tau related targets and FOX targets by different algorithms

Supplementary Table 7. Enrichment of transcription factor binding sites in the promoter of genes down-regulated between BRI and BRVI

Supplementary Table 8. Detection of FOX motifs by HOMER (known motif approach) in the different gene sets

Supplementary Table 9. List of PCR primers used for 3'UTR cloning

SUPPLEMENTARY FIGURE LEGENDS

Supplementary Figure 1. Quality assessment of the total RNA and distribution of the nCounter raw data

A/ RIN density plot of hippocampus and prefrontal cortex samples

The RIN values of the 64 hippocampus samples show alteration of RNA integrity in the vast majority of samples (blue density plot). RIN values are from 2.3 (lowest quality) to 7.4 (highest quality), with median RIN=3.8. The RIN values of the 49 prefrontal cortex samples (red density plot) are high, varying from 5.6 (lowest quality) to 9.1 (highest quality). Median RIN of prefrontal cortex samples=7.6.

B/ Distribution of the raw counts from hippocampus samples

The raw data of the 64 hippocampus samples were log2-transformed and boxplots represent global miRNA expression for each sample grouped according to their RIN values. From left to right, samples were ranked from highest to lowest total RNA quality. The number of samples in each color group is: red= 9 samples with 6<RIN<7.4, yellow= 11 samples with 5<RIN<6, blue= 11 samples with 4<RIN<5, purple= 14 samples with 3<RIN<4 and green= 19 samples with 2<RIN<3. Samples with lowest RIN values show similar distribution to those with highest RIN values.

C/ Distribution of the raw counts from prefrontal cortex samples

The raw counts of the 49 prefrontal cortex samples were log2-transformed and distribution of miRNA expression for each sample is shown by boxplot. Samples were stratified based on their RIN values. From left to right, samples were from highest to lowest quality. The color code is: red= 19 samples with 8<RIN<9.1, yellow= 22 samples with 7<RIN<8 and blue= 8

samples with 5.6<RIN<7. Distributions from these high-quality prefrontal cortex samples are similar to those found for hippocampus samples (see panel B).

Supplementary Figure 2. Comparison of the raw data from hippocampus samples showing different RIN values

A/ Pairwise comparisons of highest and lowest quality hippocampus samples

The raw counts of 10 samples with lowest RIN values (X1H, X33H, X39H, X40H, X60H, X144H, X13H, X24H, X26H, X7H) were compared to those of 10 samples with highest RIN values (X32H, X8H, X67H, X70H, X29H, X2H, X15H, X28H, X56H, X30H). Horizontal red line delimitates low and high quality samples grouped according to their RIN values. Pairwise comparisons were made by using scatterplots of log2-transformed raw counts (lower panel) and Pearson's correlation between each pair of samples was determined (upper panel). Pearson's correlation coefficient between any two hippocampus samples taken from each group was from 0.81 to 0.94 (green rectangle of the upper panel).

B/ Histograms of raw counts from hippocampus samples

Raw counts of hippocampus samples were log2-transformed and histograms for each sample represent the percentage of miRNAs found in each bin of log2-transformed expression values. The absence of a bell-shaped distribution function (histograms positively skewed), in addition to the discrete counting nature of the nCounter technology, precludes a statistical testing based on a normal distribution. The null hypothesis that raw data were normally distributed was formally rejected ($p=2.2E^{-16}$, Shapiro-Wilk test).

Supplementary Figure 3. Skewness and overdispersion of the nCounter data

A/ Histograms of raw counts from the prefrontal cortex samples

Raw counts of prefrontal cortex samples were log2-transformed and resulting histograms show positive skewness as observed for the hippocampus samples.

B/ Overdispersion of counts for the hippocampus samples

The miRNA counts possess characteristics of variance exceeding the mean (scatter smooth plot) and therefore show overdispersion with respect to a discrete Poisson distribution. For the theoretical Poisson distribution, the mean equals variance and dots should be scattered around the black line. As alternative to the Poisson model, a statistical model based on the negative binomial distribution was used to account for overdispersion when calling differential expression of miRNAs.

C/ Overdispersion of counts in the prefrontal cortex dataset

In this dataset, the variance was also higher than the mean, similar to what was observed for the hippocampus samples (panel B) and arguing for the use of a model based on the negative binomial distribution to determine which miRNAs were deregulated in LOAD prefrontal cortex.

Supplementary Figure 4. Multiple-group comparison of the prefrontal cortex data obtained with the nCounter system

A/ The number of differentially expressed miRNAs between any two Braak stages found by three different methods: DESeq (see main paper), edgeR (allowing a distinct dispersion for each miRNA "tagwise") and voom+Limma (after transformation to obtain a log2 counts per million value (cpm) for each miRNA and calculated as followed: cpm= log2 (((counts + 0.5) / (total number of counts + 1)) * 10^6). Overlaps between the different methods are also represented in the Venn diagram.

Supplementary Figure 5. Comparison of deregulated miRNAs in diverse brain areas of LOAD patients

A/ Comparison of miRNAs deregulated in the LOAD hippocampus

The up- and down-regulated hippocampal miRNAs reported by Cogswell et al. were compared to those found in the nCounter experiment. The old nomenclature was lifted to the miRBase V19 nomenclature. There was one up-regulated miRNA and four down-regulated miRNAs in common. Note the presence of miR-132-3p in common between the two experiments. Up-hippo= Up-regulated in the nCounter data, Up-Hippo-C: Up-regulated in Cogswell's study, Down-Hippo= Down-regulated in the nCounter data, Down-Hippo-C: Down-regulated in Cogswell's study.

B/ Comparison of miRNAs deregulated in the LOAD prefrontal cortex

The up and down-regulated prefrontal cortex miRNAs reported by Cogswell et al. were compared to the nCounter experiment. The old miRNA names were lifted to the ones found in miRBase V19. There was two down-regulated miRNAs found to be in common between the two studies including miR-210 and miR-132-3p. Up-prefrontal= Up-regulated in the nCounter data, Up-Prefrontal-C= Up-regulated in Cogswell's study, Down-Prefrontal= Down-regulated in the nCounter data, Down-Prefrontal-C: Down-regulated in Cogswell's study.

C/ Comparison of miRNAs deregulated in the hippocampus, parietal, anterior temporal cortex and grey matter of the temporal cortex

The up and down-regulated miRNAs found to be deregulated in the four diverse brain areas were directly extracted from the literature (see main paper for references). Data were not reanalyzed and results are shown as initially reported by the respective authors. Each brain area is represented by a rectangle and overlap between rectangles with number >0 indicates

the number of miRNAs found in common by the considered studies. Note the absence of miRNAs in common between the four studies. Parietal= red, Grey matter of temporal cortex= green, Hippocampus= purple, Anterior temporal cortex= blue.

Supplementary Figure 6. Co-targets of miR-132-3p and the FOX transcription factors

A/ Binding site motifs of FOX TFs predicted as miR-132-3p targets

Binding motifs of five of the six FOXs predicted by TargetScan to be miR-132-3p targets are shown by logos. Note the highly similar motifs for the five FOXs for which information was available in the queried databases. No motif information existed for FOXN3.

B/ Overlap between targets of miR-132-3p and FOXs

The promoters of the 407 miR-132-3p targets predicted by TargetScan were analyzed by iRegulon to find enriched regulatory motifs and by the ENCODE ChIP-Seq Significance Tool to identify enriched ENCODE TFs. Both tools were in accordance, with the enrichment of motifs for the FOX TFs in 204 of 407 predicted targets (iRegulon) and the binding of FOXP2 to 202 of the 407 miR-132-3p predicted targets. The overlap between the two tools was made of 95 predicted targets. When adding 93 up-regulated transcripts (BRVI compared to BRI) found to be enriched by Gene Set Enrichment Analysis (GSEA), an overlap of 31 transcripts was found, potentially being of relevance to LOAD.

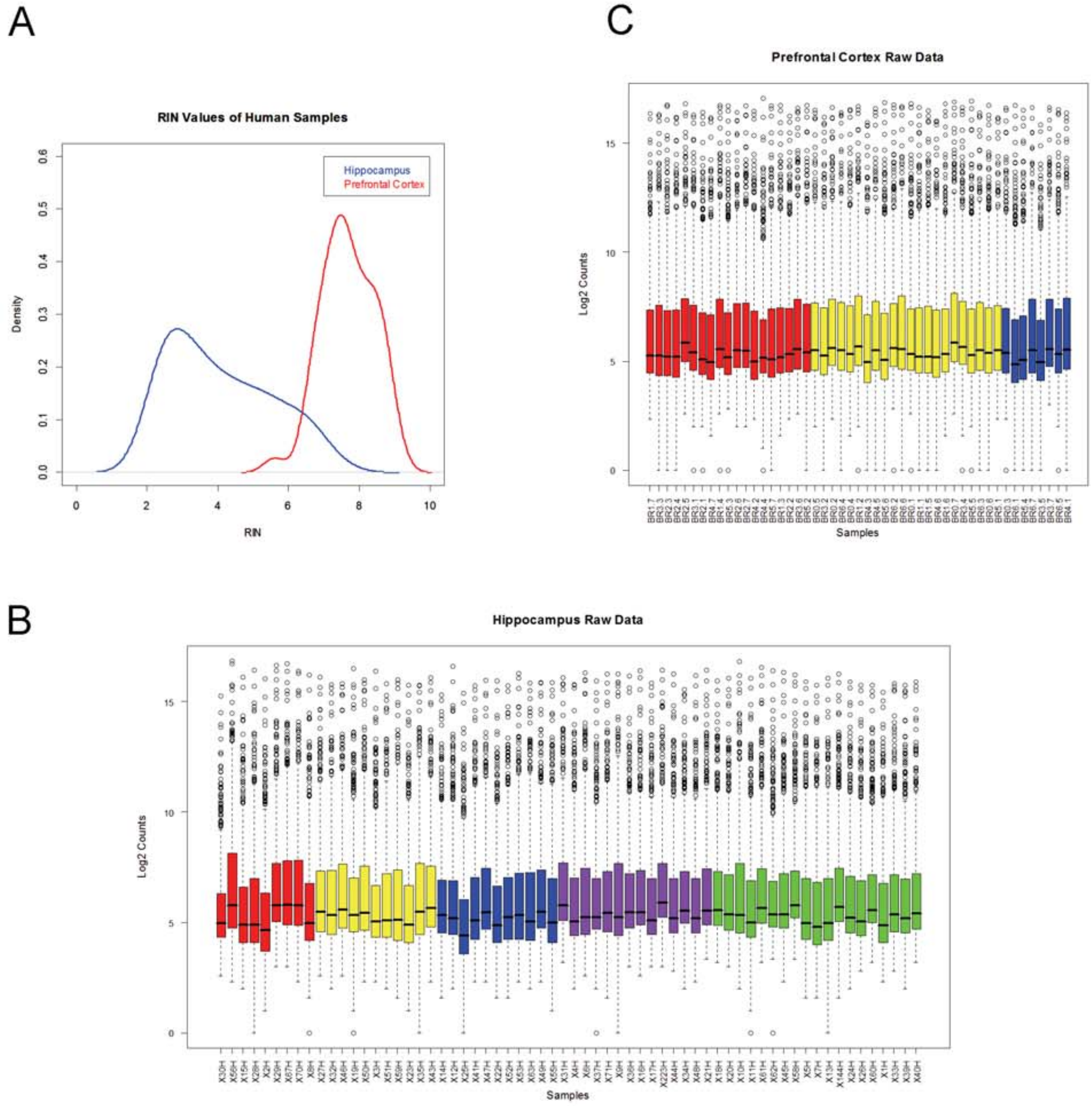

Supplementary Figure 1

Supplementary Figure 2

A

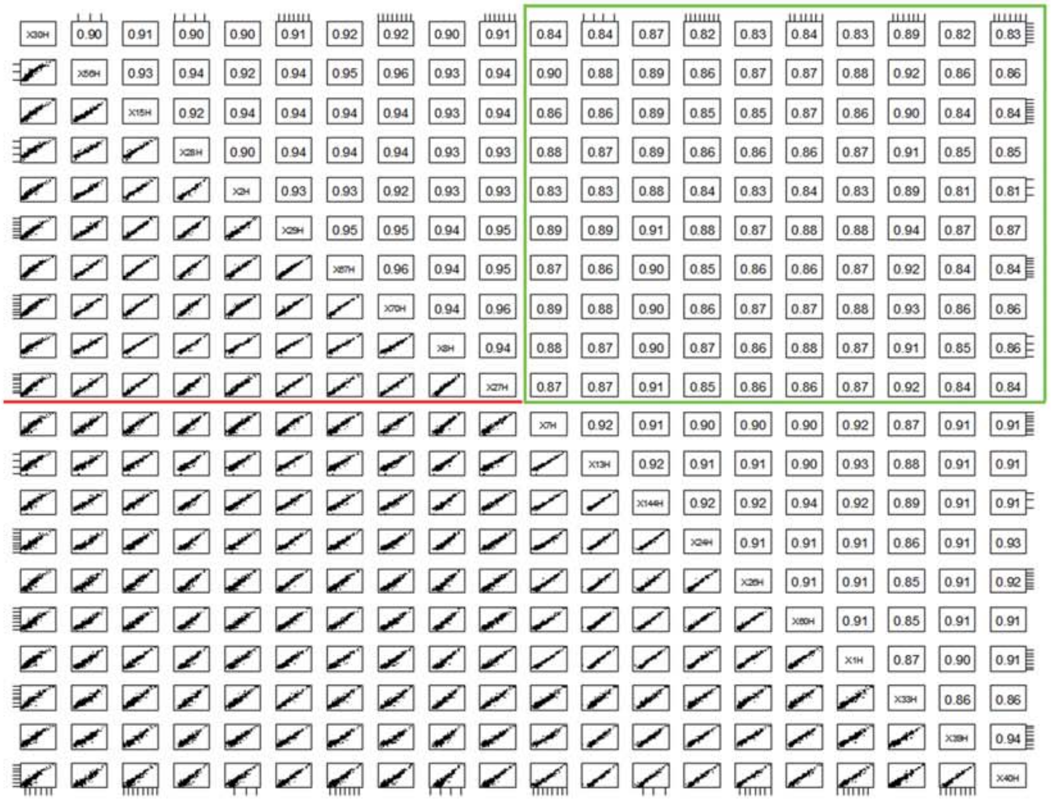

B

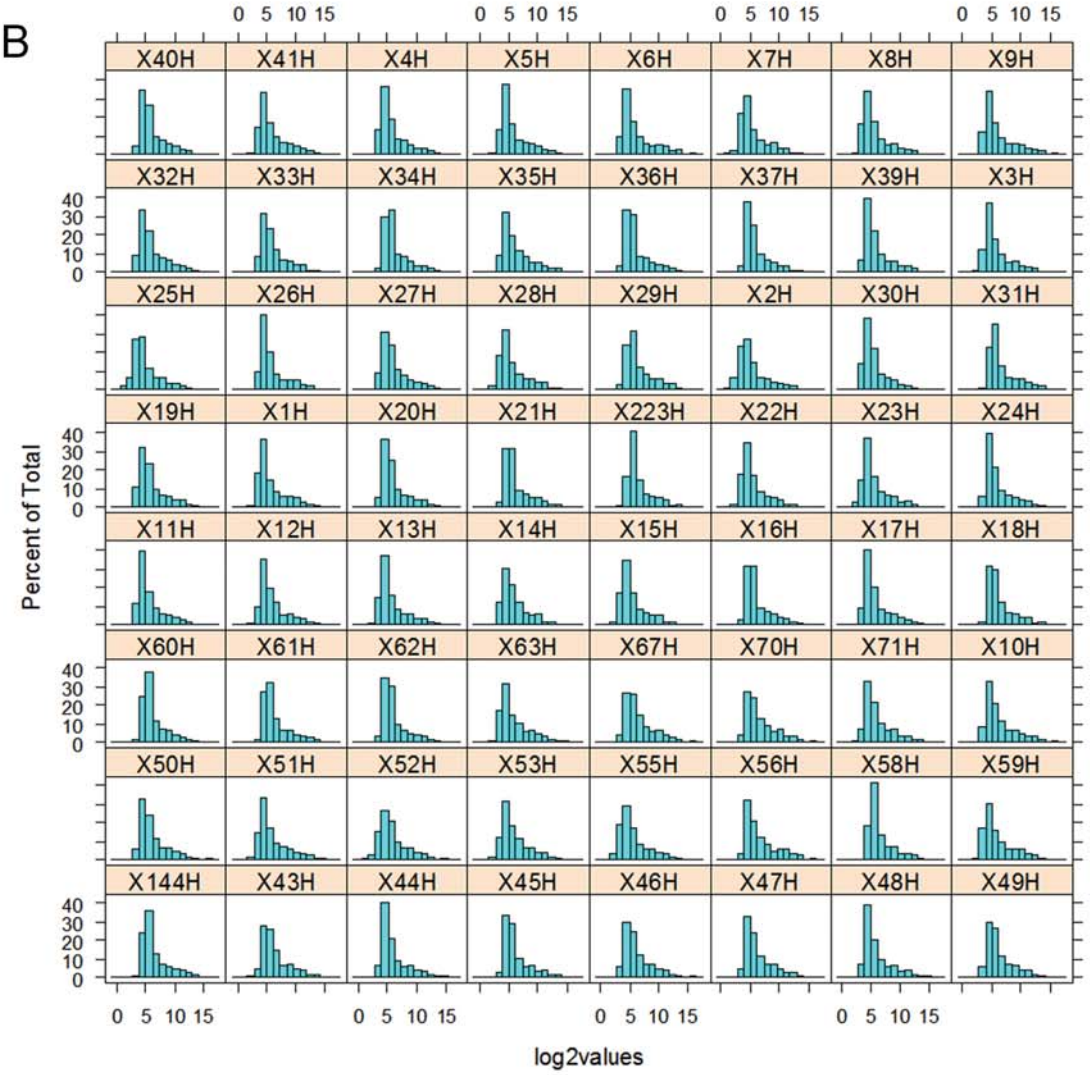

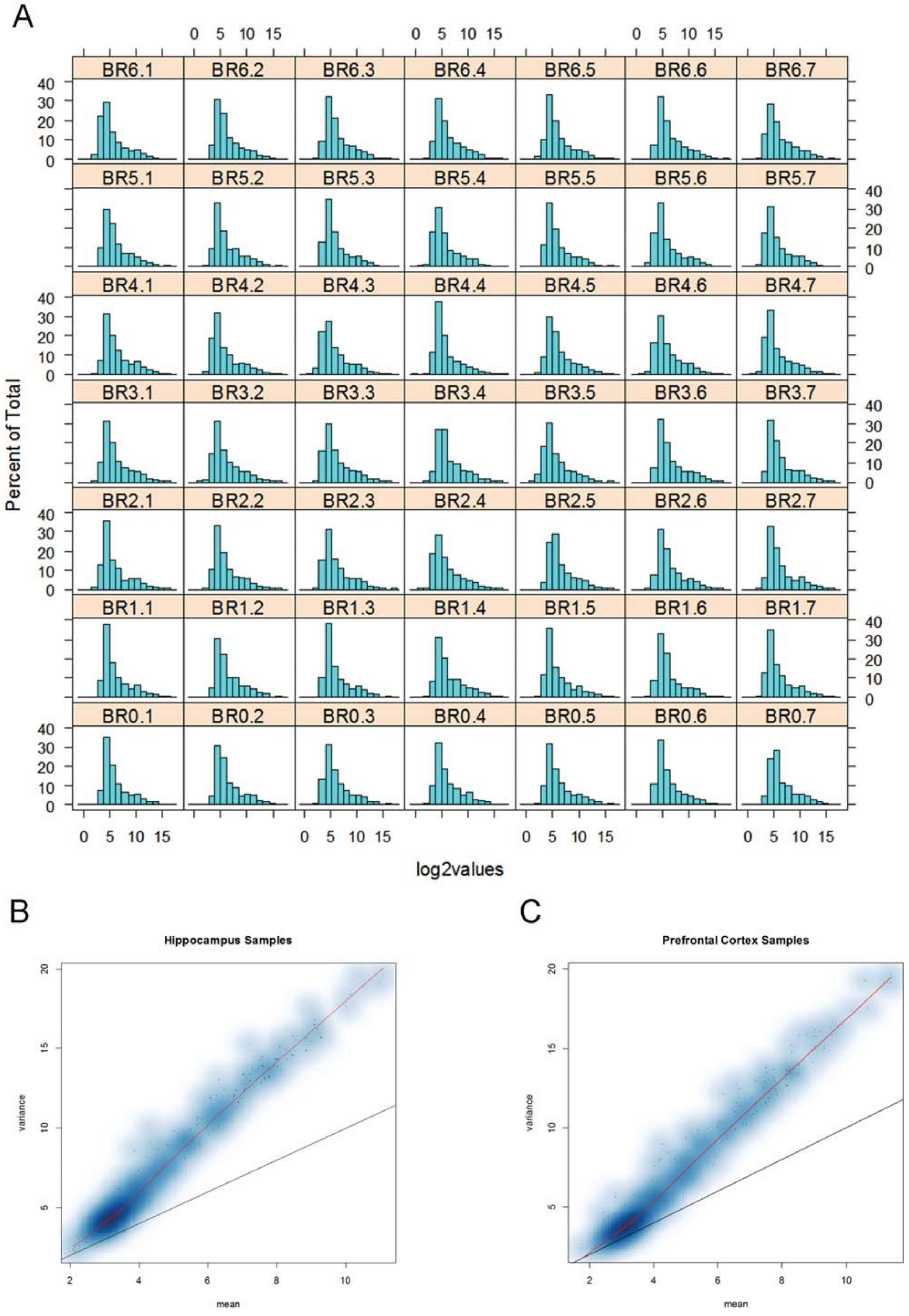

Supplementary Figure 3

Supplementary Figure 4

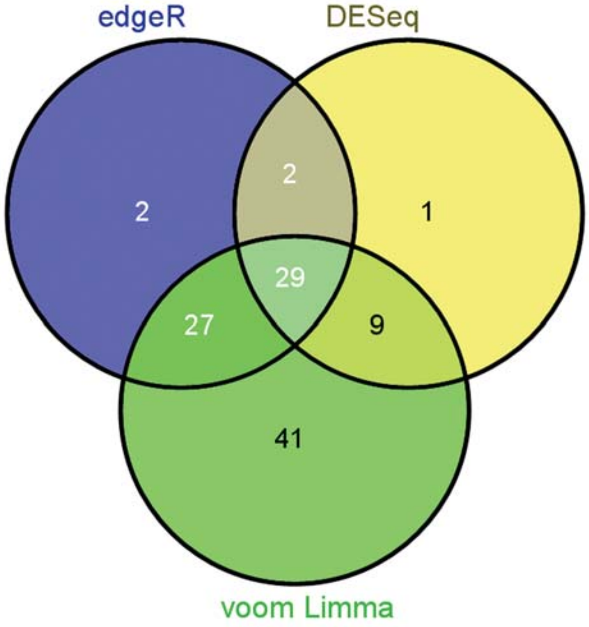

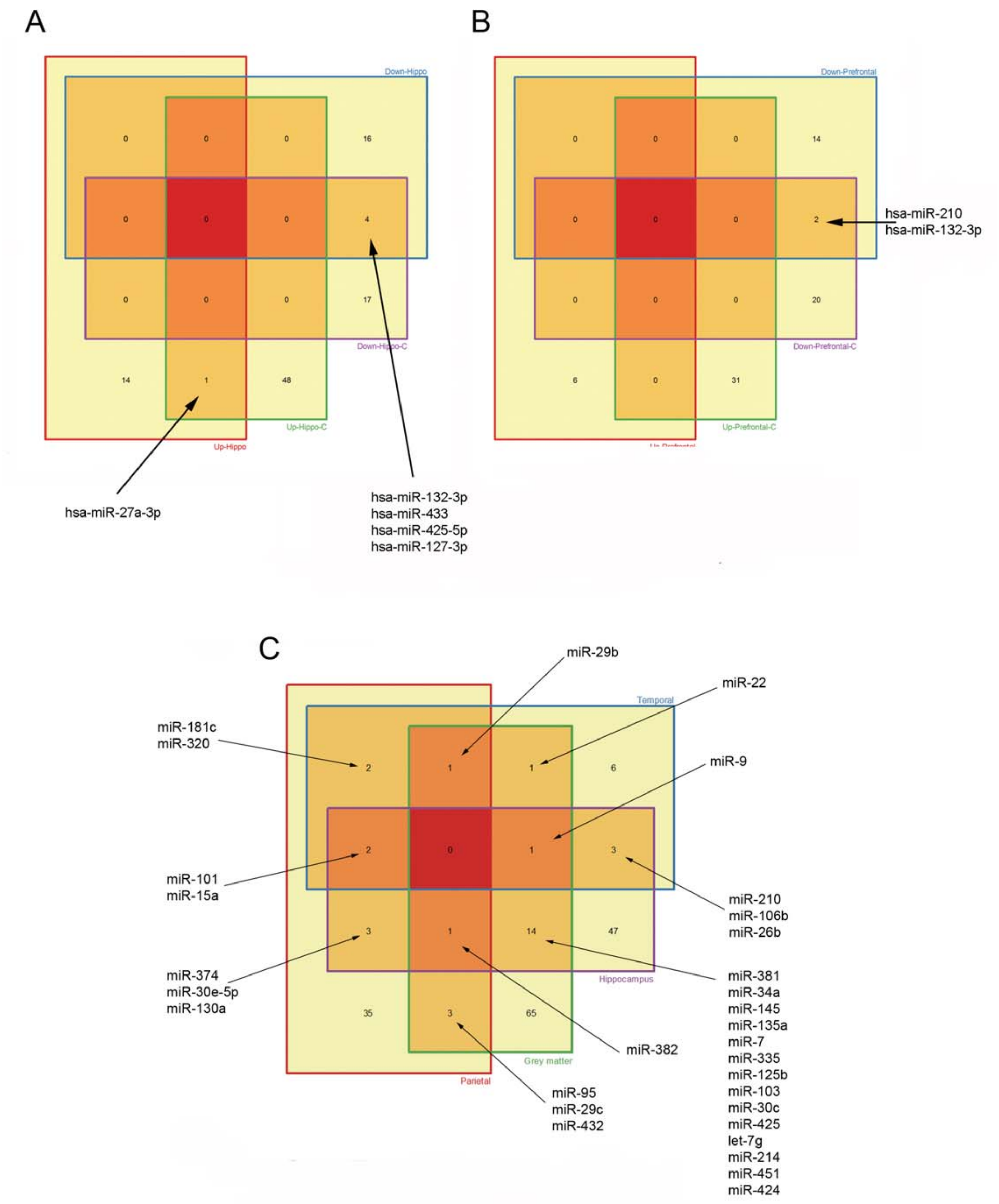

Supplementary Figure 5

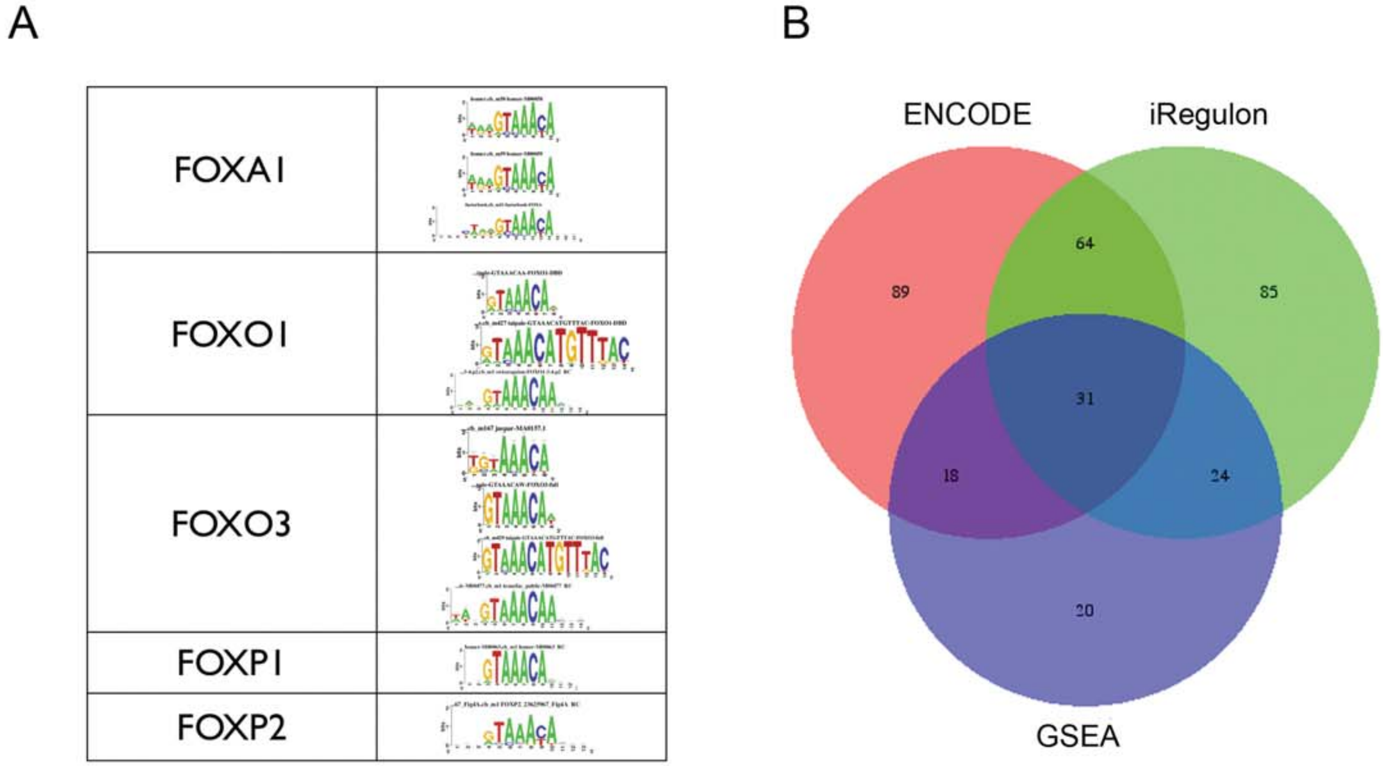

Supplementary Figure 6

Supplementary Table 1. Clinical diagnosis of cohort 1 (hippocampus samples)

ID	Brain region	Class	Sex	Age	PMI	Diagnosis 1	Diagnosis 2
X43H	hippocampus	Control	F	89	41	Normal	control case but with Hypoxic-type changes and amyloid angiopathy (AD BNE modified Braak score I-II)
X44H	hippocampus	Control	F	90	50	Leukemia	control brain- mild alzheimer-type changes (modified Braak stage II) and mild amyloid angiopathy
X45H	hippocampus	Control	M	66	52	normal	normal brain with minimal ageing changes, consistent with HP-tau stage +
X46H	hippocampus	Control	M	54	30.5	cancer	Normal brain parenchyma
X47H	hippocampus	Control	M	78	24	metastatic lesions	control - multiple deposits of metastatic carcinoma
X48H	hippocampus	Control	M	81	18	cerebral infarcts	control - old cerebral infarct (Braak I)
X49H	hippocampus	Control	M	78	30.5	normal brain	control BUT extensive amyloid angiopathy - no significant Alzheimer's pathology Braak I
X50H	hippocampus	Control	F	92	17	normal brain	control brain with some tau deposition
X51H	hippocampus	Control	F	80	3	cancer	minimal ageing changes, consistent with HP-Tau stage I; childhood poliomyelitis
X52H	hippocampus	Control	F	55	12	Mild atrophy	minimal tau pathology consistent with HP-tau stage I
X53H	hippocampus	Control	M	80	49	Normal brain	Consistent with ageing
X55H	hippocampus	Control	M	37	27	ALCOHOLIC CIRROHSIS	Normal adult brain
X56H	hippocampus	Control	M	54	50	ACUTE RESPIRATORY FAILURE	Normal adult brain
X58H	hippocampus	Control	M	41	32	ACUTE PANCREATITIS	Normal adult brain
X59H	hippocampus	Control	M	42	27	PERITONITIS	Normal adult brain
X60H	hippocampus	Control	M	59	50	Cancer	NORMAL ADULT BRAIN (NO SIGNIFICANT PATHOLOGY)
X61H	hippocampus	Control	F	82	43	Cancer	Normal adult brain
X62H	hippocampus	Control	M	79	24	SEPTICAEMIA	MILD AGEING CHANGES
X63H	hippocampus	Control	M	55	24	SYRINGOMYELIA	Normal adult brain
X67H	hippocampus	Control	F	81	49	CHRONIC ISCHAEMIC HEART DISEASE	Normal adult brain
X70H	hippocampus	Control	M	85	42	GASTROINTESTINAL HAEMORRHAGE	Normal adult brain
X71H	hippocampus	Control	F	64	60	CARCINOMA OF BLADDER	Normal adult brain
X144H	hippocampus	Control	F	92	22.5	Normal	Amyloid angiopathy. Suitable as control for MND and tau pathology in AD (Braak I-II) but some A-beta vascular pathology
X10H	hippocampus	LOAD	M	55	18.25	ALZHEIMER'S DISEASE: DEFINITE	None
X11H	hippocampus	LOAD	F	88	6	ALZHEIMER'S DISEASE	None
X12H	hippocampus	LOAD	F	96	19	Alzheimer's disease Braak V	severe amyloid angiopathy
X13H	hippocampus	LOAD	F	79	19.3	ALZHEIMER'S DISEASE	None
X14H	hippocampus	LOAD	M	76	22	Alzheimer's disease Braak VI	severe amyloid angiopathy, ischaemic lesions in cortex
X15H	hippocampus	LOAD	F	90	17	ALZHEIMER'S DISEASE	None
X16H	hippocampus	LOAD	F	90	16	ALZHEIMER'S DISEASE: DEFINITE	AMYLOID ANGIOPATHY
X17H	hippocampus	LOAD	F	64	9.5	ALZHEIMER'S DISEASE: DEFINITE	None
X18H	hippocampus	LOAD	M	71	5.25	Alzheimer's disease Braak V/VI	None
X19H	hippocampus	LOAD	F	84	<24	Alzheimer's disease Braak 5	None
X1H	hippocampus	LOAD	M	70	12	ALZHEIMER'S DISEASE	None
X20H	hippocampus	LOAD	F	81	24	Alzheimer's disease Braak VI?	None
X21H	hippocampus	LOAD	F	79	24	Alzheimer's disease Braak VI Hippocampal sclerosi	moderate amyloid angiopathy mild vascular pathology old cystic infarct
X223H	hippocampus	LOAD	F	81	24	ALZHEIMER'S DISEASE	None
X22H	hippocampus	LOAD	F	90	23	Alzheimer's disease	Infarction evidence of vasculitis
X23H	hippocampus	LOAD	M	64	10	ALZHEIMER'S DISEASE	None
X24H	hippocampus	LOAD	F	84	12	Alzheimer's disease Braak 5-6	amyloid angiopathy
X25H	hippocampus	LOAD	M	68	18	ALZHEIMER'S DISEASE: DEFINITE	None
X26H	hippocampus	LOAD	M	89	18.5	ALZHEIMER'S DISEASE: DEFINITE	AMYLOID ANGIOPATHY (SEVERE)
X27H	hippocampus	LOAD	F	78	22	ALZHEIMER'S DISEASE	AMYLOID ANGIOPATHY
X28H	hippocampus	LOAD	F	79	8	ALZHEIMER'S DISEASE: DEFINITE	None
X29H	hippocampus	LOAD	F	66	21	ALZHEIMER'S DISEASE	None
X2H	hippocampus	LOAD	F	83	20	ALZHEIMER'S DISEASE	None
X30H	hippocampus	LOAD	M	74	24	ALZHEIMER'S DISEASE	None
X31H	hippocampus	LOAD	M	81	24	ALZHEIMER'S DISEASE	None
X32H	hippocampus	LOAD	M	84	21	ALZHEIMER'S DISEASE	CEREBRAL VASCULAR DISEASE (SMALL INFARCTS), ARACHNOID CYST
X33H	hippocampus	LOAD	F	84	23	ALZHEIMER'S DISEASE	CEREBROVASCULAR DISEASE
X34H	hippocampus	LOAD	F	88	19	Alzheimer's disease with amyloid angiopathy Braak V	mild Small Vessel disease
X35H	hippocampus	LOAD	F	75	14	ALZHEIMER'S DISEASE	None
X36H	hippocampus	LOAD	M	63	20	ALZHEIMER'S DISEASE	None
X37H	hippocampus	LOAD	F	91	9	Alzheimer's disease	None
X39H	hippocampus	LOAD	F	?	24	ALZHEIMER'S DISEASE	None
X3H	hippocampus	LOAD	F	82	19	ALZHEIMER'S DISEASE	None
X40H	hippocampus	LOAD	M	67	24	ALZHEIMER'S DISEASE	None
X41H	hippocampus	LOAD	F	89	20	Alzheimer's disease tangle predominant type Braak V	None
X4H	hippocampus	LOAD	F	96	18	Alzheimer's disease with amyloid angiopathy Braak V	Mild Small Vessel disease
X5H	hippocampus	LOAD	F	84	16.6	Alzheimer's disease Braak V severe	vascular changes
X6H	hippocampus	LOAD	M	85	17	ALZHEIMER'S DISEASE	None
X7H	hippocampus	LOAD	M	78	20.5	Alzheimer's disease Braak VI	amyloid angiopathy
X8H	hippocampus	LOAD	F	69	16.3	Alzheimer's disease Braak VI	None
X9H	hippocampus	LOAD	F	71	21	Alzheimer's disease Braak VI	severe amyloid angiopathy

Sex: F (Female), M (Male)
PMI: *post-mortem* interval (in hours)

Supplementary Table 2. Clinical data of sample cases in the cohort 2 (prefrontal cortex samples)

		Braak stage		Prefrontal patholoy				
ID	Group	NFT	PL	NFT	PL	Age	Sex	APOE
BR01	Non-demented control, Braak 0	0	B	None	Substantial number of diffuse plaques	64	F	42
BR02	Non-demented control, Braak 0	0	A	None	None	80	M	33
BR03	Non-demented control, Braak 0	0	A	None	Some senile plaques, few diffuse plaques	72	M	43
BR04	Non-demented control, Braak 0	0	A	None	None	78	M	33
BR05	Non-demented control, Braak 0	0	A	None	None	52	F	33
BR06	Non-demented control, Braak 0	0	B	None	Many diffuse plaques	74	M	43
BR07	Non-demented control, Braak 0	0	A	?	?	74	F	33
BR11	Non-demented control, Braak 1	I	A	None	None	72	F	43
BR12	Non-demented control, Braak 1	I	B	None	Few senile plaques, many diffuse plaques	83	M	33
BR13	Non-demented control, Braak 1	I	A	A single NFT	Few diffuse plaques	78	F	43
BR14	Non-demented control, Braak 1	I	A	None	None	78	M	33
BR15	Non-demented control, Braak 1	I	A	None	Few plaques, few classic	90	F	32
BR16	Non-demented control, Braak 1	I	A	None	Few-to-many plaques, partly classical	79	M	33
BR17	Non-demented control, Braak 1	I	A	None	Substantial number of plaques, few classical	82	F	32
BR21	Non-demented control, Braak 2	II	A	None	None	68	M	33
BR22	Non-demented control, Braak 2	II	A	None	None	79	M	33
BR23	Non-demented control, Braak 2	II	A	None	None	78	F	43
BR24	Non-demented control, Braak 2	II	C	None	None	89	F	32
BR25	Non-demented control, Braak 2	II	B	None	Many plaques	69	F	43
BR26	Non-demented control, Braak 2	II	B	None	Few-to-substantial senile plaques	71	M	43
BR27	Non-demented control, Braak 2	II	O	None	None	83	F	33
BR31	Non-demented control, Braak 3	III	B	None	Many senile plaques	87	M	33
BR32	Dementia with s.i.c.c., Braak 3	III	B	None	Many plaques	74	F	43
BR33	Non-demented control, Braak 3	III	B	None	Many plaques	81	M	33
BR34	Non-demented control, Braak3	III	B	None	Substantial senile plaques	93	M	43
BR35	Non-demented control, Braak 3	III	B	None	Many diffuse plaques, some classical. Few senile plaques	84	F	33
BR36	Non-demented control, Braak 3	III	A	None	None	91	F	32
BR37	Dementia with s.i.c.c., Braak3	III	C	Few NFTs	Many plaques, substantial senile plaques	85	F	33
BR41	Alzheimer's disease, Braak 4	IV	C	None	Many plaques, few senile plaques	86	F	43
BR42	Alzheimer's disease, Braak 4	IV	C	None	Many largely diffuse plaques, several senile plaques	88	F	33
BR43	Alzheimer's disease, Braak 4	IV	C	Few NFTs	Many plaques, few classic plaques, few senile plaques	86	M	33
BR44	Alzheimer's disease, Braak 4	IV	C	Few NFTs	Many plaques, substantial senile plaques	75	F	33
BR45	Alzheimer's disease, Braak 4	IV	C	None	Many plaques, many senile plaques	79	M	43
BR46	Alzheimer's disease, Braak 4	IV	C	Variable NFTs	Many diffuse plaques	78	M	43
BR47	Alzheimer's disease, Braak 4	IV	B	Few NFTs	Many diffuse plaques, few senile plaques	84	F	32
BR51	Alzheimer's disease, Braak 5	V	C	Many NFTs	Many senile plaques	71	F	43
BR52	Alzheimer's disease, Braak 5	V	C	Many NFTs	Many senile plaques	69	F	42
BR53	Alzheimer's disease, Braak5	V	C	Substantial NFTs	Many plaques, part classical, few senile plaques	85	M	43
BR54	Alzheimer's disease, Braak 5	V	C	Many NFTs	Many diffuse plaques, many senile plaques	67	M	33
BR55	Alzheimer's disease, Braak 5	V	C	Few NFTs	Substantial plaques, few senile plaques	78	M	43
BR56	Alzheimer's disease, Braak 5	V	C	Many NFTs	Substantial senile plaques	71	F	33
BR57	Alzheimer's disease, Braak 5	V	C	Many NFTs	Many plaques, some senile plaques	79	M	33
BR61	Alzheimer's disease, Braak 6	VI	C	Substantial NFTs	Many plaques, many senile plaques	80	M	43
BR62	Alzheimer's disease, Braak 6	VI	C	Many NFTs	Many plaques, many senile plaques	80	F	44
BR63	Alzheimer's disease, Braak 6	VI	C	Substantial NFTs	Many plaques, few senile plaques	67	M	43
BR64	Alzheimer's disease, Braak 6	VI	C	Many NFTs	Many plaques, many senile plaques	67	F	33
BR65	Alzheimer's disease, Braak 6	VI	C	Many NFTs	Many plaques, many senile plaques	68	F	32
BR66	Alzheimer's disease, Braak 6	VI	C	Many NFTs	Many senile plaques	58	M	33
BR67	Alzheimer's disease, Braak 6	VI	C	Many NFTs	Many plaques, many senile plaques	72	F	43

NFT: presence of neurofibrillary tangles disseminated in major cortical brain areas according to Braak stages (O to VI) and in prefrontal cortex
PL: presence of amyloid plaque loads in major cortical brain areas and in prefrontal cortex (From A to C)
Sex: F (Female), M (Male)
APOE: APOE genotype

Supplementary Table 3. Clinical diagnosis of sample cases in the cohort 3 (temporal gyrus samples)

Class	ID	Sex	Age	Braak	PMI	APOE	Brain region
Alzheimer's disease	NBB1	M	77	VI	06:35	NA	superior temporalis gyrus
Alzheimer's disease	NBB2	M	80	VI	04:20	43	inferior temporalis gyrus
Alzheimer's disease	NBB4	M	82	V	04:15	43	medial temporalis gyrus
Alzheimer's disease	NBB5	M	83	VI	06:10	NA	superior temporalis gyrus
Alzheimer's disease	NBB7	M	85	V	07:10	33	medial temporalis gyrus
Alzheimer's disease	NBB8	M	85	V	04:25	43	superior temporalis gyrus
Alzheimer's disease	NBB9	M	85	V	04:45	43	superior temporalis gyrus
Alzheimer's disease	NBB10	M	86	V	06:15	NA	superior temporalis gyrus
Non-demented control	NBB14	M	71	I	07:40	33	superior temporalis gyrus
Non-demented control	NBB15	M	77	I	08:25	32	superior temporalis gyrus
Non-demented control	NBB16	M	78	I	05:35	43	inferior temporalis gyrus
Non-demented control	NBB17	M	80	0	07:15	33	superior temporalis gyrus
Non-demented control	NBB19	M	82	I	05:10	NA	superior temporalis gyrus
Non-demented control	NBB20	M	84	I	07:05	33	superior temporalis gyrus
Non-demented control	NBB21	M	88	I	07:00	32	superior temporalis gyrus
Non-demented control	NBB22	M	96	I	06:30	33	superior temporalis gyrus

M (Male)

PMI: *post-mortem* interval (in hours)

APOE: APOE genotype

NA: Not available

Supplementary Table 4. MiRNAs called different by deep-sequencing and without corresponding probes in the nCounter system

miRNA	Control	LOAD	Fold Change	log2 Fold Change	pval	padj
hsa-miR-132-5p	1315	339	0.26	-1.95	6.97E-13	1.95E-10
hsa-miR-3607-3p	3946	576	0.15	-2.78	1.08E-12	2.27E-10
hsa-miR-7-1-3p	166	432	2.60	1.38	6.46E-10	5.42E-08
hsa-miR-5701	217	46	0.21	-2.25	8.31E-10	6.34E-08
hsa-miR-338-3p	28741	65927	2.29	1.20	9.47E-10	6.62E-08
hsa-miR-26b-3p	435	174	0.40	-1.33	2.40E-09	1.55E-07
hsa-miR-3653	226	51	0.23	-2.14	8.27E-09	4.62E-07
hsa-miR-212-5p	197	51	0.26	-1.94	9.04E-09	4.74E-07
hsa-miR-6087	9	2	0.16	-2.61	2.69E-07	7.05E-06
hsa-miR-376a-5p	3336	1156	0.35	-1.53	5.02E-07	1.24E-05
hsa-miR-1225-5p	42	12	0.28	-1.83	5.25E-06	9.18E-05
hsa-miR-873-3p	531	244	0.46	-1.12	5.49E-06	9.40E-05
hsa-miR-6511b-3p	296	146	0.49	-1.02	7.77E-06	0.0001
hsa-miR-6511a-3p	262	130	0.49	-1.01	1.02E-05	0.0002
hsa-miR-365b-3p	195	380	1.95	0.97	1.30E-05	0.0002
hsa-miR-3613-5p	143	278	1.95	0.96	2.42E-05	0.0003
hsa-miR-6721-5p	8	2	0.24	-2.03	3.12E-05	0.0004
hsa-miR-4520a-3p	15	4	0.29	-1.80	3.53E-05	0.0004
hsa-miR-1185-1-3p	80	32	0.40	-1.31	4.93E-05	0.0005
hsa-miR-139-3p	397	216	0.54	-0.88	6.15E-05	0.0006
hsa-miR-3613-3p	18	44	2.44	1.28	7.18E-05	0.0007
hsa-miR-191-3p	142	73	0.51	-0.97	8.58E-05	0.0008

Numbers in the Control and LOAD columns represent normalized reads

Supplementary Table 5. Percentage of neurons positive for miR-132-3p in the prefrontal cortex

		Absolute counts			Percentage	
**ID**	**Braak**	**miR-132-3p(+)**	**miR-132-3p(-)**	**Total**	**miR-132-3p(+)**	**miR-132-3p(-)**
BR0.3	0	162	12	174	93.1	6.9
BR1.5	I	134	10	144	93.1	6.9
BR1.7	I	143	8	151	94.7	5.3
BR2.2	II	91	7	98	92.9	7.1
BR3.2	III	180	9	189	95.2	4.8
BR4.3	IV	125	2	127	98.4	1.6
BR5.2	V	163	12	175	93.1	6.9
BR6.5	VI	206	13	219	94.1	5.9

Supplementary Table 6. miR-132-3p predictions for Tau related targets and FOX targets by different algorithms

miRNA	Target Gene	TargetScan v6.2	DIANAmT	microrna.org	miRanda	miRDB	miRWalk	RNAhybrid	PICTAR4	PICTAR5	PITA	RNA22	sum
hsa-miR-132	EP300	1	1	1	1	1	1	1	1	1	1	0	10
hsa-miR-132	SIRT1	1	1	0	1	1	1	1	0	1	1	1	9
hsa-miR-132	MAPT	1	1	1	1	0	1	0	0	0	0	0	5
hsa-miR-132	GSK3B	1	0	0	1	0	1	0	0	0	0	0	3
hsa-miR-132	FOXN3	1	1	1	1	0	1	1	1	1	1	1	10
**hsa-miR-132**	**FOXO3**	**1**	**1**	**1**	**1**	**1**	**1**	**1**	**1**	**1**	**1**	**0**	**10**
hsa-miR-132	FOXA1	1	1	1	1	1	1	1	0	1	1	0	9
hsa-miR-132	FOXP2	1	1	1	1	1	1	0	0	1	1	0	8
hsa-miR-132	FOXP1	1	1	1	1	0	1	0	0	1	0	0	6
hsa-miR-132	FOXN4	0	1	0	1	0	1	0	0	1	0	1	6
hsa-miR-132	FOXM1	0	1	0	1	0	1	0	0	1	1	0	6
**hsa-miR-132**	**FOXO1**	**1**	**1**	**0**	**1**	**0**	**1**	**0**	**0**	**1**	**0**	**0**	**5**
hsa-miR-132	FOXE1	0	1	0	1	0	1	0	0	1	0	0	5
hsa-miR-132	FOXF2	0	1	0	1	0	1	0	0	1	0	0	5
hsa-miR-132	FOXG1	0	0	1	1	0	0	0	0	1	1	0	4
hsa-miR-132	FOXN2	0	0	1	1	0	0	0	0	1	1	0	4
hsa-miR-132	FOXJ3	0	0	0	1	0	0	0	0	1	1	0	4
hsa-miR-132	FOXK1	0	0	0	1	0	0	0	0	1	1	0	3
hsa-miR-132	FOXQ1	0	0	0	1	0	0	0	0	1	1	0	3
hsa-miR-132	FOXC1	0	0	0	1	0	0	0	0	1	0	0	2
hsa-miR-132	FOXJ2	0	0	0	1	0	0	0	0	1	0	0	2
hsa-miR-132	FOXK2	0	0	0	1	0	0	0	0	1	0	0	2
hsa-miR-132	FOXP4	0	0	0	1	0	0	0	0	1	0	0	2
**hsa-miR-132**	**FOXO4**	**0**	**0**	**0**	**0**	**0**	**0**	**0**	**0**	**1**	**0**	**0**	**1**
hsa-miR-132	FOXC2	0	0	0	0	0	0	0	0	0	1	0	1
hsa-miR-132	FOXD2	0	0	0	0	0	0	0	0	0	1	0	1

Eleven target prediction software were used to obtain miR-132-3p predictions for the four Tau related targets (EP300, SIRT1, MAPT and GSK3B)
The other miR-132-3p predictions are for the FOX transcripts and the FOXO subfamily is shown in bold
The sum corresponds to the number of different algorithms predicting a particular miR-132-3p target

Supplementary Table 7. Enrichment of transcription factor binding sites in the promoter of genes down-regulated between BRI and BRVI

Rank	Motif id	Z-score	Transcription factor	Target genes
1	elemento-TTTATGGC	3.61	NA	31
2	transfac_pro-M01862	3.56	ATF2,CREB1,CREM,ATF3,ATF4,ATF1,ATF7,ATF5,ATF6,JDP2	54
3	hdpi-CREB1	3.44	CREB1	47
4	transfac_pro-M00338	3.44	ATF3,ATF5,ATF1,CREB1,ATF7,CREM,ATF6,ATF2,ATF4,JUN,JDP2	98
5	transfac_pro-M00039	3.41	CREB1,CREM,ATF2,ATF6,ATF7,ATF1,ATF3,ATF4,ATF5,JDP2	112
6	transfac_pro-M00916	3.40	CREB1,CREM,ATF6,ATF1,ATF2,ATF7,ATF4,ATF5,ATF3,JDP2	148
7	yetfasco-299	3.34	ATF2,CREB1	80
8	yetfasco-44	3.30	ATF2,CREB1,CREM	83
9	yetfasco-8	3.30	ATF2,CREB1,CREM	83
10	transfac_pro-M00178	3.28	CREB1,CREM,ATF2,ATF4,ATF3,ATF5,ATF1,ATF7,ATF6,JDP2	91
11	transfac_pro-M00017	3.28	CREB1,CREM,ATF3,ATF2,ATF6,ATF4,ATF1,ATF7,ATF5,JDP2	149
12	jaspar-PF0014.1	3.23	ATF3,ATF1,CREB1,JDP2	28
13	elemento-TGACGTCA	3.23	ATF3,ATF1,CREB1,JDP2	95
14	stark-AATTRNNNNNCAA	3.22	NA	29
15	transfac_pro-M00179	3.22	ATF2,CREB1,CREM,ATF4,ATF3,ATF7,ATF6,ATF5,ATF1,E4F1	28
16	transfac_pro-M00177	3.20	CREB1,CREM,ATF4,ATF2,ATF3,ATF1,ATF6,ATF5,ATF7	136
17	transfac_pro-M01863	3.18	ATF3,ATF4,ATF2,CREB1,CREM,ATF7,ATF6,ATF1,ATF5	22
18	jaspar-PF0099.1	3.15	NA	33
19	yetfasco-1489	3.10	CREB1,CREM,ATF6,ATF5,ATF3,ATF7,ATF4,ATF2,ATF1	63
20	transfac_pro-M00406	3.04	MEF2A	43
21	jaspar-CN0010.1	3.04	RFX1,RFX2,RFX4,RFX6,RFX8	29
22	transfac_pro-M00691	3.04	ATF1,ATF3	81
23	transfac_pro-M00917	3.04	CREM,CREB1,ATF2,ATF6,ATF4,ATF1,ATF3,ATF5,ATF7,JDP2	148
24	stark-AATTRYGWCA	3.03	NA	16
25	transfac_pro-M01861	3.03	ATF1,ATF2,CREB1,ATF6,ATF7,ATF4,ATF5,CREM,ATF3	84
26	jaspar-PF0009.1	3.02	ATF3,JDP2	31
27	yetfasco-727	3.01	NA	33

There were 673 genes down-regulated between BRI and BRVI stages

NA: Motif not annotated

Supplementary Table 8. Detection of FOX motifs by HOMER (known motif approach) in the different gene sets

Homer parameters	Gene set	FOX rank	P-value	log P-pvalue	% of Targets	% of Background	Best Match/Details
Homer_20kb	Down	ND	ND	ND	ND	ND	ND
Homer_20kb	Up	2	1.00E-04	-1.12E+01	27.58%	21.41%	NF1:FOXA1/LNCAP-FOXA1-ChIP-Seq/Homer
Homer_20kb	Up	16	1.00E-02	-5.58E+00	100.00%	99.35%	Foxo1(Forkhead)/RAW-Foxo1-ChIP-Seq/Homer
Homer_20kb	TargetScan_miR-132-3p	6	1.00E-02	-4.98E+00	95.45%	86.81%	FOXP1(Forkhead)/H9-FOXP1-ChIP-Seq(GSE31006)/Homer
Homer_20kb	TargetScan_miR-132-3p	9	1.00E-02	-4.80E+00	44.32%	31.57%	FOXA1:AR/LNCAP-AR-ChIP-Seq/Homer

Down: Down-regulated in BRVI compared to BRI stage

Up: Up-regulated in BRVI compared to BRI stage

TargetScan_miR-132-3p: miRNA targets as predicted by TargetScan algorithm

The FOX rank indicates the rank of the motif predicted to be bound by the FOX TFs

ND: FOX motif not detected

Supplementary Table 9. List of PCR primers used for 3'UTR cloning

Name	Sequence (5' to 3')
TJAP1-XhoI	TGATCTCGAGGCCCCTGCTGGCCTTCCTGCCATTG
TJAP1-AscI	TGATGGCGCGCCATACCAGTAAAATAGTTTTATTTG
TJAP1-del-Reverse1	CTCAAAGCTTAAGAGTAGAGCCAAGGTTGGGAGT
TJAP1-del-Reverse2	AGTTTTATTTGATTTTAAAATAGTCATCAATGTGAAAATTTCTCAAAGCTTAAGAG
TJAP1-del-Reverse3	TGATGGCGCGCCATACCAGTAAAATAGTTTTATTTG
SIRT1-XhoI	TGATCTCGAGTGTAATAATTGTGCAGGTACAGG
SIRT1-AscI	TGATGGCGCGCCAAGTTAACAGAAAAAAGTCAAATGAC
SIRT1-del-SbfI	TGATCCTGCAGGTACAAAACATATGCCAGT
EP300-XhoI	TGATCTCGAGAGACACCTTGTAGTATTTTGGGAGC
EP300-AscI	TGATGGCGCGCCTGTCTGTCTCACACAGTTTATTTAAC
EP300-del-SbfI	TGATCCTGCAGGTATACAGTGATCCAAAGTTC
MAPT-XhoI	TGATCTCGAGTCAGGCCCCTGGGGCGGTCAATAATTG
MAPT-AscI	TGATGGCGCGCCAATCAGAGTAATAACTTTATTTC
MAPT-del-AscI	TGATGGCGCGCCAGTGTAAATCATTTGTTAAAACAC
FOXO1-XhoI	TGATCTCGAGTGGGGCAGCATTCATAATTTTCA
FOXO1-AscI	TGATGGCGCGCCTTGTGGCTGACAAGACTTAACTC
FOXO1-del1-Forward	GAGGCAACTACAGCCAAAATCAAATCTTACTACTTCATGGAG
FOXO1-del1-Reverse	CTCCATGAAGTAGTAAGATTTGATTTTGGCTGTAGTTGCCTC
FOXO1-del2-Forward-SbfI	TGATCCTGCAGGTCCGTAATATAGACGTATGGAA
FOXO1-del2-Reverse-SbfI	TGATCCTGCAGGACAAACCAGAGAAAATAAATCAA
